# Machine Learning Force Field Predictions of Structural and Dynamical Properties in HOPG Defects and the HOPG-Water Interface with Electronic Structure Analysis

**DOI:** 10.1021/acsomega.5c02543

**Published:** 2025-06-10

**Authors:** Mary T. Ajide, Parisa Naeiji, Joaquín Klug, Niall J. English

**Affiliations:** † School of Chemical & Bioprocess Engineering, 8797 University College Dublin, Belfield, Dublin 4, Ireland; ‡ Department of Life Sciences, 364010 Atlantic Technological University, ATU Sligo, Ash Lane, Sligo F91 YW50, Ireland

## Abstract

Understanding the electronic, structural, and dynamical properties of highly oriented pyrolytic graphite (HOPG) and its interface with water is crucial, as its layered structure, vacancy defects, and elemental doping offer untapped potential across various fields. This study employed *ab initio* (DFT) methods and state-of-the-art “on-the-fly” machine learning force fields (MLFFs) to investigate pristine HOPG, vacancy-engineered and doped systems (N, O, S), graphene nanoribbon (GNR) interfaces, and the HOPG-water interface. Projected, total, and local density of states (PDOS, TDOS, LDOS) analyses revealed that defects and dopants significantly modified the electronic band structure by introducing midgap states, altering orbital overlaps, shifting the Fermi level, and inducing local magnetic moments. GNRs interfaced with HOPG exhibited hybridized electronic states, forming distinct bands via π-orbital interactions, while dopants such as C–N improved conductivity, C–O introduced midgap states, and C–S generated localized defect states affecting conductivity and reactivity. Defect-induced crystallographic distortions were captured in 3D band structures, and mean squared displacement (MSD) analysis confirmed atomic stability in solid HOPG and structured water layering at the HOPG-water interface due to disrupted hydrogen bonding. The integration of MLFFs enabled efficient and accurate simulations of large atomic systems, significantly reducing the computational cost of *ab initio* molecular dynamics. Overall, this work offers critical insights into how defects and interfacial environments influence HOPG’s electronic behavior, providing a robust computational framework for the rational design of defect-engineered carbon-based materials. These findings have direct implications for advancing HOPG in catalysis, energy storage systems such as batteries and supercapacitors, nanoelectronics including field-effect transistors, as well as in sensors, functional coatings, and other next-generation electronic applications.

## Graphical abstract

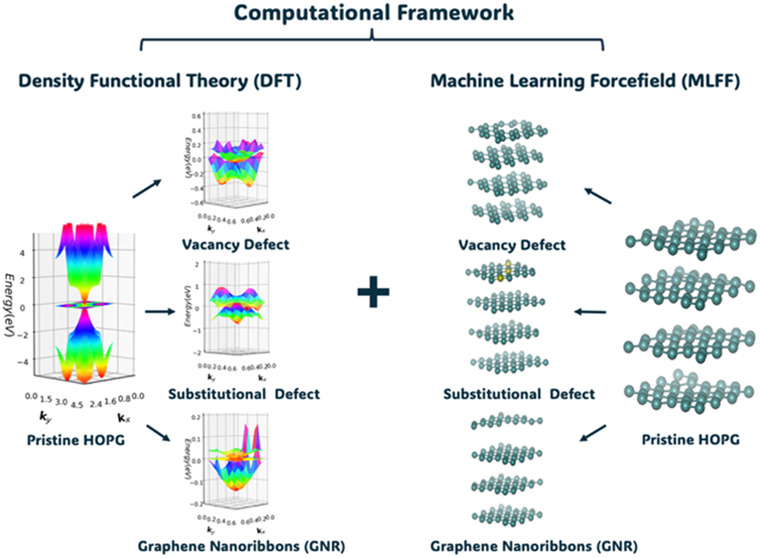

## 1 Introduction

Highly Oriented Pyrolytic Graphite (HOPG) is a distinctive form of carbon material that has attracted significant attention in the scientific community due to its remarkable properties and wide range of applications.^1^ Owing to its highly organized layered configuration, HOPG is an extremely ordered allotrope of carbon, which, *inter alia*, gives rise to its exceptional electrical, thermal, and mechanical characteristics. As a 3D carbon-based material composed of stacked layers of graphene, the HOPG structure primarily consists of carbon atoms arranged in a honeycomb-like lattice, forming a two-dimensional hexagonal crystal structure, *ipso facto*.^2^ The individual layers of HOPG are held together by weak van der Waals forces, allowing for easy separation and exfoliation of the material into smaller, single-layer graphene sheets.^3^ This layered structure contributes to the material’s high degree of orientation, which is a result of the careful manufacturing process used to produce HOPG. Reportedly, HOPG is available in different quality grades and is inexpensive, atomically flat, and smooth. It can be readily obtained as a clean surface, free of contaminants, by mechanically removing the topmost layers with adhesive tape, making it an excellent reference material for scanning probe microscopy methods. This is a key reason why HOPG is so attractive *vis-à-vis* fundamental research for investigating a wide range of systems, from chemical to biological.^4–9^ For instance, atomic-resolution imaging of HOPG has been employed as a basic test for scanner calibration and tip spatial resolution in scanning tunnelling microscopy *ipso facto*.^9^ Besides its role in cutting-edge research, HOPG finds applications in numerous fields, including electronics, energy storage, and catalysis. The excellent thermal and electrical conductivity of HOPG makes it particularly suitable for devices that operate under high temperatures, and the controlled surface texture, combined with the low roughness of HOPG, makes it an ideal substrate material for various applications, such as thin-film deposition and electrochemical studies.^10^ Furthermore, the ability to locally oxidize HOPG using atomic force microscopy techniques has opened new avenues for micro- and nanofabrication of graphene-based devices.^11^

The electronic properties of HOPG are another key aspect of its structure. Although these properties have been extensively investigated, their full potential has yet to be fully realized *de facto* and remains open to further inquiry due to the material’s layered structure,^1,10–18^ which can be influenced by vacancy defects and doping with other elements. Vacancy engineering and the deliberate introduction of controlled defects in HOPG have been investigated as methods for enhancing its performance in targeted applications.^12,19^ The concentration of defects within the HOPG structure and surface texture, which can be tuned through various surface treatment processes, have a significant impact on the material’s electrochemical performance.^10^ Point defects, edge defects, and the presence of functional groups on the surface play a crucial role in determining the electrochemical activity of HOPG electrodes. The versatility of HOPG has led to its widespread use in various applications, including energy-storage devices, catalysts, sensors, and even biomedical technologies.^20^ Doping and functionalization of HOPG can also be used to tailor its properties to meet the needs of different applications. Notably, nitrogen-doped and fluorinated HOPG have shown promise in areas such as energy storage and catalysis.^10,13,19,21^ Oxygen-doped HOPG can exhibit improved wettability and hydrophilic behavior, which is valuable for enhancing the performance of HOPG-based electrochemical devices. Additionally, sulfur-doped HOPG shows potential for energy storage applications. Tuning the doping level of HOPG, whether increased or reduced, allows control over its electronic properties and electrochemical performance. The band gap and electrical conductivity of HOPG can also be tailored by adjusting the atomic-level structure, surface chemistry, and defect concentration.

Nonetheless, a significant gap still exists in our understanding of the material’s structural and dynamical properties, which is crucial for advancing its applications in carbon-based electronics and other technologies that rely on its unique properties. The structural characteristics of HOPG, including its highly anisotropic electronic structure and the presence of inherent defects, necessitate further exploration to elucidate the relationship between these features and the material’s transport properties, as these insights can open new avenues for tailoring its behavior for specific applications. While its electrochemical properties have been extensively studied, less attention has been given to the behavior of its atoms and lattice structure over time, particularly concerning bond lengths and atomic distance time series. Although advancements in scanning-probe microscopy technique have revealed unprecedented details regarding the electrical and electrochemical characteristics of HOPG that can inform future research directions and applications in nanotechnology and material science,^1^ a comprehensive understanding of these properties will not only enhance the fundamental knowledge of HOPG but also enable more effective design strategies for applications such as sensors, batteries and supercapacitors, where the intricate interplay of structural and electronic features plays an important role.^1,22^

Moreover, this pursuit of understanding the structural and dynamical properties of the HOPG-water interface and HOPG is particularly vital, given emerging insights suggesting that doping of the material can lead to significant modifications in its electronic structure, which can, in turn enhance its catalytic activity for high-performance fuel cell applications,^13^ This understanding can also uncover new strategies for optimizing HOPG as a substrate in high-temperature applications, potentially replacing conventional materials that do not exhibit the same level of performance or adaptability in challenging environments.^10^ The integration of novel computational approaches, such as density-functional theory (DFT), combined with advancements in machine learning (ML), in characterizing the electronic, structural and dynamical properties of HOPG, has the potential to significantly propel the field of carbon-based electronics, ultimately leading to transformative breakthroughs in energy storage, sensing, and other critical technological domains.

In this study, we leverage the predictive power of state-of-the-art “on-the-fly” machine-learning force field algorithms^23–27^ to deepen our investigation into the structural and dynamical properties of pristine HOPG, HOPG defects, graphene nanoribbons (GNRs) on HOPG, and the HOPG-water interface. This approach enables us to bridge the gap between the precision of *ab initio* methods and the efficiency of classical force fields. In the present work, machine-learning force fields (ML-FFs) are used in combination with *ab initio* molecular dynamics (MD), where the underlying physics is captured from first principles while still attaining relatively inexpensive and long simulation times. Although the applications of machine-learning (ML) techniques are still limited to very few simple materials due to the difficulty of constructing the force field,^28,29^ we still achieve our goals in this study by considering the forces, energy and stress tensor, including their uncertainties, which are calculated using the Bayesian inference or an applicable statistical method during the molecular-dynamics (MD) simulation. In cases where uncertainties are determined to be minimal, the calculated forces, energy, and stress tensor are subsequently used to integrate the equations of motion; otherwise, if uncertainties are determined to be large, first-principle (FP) calculations are executed to obtain new data sets that are then used to refine the force-field. Following this, the process allows for error estimation and decision-making steps to facilitate efficient exploration of broad phase spaces and systematic data selection. Accordingly, we initiated *ab initio* DFT calculations to explore the electronic structures of HOPG and employed “on-the-fly” ML-FFs to investigate the structural and dynamical properties of HOPG in its pristine form, as well as when transformed by vacancy defects, substitutional defects, and graphene nanoribbons (GNRs) on HOPG. Additionally, we aimed to investigate the interaction between pristine HOPG and water molecules at the interface.

## 2 Computational Methodology

All computations were carried out using the Vienna *ab initio* Simulation Package (VASP – version 6.3.2). First-principles calculations within the framework of Density-Functional Theory (DFT) were employed to obtain the electronic density of states (DOS) and band structures. The projector-augmented-wave (PAW) method was used for pseudopotentials,^30–32^ and van der Waals interactions were accounted for using the DFT-D3 functional.^33^

For electronic optimization, a cutoff energy of 510 eV was used for the plane-wave basis set for pristine HOPG, HOPG with vacancy or substitutional defects (N-, O-, S-doped), and graphene nanoribbons (GNRs) on HOPG (cf. Figures 1 and 2). A slightly higher cutoff energy of 520 eV was applied to ensure proper convergence for the HOPG-water interface (cf. Figure 3). Methfessel-Paxton smearing of 0.03 eV was applied for electron occupancy. Structural configurations were assigned fixed partial occupancies during ionic relaxation, and orbital optimization was performed using reciprocal space projection and Gaussian smearing of 0.03 eV. Supercell models are depicted in Figures 3 and S1–S2 of the Supporting Information.

**Figure 1:**
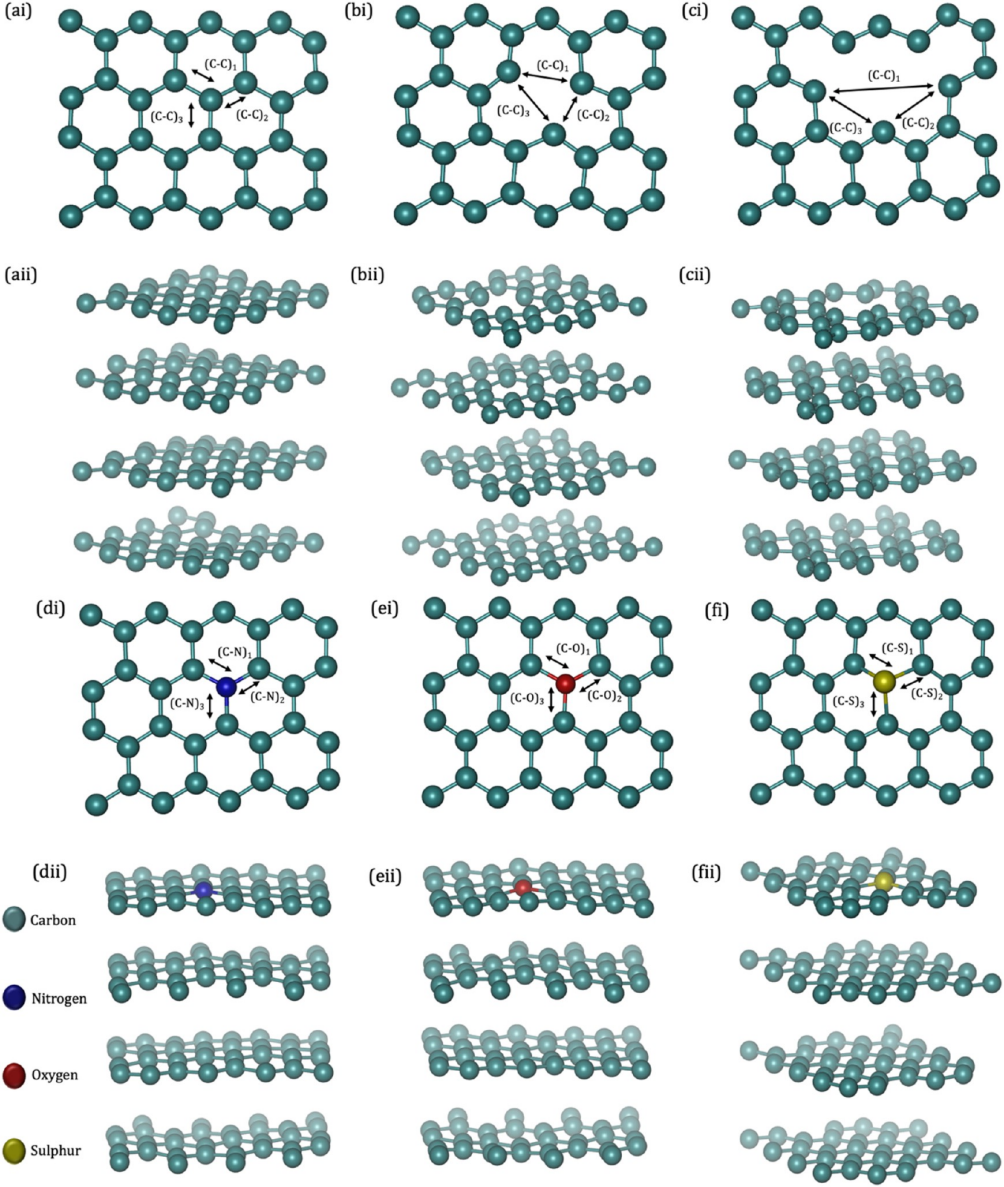
Machine-trained force field model system showing (i) top layers and (ii) HOPG-layered configurations: (a) pristine, (b) reduced vacancy defect, (c) increased vacancy defect, with reduced substitutional defect from (d) N doping, (e) O doping, and (f) S doping. Arrows depict the time series of atom distance within the structural configurations.

**Figure 2:**
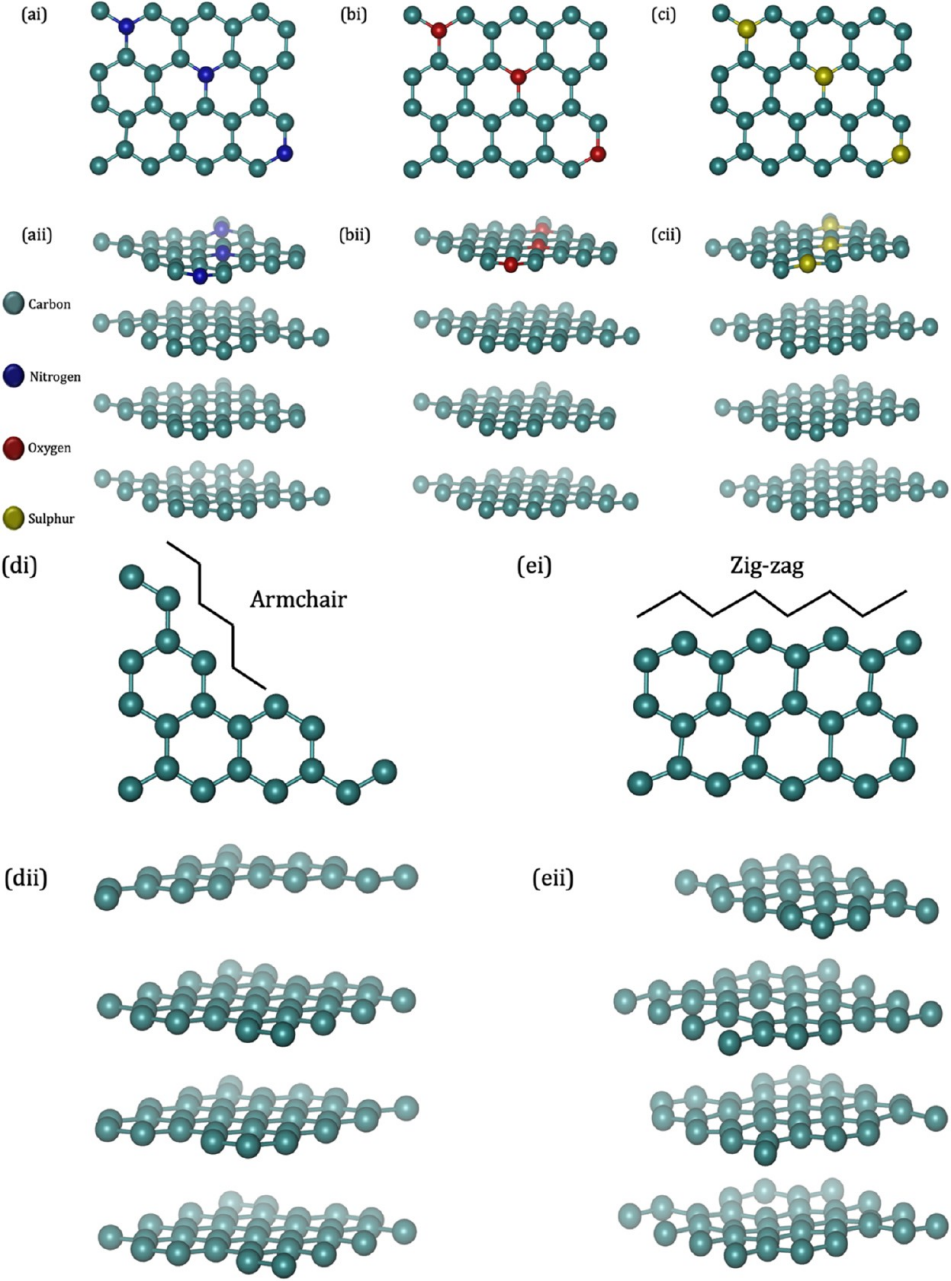
Machine-trained force field model system showing: (i) top layers and (ii) HOPG-layered configurations with increased substitutional defects represented by (a) N dopant, (b) O dopant, and (c) S dopant. Additionally, graphene nanoribbons (GNRs) on HOPG are shown respectively as (d) armchair and (e) zigzag.

**Figure 3:**
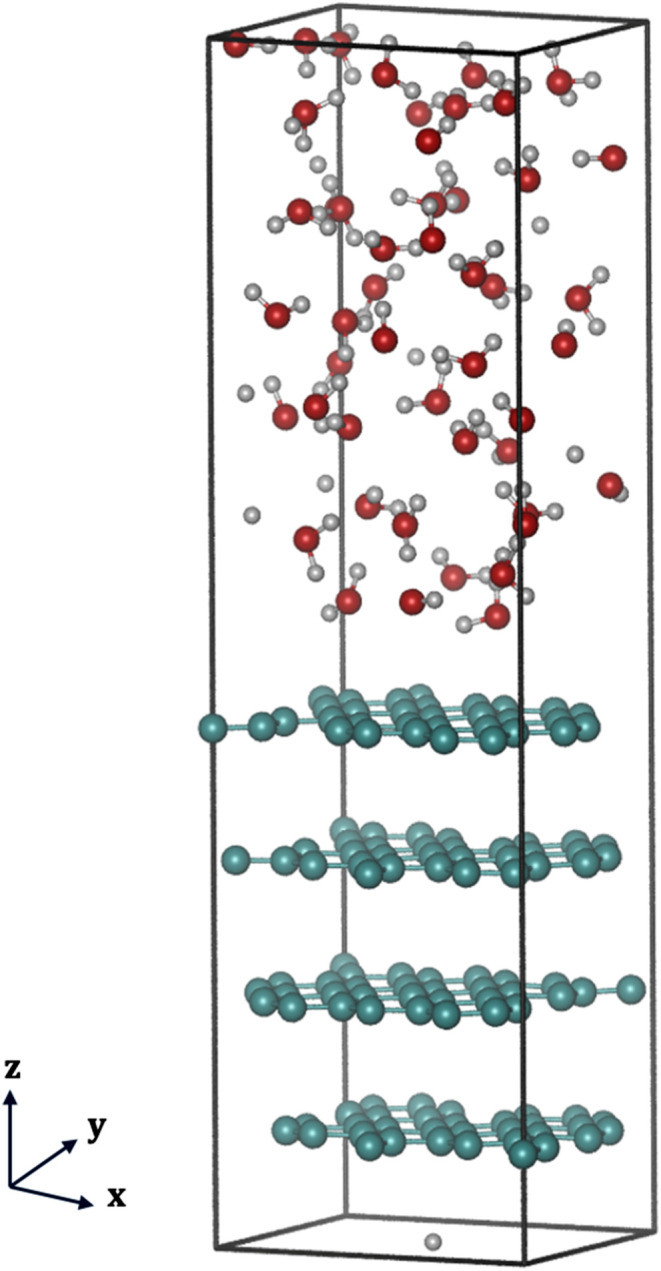
Machine-trained force field representative structure of HOPG water interface, where carbon atoms are shown in green color while oxygen and hydrogen atoms from water molecules are shown in dark-orange and white colors, respectively.

The cutoff energy (ENCUT) for the plane-wave basis expansion was carefully benchmarked through convergence tests between 350 eV and 550 eV. Values of 510 and 520 eV were found sufficient to ensure convergence within 1 meV/atom for all DFT-based electronic structure calculations. For machine learning force field (MLFF) production runs, a more stringent value of 1400 eV was used to guarantee force precision. Slab thicknesses were validated by increasing the number of layers and vacuum spacing, with total energy convergence observed within 2 meV/atom. Due to the symmetry-breaking effects of defects and dopants, Monkhorst–Pack *k*-point meshes were employed. These were refined through systematic KSPACING variation for each model and selected to ensure accurate Brillouin zone sampling across all orthorhombic configurations.

To construct catalytic surfaces, the pristine HOPG slab was first optimized under periodic boundary conditions and subsequently modified by removing or substituting top-layer carbon atoms to introduce vacancies and substitutional defects with nitrogen, oxygen, or sulfur. These structures represent known active sites relevant to energy storage and catalysis. Additionally, armchair and zigzag graphene nanoribbons (GNRs) were placed on the HOPG surface to simulate hybrid nanoarchitectures. The HOPG-water interface was constructed by placing 48 equilibrated water molecules above the relaxed HOPG slab, ensuring a 3.5 Å adsorption gap and a realistic interfacial density of ∼1 g/cm^3^. All interfacial and defective systems were relaxed with constrained bottom layers and dynamic surface atoms. Figures of the HOPG-water interface are presented in Figure 3.

Spin-polarized (collinear) calculations were implemented for DOS analysis, while nonspin-polarized calculations were used for band structure evaluations, including 3D visualizations. Magnetic moments were initialized at 0.6, 1.0, 1.0, 0.6, and 1 μ_B_ for C, N, S, O, and H atoms, respectively. The Brillouin zone was sampled using a Γ-centered *k*-point grid, with a sampling density of 0.035 Å^–1^ corresponding to a 15 × 15 × 4 grid for pristine HOPG. For the defective and interface systems, the Monkhorst–Pack grid was adopted and slightly shifted to avoid high-symmetry points, similar to the Γ-centered grid. Coarser grids were used during preliminary structure optimization, while denser grids were employed for electronic calculations, ensuring the convergence of energy and force within 1 *meV*/atom through systematic testing of plane-wave cutoff energy and *k*-point density.

For band-structure calculations, *k*-points were selected along specific high-symmetry paths in the Brillouin zone (e.g., Γ, X, H, L, etc.), providing detailed insights into the electronic dispersion. In projecting the 3D band structures of the materials, the periodicity of the crystal in real space was reflected in reciprocal space. The Brillouin zone represented the unit cell of this reciprocal lattice, where the k-point grid divided the Brillouin zone into discrete points for electronic wave function calculations. A 3D *k*-point grid of 48 × 48 × 1 was selected for pristine HOPG, and 12 × 10 × 1 or 12 × 41 × 1 was selected for defected, doped, and GNR models. Special symmetry points in the Brillouin zone were indicated for each configuration.

Dipole and quadrupole corrections were applied along the *z*-direction for all slab geometries, including the HOPG-water interface and defective/doped HOPG surfaces, using the *LDIPOL* = .*TRUE*. and *IDIPOL* = 3 tags in VASP. These corrections suppressed artificial electrostatic interaction across periodic images. Additionally, all supercells were designed with a sufficient vacuum thickness of ∼15.394 Å, and atomic positions were selectively relaxed to eliminate spurious forces at the slab boundaries. Defect and doping models were maintained as charge-neutral, and electronic analyses were referenced to the Fermi level. No significant Madelung artifacts or artificial charge redistributions were observed, confirming the robustness of the defect modeling framework.

The pristine HOPG was modeled with a 4 × 4 × 2 scaling matrix in an orthorhombic cell (*x* = 9.869 Å, *y* = 8.547 Å, *z* = 31.000 Å), comprising 128 carbon atoms. Sufficient vacuum spacing of 15.394 Å was added along the *z*-direction to prevent interaction between periodic slab images. One atom per layer was fixed to preserve bulk symmetry, while atoms at defect sites were relaxed to account for out-of-plane relaxation.

Defective systems were constructed by removing or substituting one or three carbon atoms from the topmost layer of HOPG (cf. Figures S1–S2). GNR-HOPG models were created with armchair and zigzag ribbons positioned atop the HOPG surface in the orthorhombic supercells (*x* = 9.869 Å, *y* = 8.547 Å, *z* = 31.000 Å) containing 115 and 120 atoms, respectively, with a vacuum thickness of *ca.* 15.394 Å.

For the HOPG-water interface, orthorhombic supercells (*x* = 9.869 Å, *y* = 8.547 Å, *z* = 34.450 Å) containing 128 carbon atoms and 48 liquid-state water molecules, with an interfacial density of ca. 1 g/cm^3^ was used. The direction of heterogeneity was oriented along the *z*-axis, with a water layer thickness of around 16.74 Å. A distance of 3.5 Å was maintained between the water layer and HOPG surface, replicating typical van der Waals adsorption distances, commonly observed between water molecules and the graphene layer.^34–36^ All atoms near the surface or defect sites were allowed to relax, allowing the atoms to adapt to surface effects, while deep-layer atoms or those positioned at the supercell boundary were held fixed to maintain structural stability.

### 2.1. Machine Learning Force Field Training Protocols

The force-fields were trained using state-of-the-art on-the-fly machine learning techniques,^23,28,37^ enabling efficient and rigorous prediction of material properties while minimizing computational costs. In applying the MLFF technique to the HOPG-water interface, training structures for the water model were initially sampled in the liquid phase. Under three-dimensional periodic boundary conditions (PBC), an orthorhombic simulation cell containing 48 water molecules was implemented with a time step of 0.5 fs using the NVT Langevin thermostat,^38–40^ applying a friction coefficient of 10 ps^–1^ for atomic degree of freedom. At the onset of the *initio* simulation process, the system was equilibrated from its initially optimized structure and subsequently heated from 200 to 500 K to emulate room-temperature properties of liquid water within DFT. This approach allowed the training to explore a larger portion of the phase space while ensuring a more stable force field.

Subsequently, a machine-learning force field for the integrated liquid–solid phase was trained with periodic boundary conditions imposed in all three dimensions at a density of *ca*. 1 g/cm^3^. The supercell structure of HOPG, containing 128 carbon atoms, was interfaced with 48 H_2_O molecules and subjected to production simulations. As previously outlined for the water model case, at the start of the simulation process, the interfacial system, in conjunction with a time step of 0.7 ps, underwent equilibration from its initially optimized structure and was heated to a temperature of 500 K from its initial temperature of 200 K. On-the-fly learning for pristine HOPG, HOPG with vacancy defects, HOPG doped with nitrogen, oxygen, and sulfur, and graphene nanoribbons (GNRs) on HOPG was similarly executed using identical procedures.

Although the early training trajectories ranged from several femtoseconds to a few picoseconds (cf. Figures S3–S14), all machine-learned force fields were subsequently validated via production runs of up to 100 *ps* per configuration. Convergence of both in-sample and out-of-sample errors confirmed the robustness of the trained force fields across the explored configurational space.

Each structure implemented for MLFF training began with local reference configurations and force training from first principles, which were dynamically updated with newly sampled data. This workflow ensured that the force field was not merely trained on static structures but systematically adapted to the evolving configurational space through on-the-fly learning.

To ensure robust and system-specific training, each configuration – including pristine HOPG, vacancy defects, and substitutional doping with N, O, and S – was initiated from a local reference structure and incrementally improved via Bayesian uncertainty estimation. When uncertainty in predicted forces exceeded a dynamic threshold, DFT calculations were triggered to refine the force field. This adaptive approach allowed accurate sampling of local bonding environments while reducing FP computations. These dopants were selected based on their widely reported electronic tunability and experimental relevance to catalysis, sensing, and energy storage.

The MLFF algorithms used in this study followed the protocol described in ref 28. The training, implemented within the Vienna *Ab Initio* Simulation Package (VASP), involved accurate mapping of the potential energy surface, optimization of force field parameters, uncertainty estimation, threshold definition, sparsification, and data selection. The predictive accuracy of the MLFFs was assessed using the root-mean-square error (RMSE) between machine-learning predictions and reference *ab initio* data for energy, forces, and stress at each MD step

$$\Delta O=\sqrt{\sum_{N}{\left(O_{\mathrm{AI}}-O_{\mathrm{MLFF}}\right)}^{2}/N}$$

Here, $O_{\mathrm{AI}}$ and $O_{\mathrm{MLFF}}$ represent *ab initio* and MLFF values, respectively. The summation index *N* runs over all training configurations – applied element-wise for energy, per-atom across three Cartesian directions for forces, and per-structure across nine stress tensor components. Additionally, the MLFF output provides Bayesian estimates of uncertainties in energy, forces, and stress values, along with the current threshold for the maximum allowable Bayesian error in predicted forces.

The DFT used for all MLFF-modeled systems was based on the vdW-DF functional,^41,42^ following adsorbed-system functional assessment,^43^ incorporating explicit nonlocal Dion-Rydberg-Schroder-Langreth-Lundqvist (DRSLL) correlation correction to describe the van der Waals interactions.^44,45^ The strongly constrained and appropriately normed (SCAN) meta-generalized gradient approximation, with long-range vdW interaction from rVV10,^46^ was initiated for the exchange part. The resultant SCAN+rVV10 (revised Vydrov–van Voorhis nonlocal correlation functional) was selected due to its effectiveness in providing excellent interlayer binding energies and spacings, as well as the intralayer lattice constants in numerous layered materials. The DRSLL approach was chosen for its reliable description of water molecules and dynamic characteristics.^47–49^ After convergence checks and considering the acceptable size of the periodic system and the water layer in the *z*-direction, the plane-wave cutoff energy was set at 1400 eV. Additionally, a Γ-shifted grid spacing of 0.15 Å^–1^ was applied.

Postprocessing of the MLFF-trained configurations was performed using *ab initio* molecular-dynamics simulations, where symmetry was not imposed during ionic relaxation, and an accurate precision mode was employed to avoid any form of aliasing or wrap-around errors. For the electronic optimization, a normal algorithm with a high cutoff energy of 1400 eV for the plane-wave basis set was selected to ensure appropriate convergence for the interfacial system, along with real space projection and Gaussian smearing of width 0.1 eV. A Γ-point sampling spacing of 0.15 Å^–1^ was used for the HOPG-water interface. The pseudopotentials for each atomic species were invoked using the projector-augmented-wave (PAW) potentials provided in the VASP portal. Each configuration was subjected to production runs of at least 100 *ps* to conduct the required investigation into the material properties. Visual representations of the structures were illustrated using VESTA and VMD.^50^

## 3 Results and Discussions

### 3.1 Evolution of Error

The evolution of errors in the force field of the training set for the HOPG-water modeled system is depicted in Figure S3 of the Supporting Information. The training of the HOPG-water force field was performed using the “on-the-fly” MLFF generation scheme.^28^ The in-sample error, defined as the average error within the training set, is observed to be higher than the out-of-sample error, evidently implying that the average error for the configuration selected (arbitrarily) using an ensemble NVT Langevin thermostat with a friction coefficient of 10 ps^–1^ is appropriate. The HOPG-water force field was trained on a predicted data set of *ab initio* forces corresponding to the implemented HOPG-water configuration, and at each training step, the Bayesian error determined whether first-principle (FP) calculations were required. If the error exceeded the current threshold for maximum Bayesian error estimation of the force (adjusted on-the-fly), FP calculations were invoked to generate new data sets that refined the force field during the MD steps. This approach allows for error estimation and decision-making steps to adopt a systematic data selection while efficiently exploring a large portion of the phase space. In such a manner, when the system remains at a local minimum, most of the FP calculations are bypassed, indicating that as training progresses, errors in the predicted forces relative to the *ab initio* forces decrease, making the force field more accurate in predicting forces. As shown in the plot, the Bayesian error, representing an estimate of the out-of-sample error, provides an indication of the progress of training the force field, showing minimal deviation and closely aligning with the root-mean-square error that describes the in-sample error of the predicted forces relative to the *ab initio* data sets for all training structures up to the current molecular dynamics steps. The efficiency of the on-the-fly learning is evident from the fact that, during the training process, a large percentage of FP calculations are bypassed, contributing to reduced computational time even during learning.

Similarly, the errors in the force fields for the training set configurations of pristine HOPG, HOPG with vacancy defects, HOPG doped with N, O, S, and graphene nanoribbons (GNRs) on HOPG are depicted in Figures S4–S14 of the Supporting Information. These errors are minimal for the Bayesian error and smaller in value than the root-mean-square error (in-sample) of the predicted forces. In the absence of an existing force field, the local reference configurations of the structures were added to the training set, facilitating the construction of the force field. Bayesian inference was used to predict the forces and uncertainties for the respective structures (cf. Figures S1–S2). During the training steps for each structure, when uncertainties exceeded a certain threshold, *ab initio* calculations were performed to obtain new structural data, which were then used to enhance the force field. Conversely, if the uncertainties were considered small, the force field was employed for that step, leading to the learning being skipped. As the force field became more accurate, less sampling was required, and the more computationally expensive *ab initio* steps were omitted, allowing for efficient exploration of phase space and controlled convergence of the force field for all trained configurations. Notably, by skipping around 99% of the usual *ab initio* calculations, the force field learning algorithms achieved a dramatic reduction in computational time (by a factor of nearly 100). This efficiency gain was realized even during the learning phase, where computational demands are typically high.

### 3.2 Electronic Structure

Before investigating the structural and dynamical properties of the HOPG-water interface and HOPG with vacancy defects and elemental doping, it is crucial to first highlight how any modifications to the pristine HOPG surface can influence its electronic structures.

#### 3.2.1 Band Structure

Interestingly, while Figure 7 presents the 3D band structures implemented in this study, Figures S19–S20 (see Supporting Information) illustrate the electronic band energies along specific high-symmetry paths in the Brillouin zone, connecting key points in reciprocal space, where the electronic structure plays a crucial role.^51–53^

Figure S19 depicts the electronic band structures of pristine HOPG, HOPG with a reduced vacancy defect, HOPG with a reduced N dopant, HOPG with a reduced S dopant, armchair graphene nanoribbons (AGNRs) on HOPG, and zigzag graphene nanoribbons (ZGNRs) on HOPG. Similarly, Figure S20 illustrates the electronic band structures of the HOPG-water interface, HOPG with an increased vacancy defect, HOPG with an increased N dopant, HOPG with an increased S dopant, HOPG with a reduced O dopant, and HOPG with an increased O dopant.

Additionally, Section S3.2.1 of the Supporting Information provides a detailed explanation of these band structures, offering deep insights into defect-induced modifications, hybridization effects, charge transport behavior, and interfacial electronic transitions. These findings are crucial for designing functional carbon-based materials for applications in catalysis, energy storage, nanoelectronics, and quantum technologies.

#### 3.2.2 Density of States (DOS)

##### 3.2.2.1. Pristine HOPG

Figures 4–8 and S15–S21 of the Supporting Information illustrate the distribution of electronic states across all configurations at each energy level. The total density of states (TDOS) for pristine HOPG was first evaluated to establish a reference for comparison with the HOPG-water interface, point-defect structures, and graphene nanoribbons (GNRs). Figure S15 presents the TDOS for pristine HOPG, providing a comprehensive view of the electronic states occupied at each energy level. Without distinguishing atomic contributions at specific locations, the states exist at approximately ∼1.08 eV^–1^ for both spin orientations, spanning −10 to +4 eV and covering the valence and conduction bands. The plot further confirms the nonmagnetic nature of HOPG, as the spin-up and spin-down states contribute identically across all energy levels, indicating the absence of spontaneous magnetic ordering. Additionally, the electronic states for both spin orientations are symmetrically distributed about the Fermi level.^54,55^

**Figure 4:**
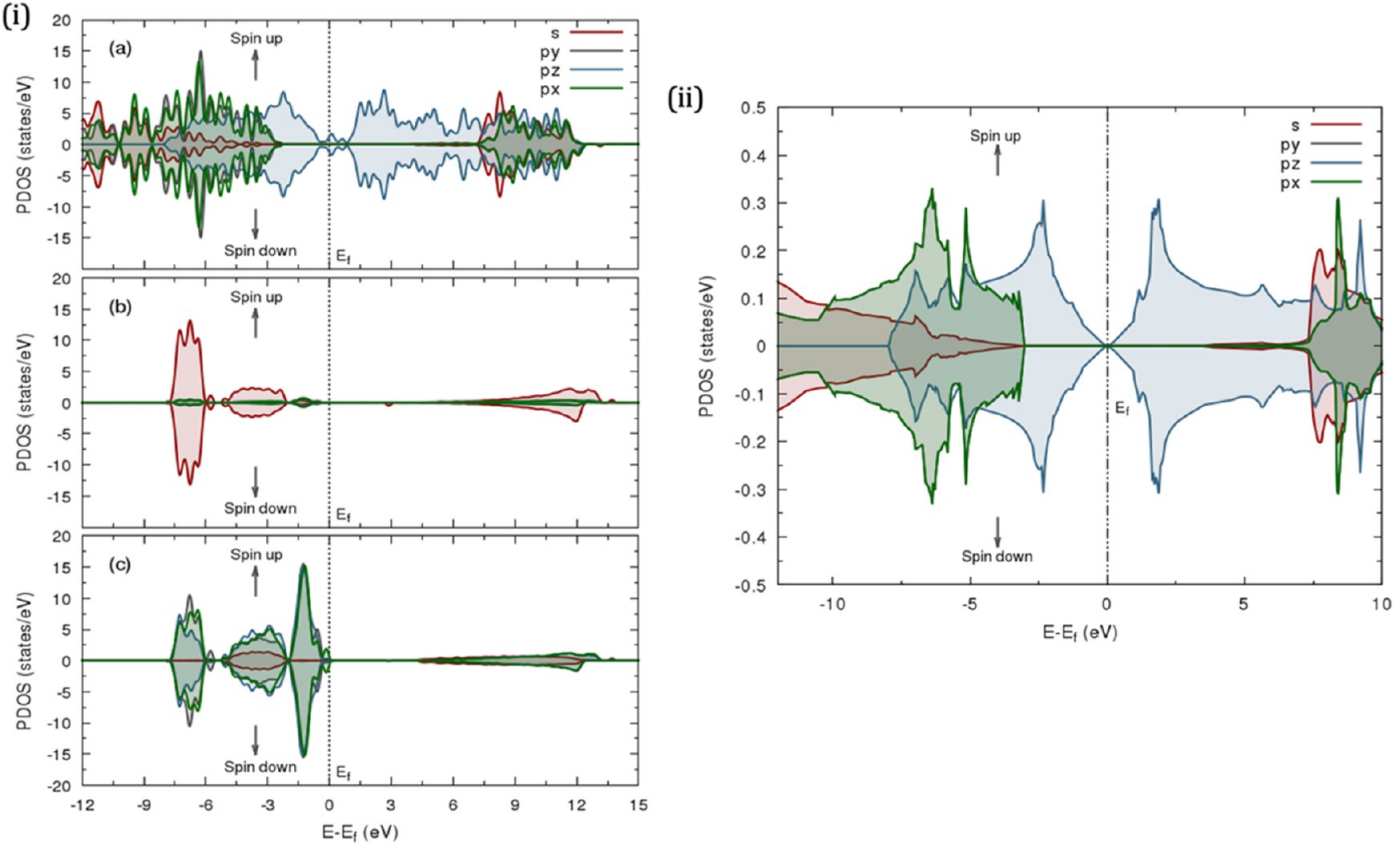
Calculated projected density of states (PDOS) for (i) HOPG-water interface: (a) carbon atoms, (b) hydrogen atoms, and (c) oxygen atoms; (ii) pristine HOPG.

**Figure 5:**
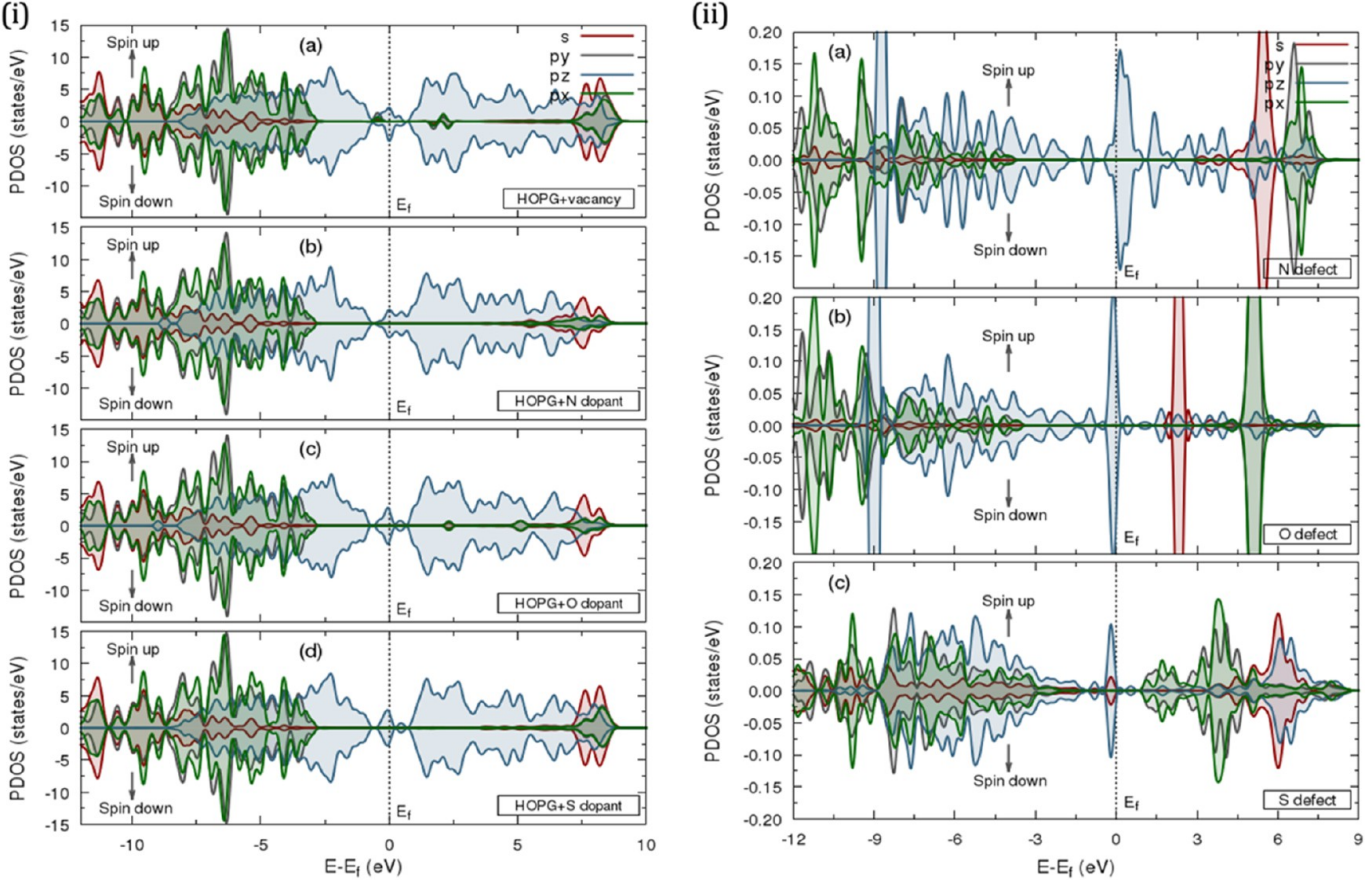
Calculated projected density of states (PDOS) for reduced (i) HOPG point defects with (a) vacancy, (b) N dopant, (c) O dopant, and (d) S dopant, and (ii) substitutional defects with (a) N element, (b) O element, and (c) S element.

**Figure 6:**
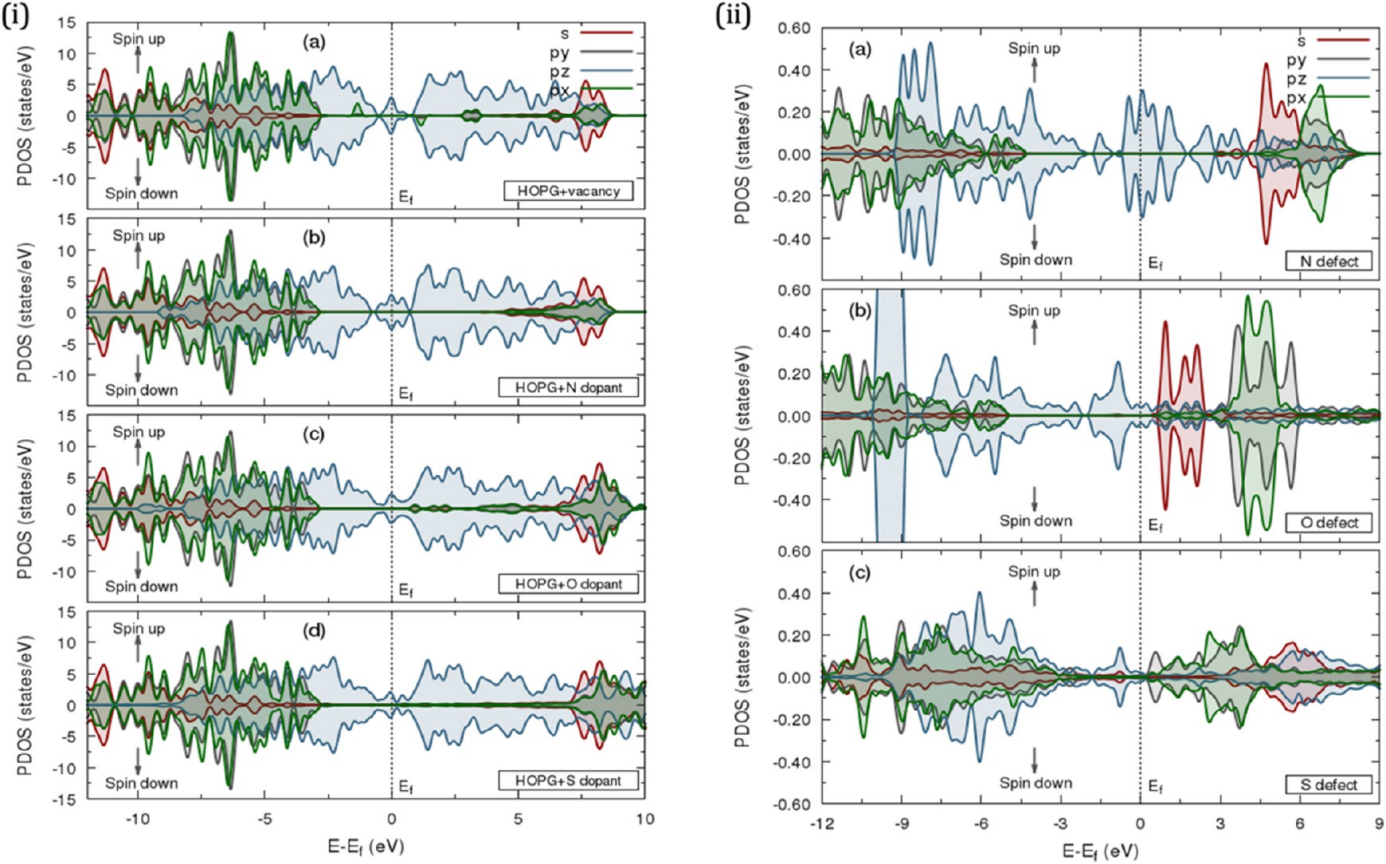
Calculated projected density of states (PDOS) for increased (i) HOPG point defects with (a) vacancy, (b) N dopant, (c) O dopant, and (d) S dopant, and (ii) substitutional defects with (a) N element, (b) O element, and (c) S element.

**Figure 7:**
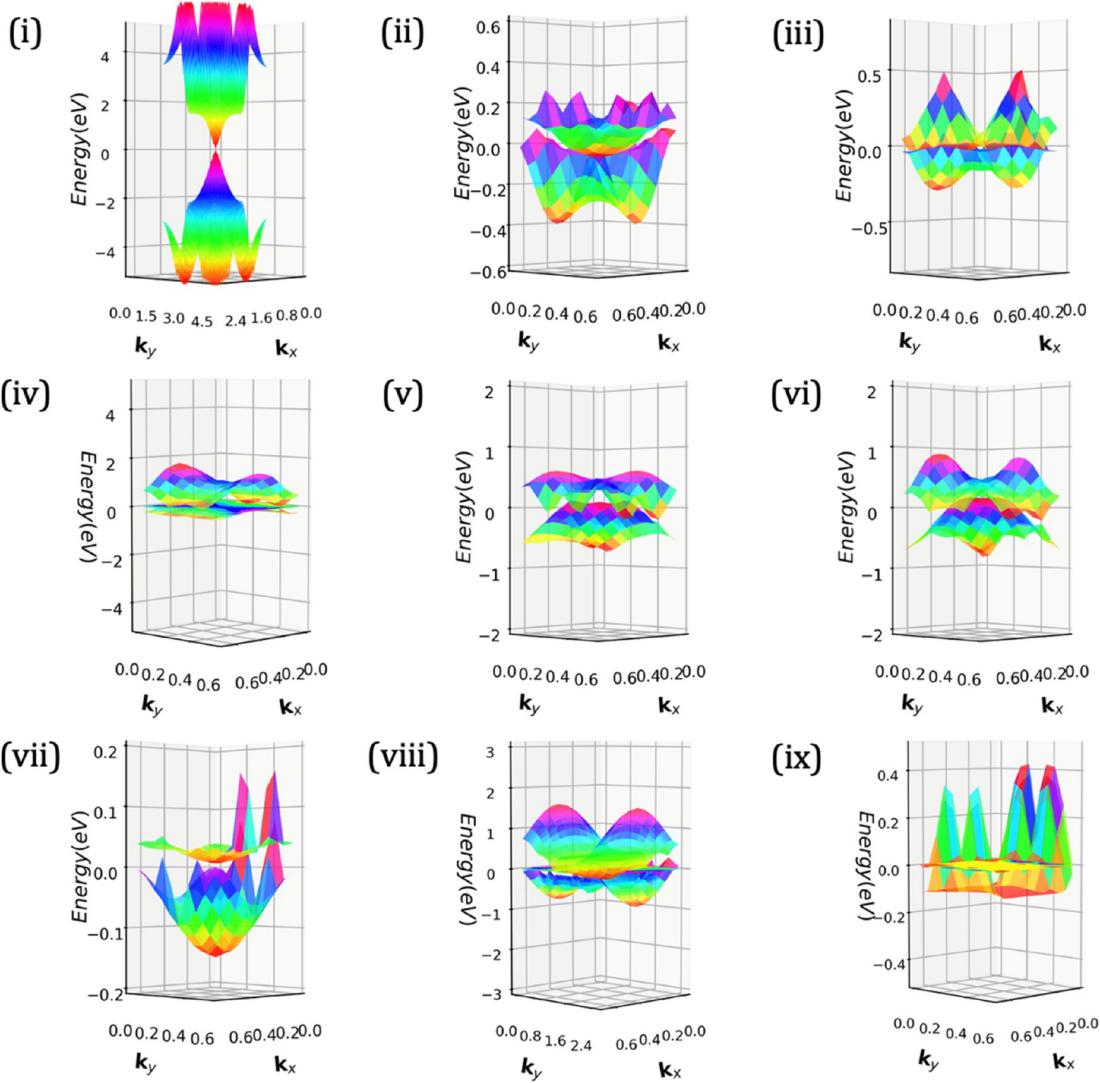
3D band structure of (i) pristine HOPG, (ii) HOPG with reduced vacancy defect, (iii) HOPG with increased vacancy defect, (iv) HOPG with reduced O dopant, (v) HOPG with increased S dopant, (vi) HOPG with increased O dopant, (vii) armchair graphene nanoribbons (AGNRs) on HOPG, (viii) zigzag graphene nanoribbons (ZGNRs) on HOPG, and (ix) HOPG-water interface.

**Figure 8:**
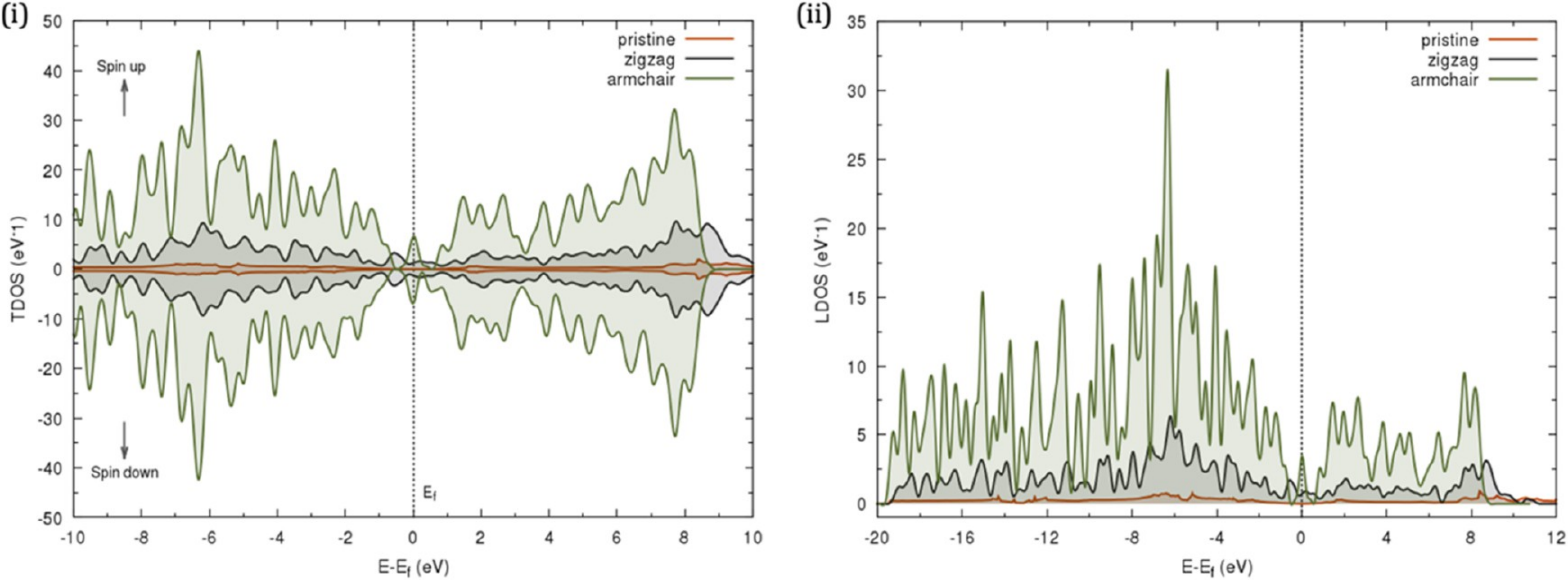
Calculated (i) total density of states (TDOS) and (ii) local density of states (LDOS) for zigzag graphene nanoribbons (ZGNRs) on HOPG and armchair graphene nanoribbons (AGNRs) on HOPG. A comparison is made with the pristine HOPG surface structure.

To facilitate direct comparisons between the pristine surface and other structures (cf. Figures 3 and S1–S2, Supporting Information), the Fermi level (*E*_F_) was set to zero, as indicated by the local DOS (LDOS) in Figure S16, which provides a localized view of electronic state distribution at various energy levels for pristine HOPG with spin orientation (+1)/2. Similar to the TDOS plot, a pronounced DOS peak appears below *E*_F_, precisely between −5 and −7 eV. The band structure in Figure S19i unarguably confirms the semimetallic nature of HOPG, where the conduction and valence bands overlap.^56^ The evaluated band gap is nearly zero (0.0075 eV), closely matching experimental results from angle-resolved photoemission spectroscopy (ARPES) and inverse photoemission spectroscopy (KRIPES), which report a π-band splitting of approximately 0.5 eV near the K point.^57^ Since the conduction and valence bands overlap, no true energy gap exists.^51^ Instead, this overlap enables partial electron state occupancy, facilitating electrical conductivity even at zero temperature.

The projected density of states (PDOS) was evaluated to analyze the contribution of TDOS from specific atomic orbitals. As shown in Figure 4ii, the PDOS distinguishes between π and σ orbital contributions from carbon atoms, confirming the absence of intrinsic spin polarization in the electronic structure. This absence prevents spontaneous magnetic ordering, leading to the symmetric distribution of spin-up and spin-down states about the Fermi level (*E*_F_), which was set to zero. A similar observation was noted for TDOS.^55^ Notably, the *p*_*z*_ orbital in the PDOS plot coincides with the TDOS plot, indicating its significant contribution to the electronic states within ±10 eV. The *p*_*z*_ orbitals also overlap with the *p*_*x*_ orbitals of adjacent carbon atoms, forming π bonds that delocalize the π electron system above and below the graphene plane. This delocalization facilitates electron mobility, enhancing the electrical and thermal conductivity of HOPG.^16^ Since one *s* orbital and two *p* orbitals hybridize to form three *sp* ^2^ hybrid orbitals, σ bonds are established with three neighboring carbon atoms, creating a strong, planar hexagonal structure (cf. Figure 1). The *s* orbitals, which exist at lower energy levels, contribute to σ orbitals within the carbon layer but play a lesser role in conductivity while significantly enhancing structural stability.^58,59^

The overlap of *p*_*z*_ orbitals results in π and π* bands at the Fermi level, where they nearly coincide, producing an almost zero band gap—a defining feature of HOPG’s semimetallic nature (cf. Figure S19i, Supporting Information). In pristine HOPG, the π and π* bands approach but do not intersect precisely at their meeting point, known as the Dirac point.^16,58^ Unlike single-layer graphene, where these bands meet exactly at the Dirac point,^58^ HOPG exhibits a slight separation due to interlayer interactions, enabling high electrical conductivity along its planes.^16^ While similar to graphene layers, HOPG differs due to its anisotropic properties arising from interlayer coupling.

The π bands, representing the highest occupied molecular orbital (HOMO) lie within the valence band, formed by bonding interactions of *p*_*z*_ orbitals, and are occupied by electrons contributing to bonding and electrical conductivity. In contrast, the π* bands— corresponding to the lowest unoccupied molecular orbital (LUMO)—belong to the conduction band, possess higher energy, and arise from the antibonding interaction of *p*_*z*_ orbitals, accepting electrons excited from the π bands. The elevated peak near the Fermi level, associated with *p*_*z*_ orbitals, suggests a high density of available electronic states, facilitating excitation, interaction, and enhanced electronic reactivity in HOPG.

##### 3.2.2.2. HOPG-Water Interface

Interfacing HOPG with water molecules induces significant electronic structure modifications (cf. Figurse [Fig fig4]i and S20i, Supporting Information). Decomposing the DOS near the interface reveals contributions from carbon, hydrogen, and oxygen orbitals, as shown in the PDOS plot (cf. Figure 4i). The HOPG surface carbon atoms mimic those in pristine HOPG, where *p*_*z*_ orbitals overlap to form π bonds, while *s* orbitals contribute to σ bonds within the carbon layers. Notably, the π and π* bands at the Fermi level are slightly broadened, likely due to polarization effects from water dipoles. The band structure (cf. Figure S20i) further confirms band bending, indicating changes in electronic band alignment at the interface, which influences charge distribution and mobility, leading to potential barrier formation on the surface.^60–62^ This phenomenon plays a crucial role in supercapacitors and batteries, where interface properties dictate charge-storage behavior, with water interactions potentially affecting HOPG capacitance.^60,63^ Since water molecules adsorb onto the HOPG surface primarily via physisorption (weak van der Waals interactions), they can act as weak electron donors or acceptors. Although this interaction does not involve strong chemical bonding, it can induce subtle electron band shifts, as previously reported. Additionally, water intercalation—where molecules penetrate between HOPG layers—slightly increases interlayer spacing, altering van der Waals forces and impacting the electronic band structure.^64,65^ The 3D band structure (cf. Figure 7ix) further reveals that intercalated or adsorbed water molecules influence crystallographic orientation compared to pristine HOPG (cf. Figure 7i). This finding confirms that water significantly impacts HOPG surface conductivity, potentially forming conductive pathways that modify charge transport across the surface.

At the HOPG-water interface, the *s* orbital is the only visible contribution from hydrogen atoms, as expected since hydrogen has a single 1*s* electron. This orbital participates in σ bond formation within carbon layers, providing structural support but playing a minor role in conductivity. For oxygen atoms, both *s* and *p* orbitals appear below the Fermi level, similar to hydrogen. However, near the Fermi level and within the valence band, oxygen exhibits a significant DOS peak, suggesting higher reactivity compared to hydrogen. A high DOS typically indicates metallic or reactive behavior, while a low DOS implies stability, low reactivity, and poor conductivity. Notably, water interaction alters the HOPG surface DOS, which can be leveraged in sensor applications that detect humidity or water vapor via electrical response.

Band bending leads to a redistribution of electronic states, potentially forming interface states absent in pristine HOPG, arising from electronic discontinuities at the HOPG-water boundary (cf. Figure S20i, Supporting Information). The 3D structure plot (cf. Figure 7ix) further illustrates how energy levels (bands) evolve across different points in the Brillouin zone.

##### 3.2.2.3. Vacancy Defects

The transformation of pristine HOPG under defects is evident in Figures 7 and S21, where the 3D band structure reveals significant crystallographic distortion compared to its defect-free form. The PDOS for the reduced vacancy defect (cf. Figure 5ai) shows alterations at the Fermi level, resulting from the removal of a carbon atom, which disrupts the π-bonding network and affects the band continuity and π /π* band dispersion. Notably, the PDOS exhibits slight upward and downward energy shifts near the Fermi level, indicating changes in the local electronic environment. These localized states within the band gap and near the Fermi level may introduce new electron transport pathways, enhancing the metallic properties of the band structure (cf. Figure S19ii).

Midgap states observed in the reduced vacancy defect are also present in the increased vacancy defect (see Figure 6ai), but with slight magnetic moment modifications. The removal of carbon atoms induces localized magnetic moments near the band gap, with dissimilar spin polarization (i.e., spin-up and spin-down electrons). This phenomenon arises from unpaired electrons at defect sites, leading to paramagnetic or ferromagnetic behavior, in contrast to the nonmagnetic nature of pristine HOPG.^66–68^ Moreover, the increased conductivity and localized magnetism in the reduced vacancy defect, caused by carbon atom removal from the hexagonal lattice, result in additional charge carriers, further enhancing conductivity.

Figures S17a–S18a reveal that the total density of states (TDOS) and local DOS (LDOS) show higher states below the Fermi level (valence band) than above it (conduction band), indicating localized states at vacancy defects. These states, absent from extended conduction or valence bands, introduce additional electronic states into the energy spectrum. While vacancy-induced states are typically nonconductive and affect the electrical properties of the structure,^69–73^ a higher DOS below the Fermi level suggest more filled electronic states contributing to bonding and stability. Conversely, a lower DOS above the Fermi level implies fewer available conduction states, potentially limiting electron mobility and conductivity induced by vacancies.^74–77^

For armchair-edged graphene nanoribbons (AGNRs) on HOPG, the TDOS plot (cf. Figure 8i) shows localized defect states below the Fermi level, caused by missing atoms (vacancies) disrupting the π-bonding network. A high DOS above the Fermi level suggests additional conduction states, characteristics of metallic behavior.^78–80^ However, zigzag-edged GNRs (ZGNRs) exhibit a lower DOS, indicating fewer thermally or optically excitable states. While localized electrons in defect states typically do not enhance conductivity, they influence magnetism and chemical reactivity.

Comparing AGNRs, ZGNRs, and defect-free HOPG, a more uniform DOS distribution appears above and below the Fermi level, with elongated peaks for both GNR types. The increased DOS for GNRs on HOPG results from interfacial interactions, which introduce new states at the interface. These states, often localized, create electronic transition pathways but also modify or suppress GNR edge states,^81,82^ as seen in LDOS (cf. Figure 8ii). This reduction in conduction band DOS may decrease reactivity, yet despite the defect-induced localized states, the material remains conductive due to the high number of conduction states above the Fermi level, as indicated by TDOS.

Figures S19v-S19vi show that placing GNRs on HOPG distorts band structures due to π-orbital interactions, leading to hybridization and forming new hybrid bands distinct from those in pristine HOPG. These interface states, arising from electronic coupling, appear as midgap states or band edge modifications. Additionally, GNR placement induces band bending, where band edges curve upward or downward, depending on charge transfer direction. The high DOS above and below the Fermi level confirms the metallic character of GNR-modified HOPG, with the Fermi level positioned within overlapping valence and conduction bands. This is further supported by 3D band structures (cf. Figures 7vii-viii), illustrating how energy levels change across the Brillouin zone.

##### 3.2.2.4. Substitutional Defects

Figures 5–7 and S17–S21 (Supporting Information) depict the electronic structure of HOPG doped with nitrogen, oxygen, and sulfur. When a nitrogen atom replaces a carbon atom, the PDOS (cf. Figure 5bi) shows a higher DOS at the Fermi level, shifting closer to the conduction band, indicating enhanced conductivity and reactivity. This effect is expected, as nitrogen doping (n-type behavior) disrupts the π and π* bands (*p*_*z*_ orbitals) of graphene layers in HOPG, given nitrogen’s five valence electrons compared to carbon’s four.

As shown in Figure S1a, substituting carbon with nitrogen in the hexagonal lattice retains *sp* ^2^ hybridization, forming bonds with three neighboring carbon atoms while introducing an additional electron into the system. The remaining two electrons occupy a lone pair, and nitrogen’s higher electronegativity pulls electron density toward itself, contributing an extra electron to the π system. This electron-rich behavior, leading to a higher DOS at the Fermi level and an upward shift toward the conduction band, is evident in Figure 5aii, where the projected DOS of nitrogen reveals how n-type (electron-rich) doping increases *p*_*z*_ orbital contributions near the Fermi level, introduces localized states within the band gap, and distorts the electronic band structure (cf. Figure S19iii), potentially influencing magnetic moments. Although less pronounced in the PDOS of bulk HOPG with N doping (cf. Figure 5bi), the s orbital peak appears more elongated in the conduction band (cf. Figure 5aii), indicating increased bonding around the nitrogen atom.

Nitrogen dopant concentration was engineered by removing three carbon atoms and incorporating two nitrogen atoms into the graphene hexagonal lattice, each bonding to three carbon atoms. A third nitrogen atom was introduced at the graphene edge, forming pyridinic-N, where it bonded to two carbon atoms in a six-membered ring^89^ (cf. Figure S1d, Supporting Information). Similar to reduced N-doped PDOS, the DOS increased at the Fermi level, shifting toward the conduction band (cf. Figure 6bi) but with split peaks, as reflected in Figure 6aii. This effect is unsurprising, as nitrogen doping enhances conductivity and reactivity, while localized states disrupt charge carrier mobility, introducing split peaks and electronic disorder (cf. Figure S20iii). The presence of pyridinic-N resulted in nitrogen forming two σ bonds with adjacent carbon atoms, contributing one electron to the delocalized π system and leaving a lone pair, which increases chemical reactivity, particularly for catalysis and adsorption.^89–95^ Additionally, the broadening of the s orbital peak (cf. Figure 6aii) in the conduction band, caused by increased doping, indicates significant orbital overlap and hybridization with neighboring atoms. This greater dispersion of energy states enhances electron mobility, while the broadening effect signifies defect-induced lattice distortion and variations in the local electronic environment.

When HOPG is doped with oxygen, the Fermi level shifts to the valence band (cf. Figure 5ci), as oxygen’s higher electronegativity and two additional valence electrons make it an electron acceptor. This p-type behavior arises from electron withdrawal from the carbon lattice, creating holes (p-type behavior), as reflected in the PDOS of oxygen defects (cf. Figure 5bii). Oxygen incorporation often forms functional groups like carbonyl (C=O) and epoxy (C–O–C) within the hexagonal lattice and at graphene edges.^96–98^ Its strong bonding affinity disrupts the π-conjugated system, affecting π and π* bands linked to *p*_*z*_ orbitals. Consequently, oxygen doping converts some *sp* ^2^-hybridized carbon into *sp* ^3^ hybridization, forming hydroxyl and epoxy groups. When carbon forms four single bonds, it cannot participate in the π electron system, disrupting the delocalized π network and typically reducing electrical conductivity. This effect manifests as localized midgap states in the band structure (Figures S20v–S20vi), becoming more dispersed with increased doping concentration. Surprisingly, despite this disruption, the band structure remained metallic, suggesting that parts of the material retained *sp* ^2^ hybridization, preserving limited conductivity.

Oxygen incorporation shifted *p*_*z*_ orbital energy levels below the Fermi level (cf. Figure 6bii), introducing midgap states (see Figure 6ci) near the valence band edge with sharpened peaks, particularly at higher oxygen doping levels (cf. Figure 6bii). These new states result from bonding and antibonding interactions with surrounding carbon atoms, primarily due to nonbonding *p*-orbitals from oxygen lone pairs. Additionally, oxygen substitution shifted *s*-orbital states above the Fermi level, closer to the conduction band edge (i.e., toward the valence band), indicating altered hybridization and energy level modifications. Oxygen-containing functional groups created polar sites on the otherwise nonpolar graphene surface, enhancing chemical reactivity while inducing structural distortions in the HOPG lattice. The conversion of *sp* ^2^ to *sp* ^3^ hybridized carbon atoms disrupted the planar graphene structure, affecting bond lengths and angles (cf. Table 1). These distortions are evident in the 3D band structures (cf. Figure 7iv and vi).

**Table 1:** Mean Atomic Distance Time Series of HOPG with Pristine, Vacancy, and Substitutional Defects at 10 ps

HOPG system		average distance (Å^2^)		^a^refs
no defect	pristine	1.427	1.420^b^ ^,^ ^c^	^83,84^
vacancy defect	reduced	2.560		
	increased	3.589		
substitutional	N-doped	1.417	1.465^b^	^85^
defect	O-doped	1.500	1.330–1.440^c^	^86^
	S-doped	1.733	1.771^b^, 1.820^c^	^87,88^

a Comparison is made with available experimental and first-principles (FP) calculation values.

b Expt.

c FP.

With six valence electrons, sulfur doping introduces a new electronic state near the Fermi level, where sulfur *p* orbitals overlap with the *p*_*z*_ orbitals, leading to hybridization (cf. Figure 5cii). This interaction disrupts the π and π* bands in HOPG while preserving the *sp* ^2^ hybridization of the surrounding carbon atoms.^99^ Sulfur acts as an electron donor, increasing the energy of *p*_*z*_ states and making them more prominent at the Fermi level (see Figure 5cii), similar to nitrogen doping. However, when sulfur is introduced at the graphene edge, it induces *sp* ^3^ hybridization in localized areas, previously observed for oxygen doping, disrupting the delocalized π electron system and affecting electrical conductivity. This effect is evident in the PDOS (cf. Figure 6cii), where a reduction in *p*_*z*_ peak orbitals is observed. Sulfur-containing functional groups, such as sulfides (C–S–C), can bond to the carbon atoms at the graphene edge, as shown in Figure S 1f (Supporting Information).^100^

The dual n-type and p-type behavior of sulfur doping arises from its bonding environment within HOPG. With six valence electrons, a larger atomic size, and weaker, more polar bonds than carbon–carbon bonds, sulfur induces distortions in the *sp* ^2^ carbon network, sometimes affecting *p*_*y*_ orbitals. These perturbations can shift *p*_*y*_ states closer to the Fermi level, as seen in the PDOS for increased sulfur doping (cf. Figure 6cii). Additionally, sulfur’s complex electronic effects are evident in the slight Fermi level shift toward the valence band (cf. Figure 6di) and conduction band (cf. Figure 5di).

The 3D structures (cf. Figure 7v, Figure S21, Supporting Information) reveal significant structural distortion from pristine HOPG, caused by sulfur doping, which increased bond lengths to 1.733 Å around dopant sites. This defect introduces midgap states (cf. Figures S19iv, S20iv), which become more dispersed with increased doping, reflecting varying bonding environments in HOPG. Since sulfur doping can enhance chemical reactivity or conductivity, depending on doping concentration and bonding configuration, it can be engineered to create localized electronic states that promote adsorption and activate reactants. These modifications improve HOPG’s catalytic properties for the oxygen reduction reaction (ORR) and hydrogen evolution reaction (HER).^101–103^

Similar to vacancy defects, the total DOS (TDOS) and local DOS (LDOS) (cf. Figures S17–S18, Supporting Information) reveal higher states below the Fermi level (valence band) than in the conduction band for HOPG substitutional defects (N-doped, O-doped, S-doped). This occurs because dopants introduce localized electronic states that enhance DOS in the valence region, arising from lone-pair interactions and modifications to the π-conjugated system, which create defect states and lattice perturbations (cf. Figure 7, Figures S19–S21). While nitrogen doping introduces donor states near the conduction band edge, it predominantly increases valence band DOS. In contrast, oxygen and sulfur reduce available conduction band states (see PDOS – *vide infra*) by introducing localized states and altering the bonding environment, resulting in higher DOS in the valence band. However, these localized states, though they enhance valence band DOS, contribute little to delocalized electronic states necessary for electrical conductivity. Electrons in these states remain confined around the defect, primarily affecting the valence band and midgap states. This behavior is evident in the band structure, where pristine HOPG, initially semimetallic, transitions to a more metallic nature upon defect introduction.

### 3.3 Structural Properties

To further assess the performance of the trained force fields on the structural properties of the HOPG defect, radial distribution functions (RDFs) were computed, and the bond lengths were compared with available experimental data and FP calculations (see Table 1).

#### 3.3.1 RDF

While adopting the RDF formulations implemented in previous studies,^104–106^ Figures 9 and S22 (Supporting Information) present the RDFs detecting the interaction of HOPG with vacancy and substitutional defects, as well as with armchair and zigzag graphene nanoribbons (ZGNRs) on HOPG. As illustrated, RDFs detecting the interaction between carbon–carbon atoms of pristine HOPG emerge at distances of 1.40, 1.60, and 2.40 Å, corresponding to the first peak, first minimum, and second peak, with noticeable convergence to a normalized value of unity at the *R*_CC_ distance of 12 Å (*grosso modo*). This is encouraging, considering that the RDF was sampled on a trajectory obtained from the machine learning force field (MLFF) simulation. When compared to both experimental and FP calculation values obtained at 1.42 Å for *R*_CC_ in refs 83 and 84, it is permissible to assert that the close resemblance in the values justifies the robustness of MLFF in accurately predicting the properties of solids.^23,28,107^ Furthermore, the noticeable sharpness and intensity accompanying the first peak evince the presence of strong covalent bonds existing between the carbon atoms in the hexagonal lattice, with subsequent intralayer peaks (see the second, third, and fourth) being less prominent than the first peak, yet still relatively protruding due to the ordered structure within the layers. In terms of the interlayer peak distance of 4.9 Å, the integral of *R*_CC_ emerged with a coordination number of ∼0.8, corresponding to the weak interactions between the graphene layers that are held together by weak van der Waals forces rather than the strong covalent bonds observed for the intralayer peaks. This outcome is expected and indicative of weaker, less specific interactions between the layers, where each carbon atom in one layer is loosely “coordinated” with atoms in the adjacent layers.^58^ The interlayer peaks, with low coordination numbers and split peaks at the tip, emphasize the layered nature of HOPG, where strong covalent bonds are mainly present within the layers, and the layers can slide over one another easily due to the weak nature of interactions between the layers, contributing to the lubricating properties of HOPG.^84,108–110^ Although one might anticipate a broader peak for intralayer peaks, misalignment in the adjacent graphene layers resulted in variations in the interlayer spacing, leading to split peaks.^83,109^ Nonetheless, while the coordination numbers highlight the anisotropic nature of HOPG, they reveal a high degree of order and strong bonding within the planes of the graphene layers through a well-defined coordination number. Conversely, the weak interactions between the layers result in a lower and less distinct coordination number.

**Figure 9:**
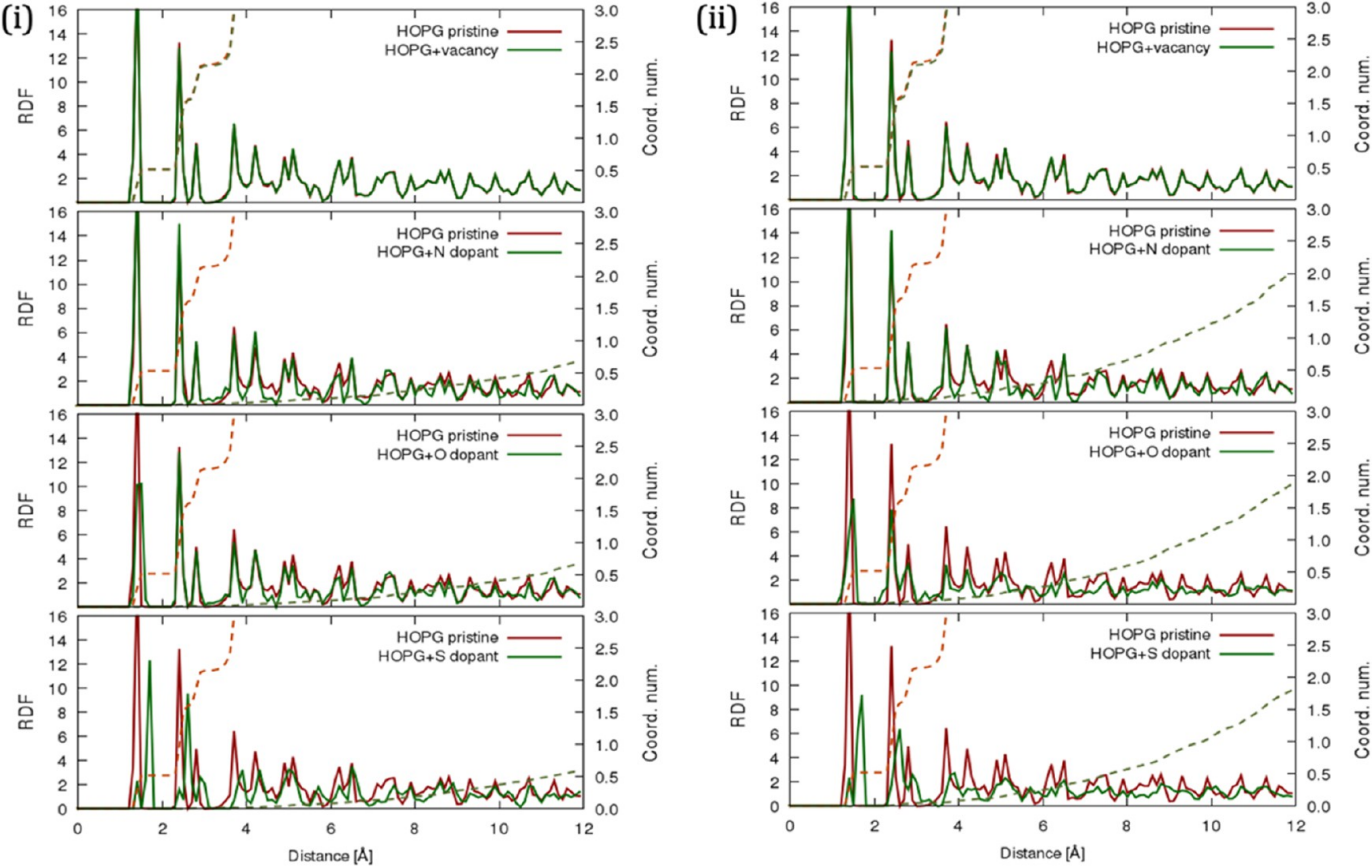
Radial distribution functions (RDF) detecting the interaction of HOPG with (i) reduced defects—vacancy defect (C–C), N dopant (C–N), O dopant (C–O), and S dopant (C–S); and (ii) increased defects—vacancy defect (C–C), N dopant (C–N), O dopant (C–O), and S dopant (C–S). A comparison is made with the RDF of the pristine HOPG surface structure.

Despite the presence of vacancy defects (cf. first plot of Figure 9i), the peaks are seen to embody regular periodicity at specific distances in the RDF, particularly within the layers of HOPG, thus indicating its crystalline nature, where atoms are not randomly distributed but are positioned at well-defined distances relative to one another. Similar to pristine HOPG, the first peak (i.e., intralayer) integrates to a coordination number of 3.0. Therefore, implying that the sp^2^ hybridization of the carbon atoms, where each carbon atom forms three σ-bonds with its neighbors and has a π-bond above and below the plane, contributes to the delocalized electron cloud. These observations are also likened to the RDFs of GNRs on HOPG (cf. Figure S22, Supporting Information), albeit with a slight increase in the tip of the peaks’ RDF for armchair GNRs on HOPG, and a decline in coordination numbers for both GNRs. This decline is particularly pronounced for armchair GNRs due to the reduced number of carbon atoms in the topmost graphene layer.

Notwithstanding the diminution in peak intensity to a normalized value of zero at 12 Å (*grosso modo*), it persists when HOPG is doped with nitrogen, oxygen, or sulfur, albeit with a slight shift in the RDF peaks (cf. Figure 9). Considering the carbon–nitrogen pair interaction (i.e., *R*_CN_), peaks at 1.40, 1.60, and 2.40 Å emerge for the first peak, first minimum, and second peak, respectively, for both defect concentrations, which closely resemble those of *R*_CC_. This similarity lends credence to nitrogen atoms occupying an analogous position in the graphene lattice when it replaces carbon atoms, owing to its similar atomic size and its ability to form three covalent bonds. Taking into account the carbon–oxygen pair interaction (i.e., *R*_CO_), peaks at 1.40, 1.80, and 2.40 Å emerge for the first peak, first minimum, and second peak in the reduced dopant concentration (see Figure 9i), while peaks at 1.50, 1.80, and 2.40 Å emerge, respectively, for the higher dopant concentration. These slight disparities observed at both defect concentrations can be rationalized as the result of sp^2^ to sp^3^ hybridization changes due to oxygen incorporation at the edges of the graphene layers with increased dopant concentration (cf. Figure 9ii).

Furthermore, the decline in the intensity of the C–O single bonds corresponding to the first intralayer peaks at 1.4 and 1.5 Å signifies a reduction in the overall crystalline order due to the disruption of the HOPG structure. When examining the carbon–sulfur pair interaction (i.e., *R*_CS_), the RDF appears highly perturbed, likely due to sulfur’s larger atomic size and its tendency to form longer bonds with carbon (see Table 1). Moreover, peaks at 1.40, 1.50, and 1.70 Å, corresponding to the first peak, first minimum, and second peaks of both sulfur doping concentrations, are seen to decline in intensity at higher doping levels, signifying that the increased presence of sulfur atoms disrupts the regular carbon lattice, further contributing to the decline in the peak intensity.

Generally, introducing vacancies and dopants into the HOPG lattice structure reduced the overall average coordination number of the carbon atoms. As for the intensity of intralayer peaks, it decreases with increasing distance but remains relatively sharp and distinct due to the crystalline order within the layers. In contrast, interlayer peaks exhibit a more rapid decline in intensity with distance, reflecting the diminishing influence of van der Waals interactions as one moves farther from the reference layer.

### 3.4 Dynamical Properties

#### 3.4.1 MSD and Diffusion Coefficient

We evaluated the mean square displacement (MSD) using the Fast Fourier Transform (FFT) algorithm described in refs^111^ and^112^ on a trajectory obtained from the machine-learning force field (MLFF) simulation for both pristine HOPG and HOPG with defects (vacancy and substitutional). Unsurprisingly, the MSDs in Figures S23–S24 (Supporting Information) were observed to remain constant with time due to the strong interatomic bonds in the solid lattice of the HOPG surfaces. Since the system is in a solid state, atoms are confined to specific positions while oscillating about their lattice sites. Additionally, the diffusion coefficient (*D*), evaluated from the limiting slope of the MSD for 100 ps, disclosed similar observations as the MSD (cf. Table S1, Supporting Information), thereby supporting the notion that within the graphene layer of HOPG, carbon atoms are held together by strong covalent bonds in a hexagonal lattice, making atomic diffusion within the layers (i.e., intralayer diffusion) extremely difficult under normal conditions. Despite the presence of defects (i.e., vacancy and substitutional), the overall diffusion of carbon atoms in the bulk HOPG remains limited.

In terms of the HOPG surface at the HOPG-water interface, the MSD in Figure S25 (Supporting Information) reveals complex behavior, transitioning over time from a ballistic to a random-walk régime, and then back to ballistic behavior. These behavioral patterns were also observed for TiO_2_ water interfaces.^113^ Taking into account the MSD of water molecules at the HOPG-water interface (see Figure S26, Supporting Information), these reveal considerably higher value than the HOPG surface—which is unsurprising given that water molecules positioned slightly further from the HOPG surface exhibit enhanced mobility due to less direct interactions with the surface. However, close to the surface, the MSD of water molecules is lower than that of the molecules in the bulk due to the formation of ordered structures and the molecules being more constrained. This analogy is supported by the decrease in the MSD value of pristine HOPG compared to the HOPG surface at the HOPG-water interface (see Table 2). Nonetheless, the tendency of water molecules to form ordered layers near the hydrophobic HOPG surface is due to the disruption of the hydrogen-bonding network at the interface,^105^ thereby causing the first few layers of water molecules to be more structured, forming an interfacial layer where mobility is reduced, and the MSD is generally lower than that of bulk water (i.e., water molecules positioned at a distance from the surface where the surface’s influence diminishes), as reported thereon.

**Table 2:** Dynamical Properties of Pristine HOPG and the HOPG-Water Interface: Mean Squared Displacement (MSD), Å^2^; and Diffusion Coefficient, *D*

	MSD (Å^2^)	*D* (cm^2^/s)
pristine HOPG	0.0279	3.607 × 10^–08^
HOPG interface	0.1168	5.673 × 10^–08^
water interface	8.9961	3.437 × 10^–06^

#### 3.4.2 Mean Atomic Distance Time Series

As previously discussed, vacancy defects influence the electronic structure of pristine HOPG in several ways, such as the formation of midgap or defect states, disruption of the π-conjugated network, modification of the band structure, and the potential introduction of magnetic moments. Given this, we sampled a trajectory from the MLFF simulation to determine the size of the vacancy defect concentration and the bond distance between the dopants and carbon atoms. Figure 1 depicts the positions of atoms or bonds within the structural configurations, while Section S3.4.2 (Supporting Information) briefly illustrates the mean atomic distance time series shown in Figure 10 for HOPG with vacancy defects and dopants. From Table 1, we confirm that increasing the atom distance from 1.427 Å (which closely agrees with ref 114) to 2.56 Å for reduced vacancy defects and 3.589 Å for higher vacancy defects led to a more pronounced alteration in the electronic structure, where pristine HOPG transformed from its intrinsic semimetallic to metallic behavior.

**Figure 10:**
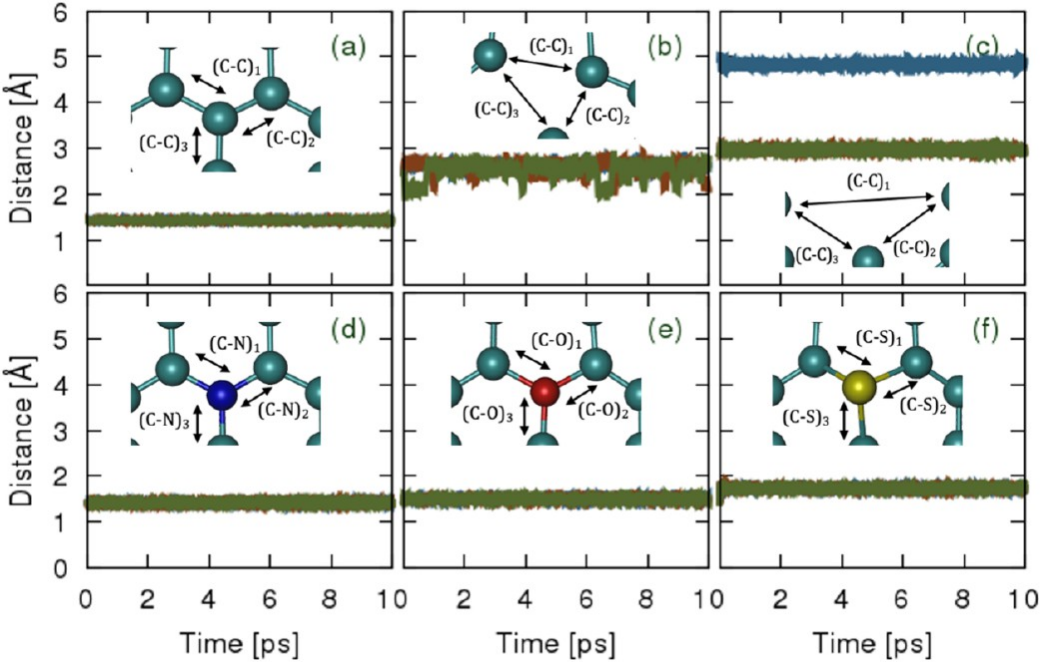
Mean atomic distance time series for HOPG with vacancy defects and dopants, tracking the evolution of key bond distances over time with (a) pristine, (b) reduced vacancy defect, (c) increased vacancy defect, (d) N dopant, (e) O dopant, and (f) S dopant.

Similarly, the introduction of dopants within the topmost graphene layer of HOPG influenced the electronic structure of HOPG by altering the overlap of *p*_*z*_ orbitals, introducing new states within the band gap, and modifying the band structure and density of states (DOSs). To this end, after sampling the distances from a trajectory obtained via the MLFF simulation, we affirm from Table 1, where comparisons are made with available data (see references herein)—that the shorter bonds associated with C–N bonds (i.e., 1.417 Å) increased the density of states—causing the Fermi level to shift upward toward the conduction band, potentially enhancing HOPG’s electrical conductivity, although this is not a definitive conclusion from the present study. In contrast, C–O bonds with an average bond distance of 1.5 Å led to the occurrence of localized midgap states and the conversion of *sp* ^2^ to *sp* ^3^ hybridized carbon atoms, which disrupted the delocalized π electron network, typically reducing electrical conductivity but increasing the reactivity of the HOPG structure. Longer bonds related to C–S bonds (i.e., 1.733 Å) introduced localized defect states within the band gap, and due to their complex bonding behavior, they influenced the overall conductivity and chemical reactivity of the structure, depending on the doping concentration and bonding environment in HOPG’s carbon lattice.

## 4 Conclusions

In this study, we demonstrated a comprehensive computational framework for defect engineering in highly oriented pyrolytic graphite (HOPG) by combining *ab initio* density functional theory (DFT) with state-of-the-art on-the-fly machine-learning force fields (MLFFs).^23,28,37^ We systematically investigated the structural, electronic, and dynamical properties of pristine HOPG, the HOPG-water interface, HOPG with vacancy defects, doped HOPG (N, O, S), and HOPG interfaced with armchair and zigzag graphene nanoribbons (GNRs).

The generated MLFFs enabled efficient and accurate prediction of material behavior across a range of defected and interfacial configurations, significantly reducing the need for repeated first-principles (FP) calculations. By incorporating the Bayesian error estimation, the training protocol adaptively bypassed unnecessary first-principles calculations, achieving near real-time learning with substantial computational savings.

Our findings revealed that vacancy defects and substitutional dopants substantially modified the electronic structure of HOPG by introducing midgap states, shifting the Fermi level, altering orbital hybridization, disrupting the π-conjugated network, and inducing localized magnetism. These changes directly influence the material’s electronic transport, surface reactivity, and interfacial behavior, while the MLFF-based simulations accurately captured dynamical stability and atomic-level interactions

Importantly, the insights from this work offer practical strategies for tuning the functional properties of HOPG through defect engineering. Specifically, nitrogen and sulfur doping enhance electrical conductivity and chemical activity, supporting improved charge transport in energy storage systems such as supercapacitor electrodes and battery anodes. Oxygen and sulfur functionalities increase surface polarity and catalytic potential, enabling enhanced electrocatalytic performance in reactions like oxygen reduction and hydrogen evolution reactions (ORR/HER). Defect-induced midgap states and tunable band gaps, along with localized states introduced by graphene nanoribbons (GNRs) and water interfaces, provide pathways for engineering the electronic landscape of HOPG—making it suitable for nanoelectronics components such as field-effect transistors (FETs) and low-power logic devices. Furthermore, interface-driven band bending and localized electronic states enhance surface sensitivity and tunability, suggesting promising applications in environmental sensing, wettability control, and functional coatings for flexible and corrosion-resistant surfaces.

In summary, this study highlights how the integration of MLFFs and DFT bridges fundamental electronic structure analysis with practical, application-driven design. It lays the groundwork for using machine learning-enhanced simulations to engineer next-generation carbon-based materials; the approach is scalable to other layered or carbon-based materials, accelerating the development of next-generation functional materials for use in energy, catalysis, electronics, and sensing technologies.

## Supplementary Material



## Notes

Detailed explanation of band structures along high-symmetry paths in the Brillouin zone (Section S3.2.1) and description of mean atomic distance time series (Section S3.4.2); supercell models showcasing various configurations of HOPG, including structures with N, O, and S dopants; models with 3N, 3O, and 3S dopants; configurations with increased and reduced vacancy defects; pristine HOPG; and armchair and zigzag graphene nanoribbons (GNRs) on HOPG (Figures S1–S2); error analysis of the force field during the “on-the-fly” machine learning simulations for different HOPG configurations: pristine, reduced/increased vacancy defects, N, O, and S dopants (both reduced and increased), and armchair/zigzag GNRs on HOPG (Figures S3–S14); total density of states (TDOS) and local density of states (LDOS) for pristine HOPG, and HOPG with reduced and increased point defects, including vacancy, N, O, and S dopants (Figures S15–S18); electronic band structures of various HOPG configurations, such as the HOPG-water interface, increased/reduced vacancy defects, HOPG with reduced/increased O, N, and S dopants, armchair graphene nanoribbons (AGNRs) on HOPG, and zigzag graphene nanoribbons (ZGNRs) on HOPG (Figures S19–S20); 3D electronic band structure of HOPG with reduced S dopant (Figure S21); RDF analysis of zigzag graphene nanoribbons (ZGNRs) and armchair graphene nanoribbons (AGNRs) on HOPG (Figure S22); MSD analysis for HOPG with various point defects (reduced and increased), including vacancy, N, O, and S dopants, as well as pristine HOPG (Figures S23–S24); log-scale MSD for the HOPG surface and water molecules at the HOPG-water interface (Figures S25–S26); dynamical properties of pristine HOPG, vacancy and substitutional HOPG defect: mean squared displacement (MSD) in Å^2^; and diffusion coefficient (*D*) (Table S1) (PDF)

## References

[1] Güell A. G., Tan S., Unwin P. R., Zhang G. (2015). Electrochemistry at highly oriented
pyrolytic graphite (HOPG): Toward a new perspective. Adv. Electrochem. Sci. Eng..

[2] Naveen G. J., SampathKumaran P., Badrinath P., Vynatheya S., Sailaja R. R., Seetharamu S., Deepthi M. V., Niranjan H. B. (2018). Role of
graphene oxide and addition of MOS_2_ in HDPE matrix for
improved tribological properties. IOP Conf.
Ser.:Mater. Sci. Eng..

[3] Singh Z. S. (2016). Applications
and toxicity of graphene family nanomaterials and their composites. Nanotechnol., Sci. Appl..

[4] Demidov D. V., Prosvirin I. P., Sorokin A. M., Bukhtiyarov V. I. (2011). Model AG/HOPG
catalysts: Preparation and STM/XPS study. Catal.
Sci. Technol..

[5] Oliveira
Brett A. M., Chiorcea Paquim A.-M. (2005). DNA imaged on a HOPG electrode surface
by AFM with controlled potential. Bioelectrochemistry.

[6] Tran T.-H., Rodriguez R. D., Salerno M., Matković A., Teichert C., Sheremet E. (2021). Twisted graphene
in graphite: Impact
on surface potential and chemical stability. Carbon.

[7] Grigorieva I. G., Antonov A. A. (2003). HOPG as powerful
x-ray optics. X-Ray Spectrometry.

[8] Brülle T., Ju W., Niedermayr P., Denisenko A., Paschos O., Schneider O., Stimming U. (2011). Size-dependent electrocatalytic activity of gold nanoparticles
on HOPG and highly boron-doped diamond surfaces. Molecules.

[9] Blanford C. F., Armstrong F. A. (2006). The pyrolytic graphite surface as
an enzyme substrate:
Microscopic and spectroscopic studies. J. Solid
State Electrochem..

[10] Talu, S. ; Dallaev, R. ; Sobola, D. Evaluation of surface characteristics of highly oriented pyrolitic graphite DEStech Transactions on Social Science, Education and Human Science, (amse), 2018, 10.12783/dtssehs/amse2018/24828.

[11] Uki K., Kato M., Harako S., Zhao X. (2014). AFM induced local oxidation
of HOPG. MRS Proc..

[12] Mandal B., Sarkar S., Pramanik A., Sarkar P. (2013). Theoretical prediction
of a new two-dimensional carbon allotrope and NDR behaviour of its
one-dimensional derivatives. Phys. Chem. Chem.
Phys..

[13] Zhou Y., Holme T., Berry J., Ohno T. R., Ginley D., O’Hayre R. (2010). Dopant-induced
electronic structure modification of
HOPG surfaces: Implications for high activity fuel cell catalysts. J. Phys. Chem. C.

[14] Park S., Suh Y., Floresca H., Kim M. (2009). HRTEM observation of mechanically
exfoliated graphene from natural graphite and HOPG. ECS Trans..

[15] Cisternas E., Stavale F., Flores M., Achete C. A., Vargas P. (2009). First-Principles
Calculation and scanning tunneling microscopy study of highly oriented
pyrolytic graphite (0001). Phys. Rev. B.

[16] Pantin V., Avila J., Valbuena M. A., Esquinazi P., Dávila M. E., Asensio M. C. (2006). Electronic properties
of high oriented
pyrolitic Graphite: Recent discoveries. J. Phys.
Chem. Solids.

[17] Zhang J., Wang E. (1995). STM investigation of HOPG Superperiodic
features caused by Electrochemical
Pretreatment. J. Electroanal. Chem..

[18] Lang W., Philipp A., Seeger K. (1985). Galvanomagnetic Properties of ASF5
-intercalated highly-oriented pyrolytic graphite (HOPG). Mol. Cryst. Liq. Cryst..

[19] Ray S. C. (2015). Application
and uses of graphene oxide and reduced graphene oxide. Appl. Graphene Graphene-Oxide Based Nanomater..

[20] Dasari
Shareena T. P., McShan D., Dasmahapatra A. K., Tchounwou P. B. (2018). A review on graphene-based nanomaterials in biomedical
applications and risks in environment and health. Nano-Micro Lett..

[21] Pylypenko S., Queen A., Neyerlin K. C., Olson T., Dameron A., O’Neill K., Ginley D., Gorman B., Kocha S., Dinh H. N., Gennett T., O’Hayre R. (2010). The role of
nitrogen doping on durability in the Pt-Ru/HOPG system. ECS Trans..

[22] Mechler, Heszler Á., Reimann C., Révész K., Bor Z. (1998). Cantilever flexure, adhesive\attractive
and lateral force measurements on highly-oriented pyrolytic graphite
by scanning force microscopy. Vacuum.

[23] Jinnouchi R., Lahnsteiner J., Karsai F., Kresse G., Bokdam M. (2019). Phase transitions
of hybrid perovskites simulated by machine-learning force fields trained
on the fly with Bayesian inference. Phys. Rev.
Lett..

[24] Jacobsen T. L., Jørgensen M. S., Hammer B. (2018). On-the-fly machine learning of atomic
potential in density functional theory structure optimization. Phys. Rev. Lett..

[25] Miwa K., Ohno H. (2017). Interatomic potential
construction with self-learning and Adaptive
Database. Phys. Rev. Mater..

[26] Podryabinkin E. V., Shapeev A. V. (2017). Active learning
of linearly parametrized interatomic
potentials. Comput. Mater. Sci..

[27] Li Z., Kermode J. R., De Vita A. (2015). Molecular
dynamics with on-the-fly
machine learning of quantum-mechanical forces. Phys. Rev. Lett..

[28] Jinnouchi R., Karsai F., Kresse G. (2019). On-the-fly
machine learning force
field generation: Application to melting points. Phys. Rev. B.

[29] Unke O. T., Chmiela S., Sauceda H. E., Gastegger M., Poltavsky I., Schütt K. T., Tkatchenko A., Müller K.-R. (2021). Machine Learning Force Fields. Chem. Rev..

[30] Kresse G., Joubert D. (1999). From Ultrasoft pseudopotentials to
the projector augmented-wave
method. Phys. Rev. B.

[31] Blöchl P. E. (1994). Projector
augmented-wave method. Phys. Rev. B.

[32] Kresse G., Furthmüller J. (1996). Efficient iterative schemes for ab initio total-energy
calculations using a plane-wave basis set. Phys.
Rev. B.

[33] Grimme S., Antony J., Ehrlich S., Krieg H. (2010). A consistent
and accurate
ab initio parametrization of density functional dispersion correction
(DFT-D) for the 94 elements H-Pu. J. Chem. Phys..

[34] Tocci G., Joly L., Michaelides A. (2014). Friction of
water on graphene and
hexagonal boron nitride from ab initio methods: Very different slippage
despite very similar interface structures. Nano
Lett..

[35] Maekawa Y., Sasaoka K., Yamamoto T. (2018). Structure of water clusters on graphene:
A classical molecular dynamics approach. Jpn.
J. Appl. Phys..

[36] Ohto T., Tada H., Nagata Y. (2018). Structure and dynamics of water at
water–graphene and water–hexagonal boron-nitride sheet
interfaces revealed by ab initio sum-frequency generation spectroscopy. Phys. Chem. Chem. Phys..

[37] Jinnouchi R., Karsai F., Verdi C., Asahi R., Kresse G. (2020). Descriptors
representing two- and three-body atomic distributions and their effects
on the accuracy of machine-learned inter-atomic potentials. J. Chem. Phys..

[38] Evans D. J. (1983). Computer
‘“experiment”’ for nonlinear thermodynamics
of Couette Flow. J. Chem. Phys..

[39] Allen, M. P. ; Tildesley, D. J. Computer Simulation of Liquids; 2017.

[40] Hoover W. G., Ladd A. J., Moran B. (1982). High-strain-rate plastic flow studied
via nonequilibrium molecular dynamics. Phys.
Rev. Lett..

[41] Klimeš J., Bowler D. R., Michaelides A. (2010). Chemical accuracy
for the van der
waals density functional. J. Phys.: Condens.
Matter.

[42] Klimeš J., Bowler D. R., Michaelides A. (2011). Van der waals
density functionals
applied to solids. Phys. Rev. B.

[43] Dev P., Agrawal S., English N. J. (2013). Functional
assessment for predicting
charge-transfer excitations of dyes in complexed state: A study of
triphenylamine-donor dyes on titania for dye-sensitized solar cells. J. Phys. Chem. A.

[44] Dion M., Rydberg H., Schröder E., Langreth D. C., Lundqvist B. I. (2004). Van der
waals density functional for general geometries. Phys. Rev. Lett..

[45] Román-Pérez G., Soler J. M. (2009). Efficient
implementation of a van der waals density
functional: Application to double-wall carbon nanotubes. Phys. Rev. Lett..

[46] Peng H., Yang Z.-H., Perdew J. P., Sun J. (2016). Versatile van der waals
density functional based on a meta-generalized gradient approximation. Phys. Rev. X.

[47] Gillan M. J., Alfè D., Michaelides A. (2016). Perspective:
How good is DFT for
water?. J. Chem. Phys..

[48] Zhang C., Wu J., Galli G., Gygi F. (2011). Structural and vibrational properties
of liquid water from van der waals density functionals. J. Chem. Theory Comput..

[49] Wang J., Román-Pérez G., Soler J. M., Artacho E., Fernández-Serra M.-V. (2011). Density,
structure, and dynamics
of water: The effect of van der waals interactions. J. Chem. Phys..

[50] Humphrey W., Dalke A., Schulten K. (1996). VMD: Visual molecular dynamics. J. Mol. Graphics.

[51] Bird J. P. (2002). Semiconductors:
An introduction. Encyclopedia Mater.: Sci. Technol..

[52] Ibach, H. ; Lüth, H. Solid-State Physics. In An Introduction to Principles of Materials Science, 4th ed.; Springer-Verlag, 2009.

[53] Setyawan W., Curtarolo S. (2010). High-throughput electronic band Structure Calculations:
Challenges and Tools. Comput. Mater. Sci..

[54] Tang N., Tang T., Pan H., Sun Y., Chen J., Du Y. (2020). Magnetic properties of graphene. Spintronic
2D Materials.

[55] Hayami S., Kusunose H., Motome Y. (2016). Emergent spin-valley-orbital
physics
by spontaneous parity breaking. J. Phys.: Condens.
Matter.

[56] Lechner C., Pannier B., Baranek P., Forero-Martinez N. C., Vach H. (2016). First-Principles Study of the structural,
electronic, dynamic, and
mechanical properties of HOPG and diamond: Influence of Exchange–Correlation
functionals and dispersion interactions. J.
Phys. Chem. C.

[57] Kundu R., Mishra P., Sekhar B. R., Maniraj M., Barman S. R. (2012). Electronic
structure of single crystal and highly oriented pyrolytic graphite
from Arpes and Kripes. Phys. B.

[58] Yang G., Li L., Lee W. B., Ng M. C. (2018). Structure of graphene and its disorders:
A Review. Sci. Technol. Adv. Mater..

[59] Rockett, A. Materials Science of Semiconductors; Springer Science & Business Media, 2008.

[60] Ma J., Zhong W., You L., Pei Y., Lu C., Xiao Z., Shen Z., Jiang X., Qian N., Liu X., Zhang S. (2022). Band bending caused by forming heterojunctions in Cu-Cu2O/RGO-NH2
semiconductor materials and surface coordination of N-methylimidazole,
and the intrinsic nature of synergistic effect on the catalysis of
selective aerobic oxidation of alcohols. Appl.
Surf. Sci..

[61] Wang C., Lai J., Chen Q., Zhang F., Chen L. (2021). *In operando* visualization of interfacial band bending in photomultiplying organic
photodetectors. Nano Lett..

[62] Haring A. J., Ahrenholtz S. R., Morris A. J. (2014). Rethinking Band Bending at the P3HT–TiO2
Interface. ACS Appl. Mater. Interfaces.

[63] Yang Q., Zhang N., Zhang Q., Zhang J.-Y., Fang Y.-Z., Zhou M. (2022). Band bending induced
charge redistribution on the amorphous MIL-53­(AL)/Co-LDH
conjunction to boost the supercapacitive and Oxygen Evolution Performance. Electrochim. Acta.

[64] Wen X., Fan X.-T., Jin X., Cheng J. (2023). Band alignment of 2D
material–water interfaces. J. Phys. Chem.
C.

[65] Oulhakem O., Rezki B., Belaiche M., Elansary M., Salameh B., Alsmadi A. K. M., Belrhiti Alaoui K. (2023). Effect of
water intercalation into
tungsten trioxide structure (WO3·XH2O) (x = 0,1,2): Correlation
between structure and photocatalytic performances. J. Catal..

[66] Wang X., Li G., Zhang L., Xiong F., Guo Y., Zhong G., Wang J., Liu P., Shi Y., Guo Y., Chen L., Chen X. (2022). The surface defects of HOPG induced
by low-energy AR+ ion irradiation. Appl. Surf.
Sci..

[67] Wang Y., Pochet P., Jenkins C. A., Arenholz E., Bukalis G., Gemming S., Helm M., Zhou S. (2014). Defect-induced magnetism
in graphite through neutron irradiation. Phys.
Rev. B.

[68] Nair R. R., Sepioni M., Tsai I.-L., Lehtinen O., Keinonen J., Krasheninnikov A. V., Thomson T., Geim A. K., Grigorieva I. V. (2012). Spin-half
paramagnetism in graphene induced by point defects. Nat. Phys..

[69] Chae J., Kim G. (2023). Effects of a single vacancy on electronic properties of a CA2N electride
bilayer. Appl. Surf. Sci..

[70] Jalilvand S., Sodagar S., Noorinejad Z., Karbaschi H., Soltani M. (2024). The effect of vacancy induced localized
states on the
thermoelectric properties of armchair bilayer phosphorene nanoribbons. Phys. Scr..

[71] Ren W., Xue W., Guo S., He R., Deng L., Song S., Sotnikov A., Nielsch K., van den
Brink J., Gao G., Chen S., Han Y., Wu J., Chu C.-W., Wang Z., Wang Y., Ren Z. (2023). Vacancy-mediated
anomalous
phononic and electronic transport in defective half-heusler zrnibi. Nat. Commun..

[72] Kumar S., Saklani R., Bhavya, Pratap S., Bhalla P. (2024). Effects of
vacancies
on quantum transport of zigzag graphene nanoribbons. Phys. Scr..

[73] Tan, C. S. Quadratic and Cubic Analysis of Vacancy-Induced Electrical Property Variations in Silicon, 2024, 10.2139/ssrn.4693314.

[74] Khan M. R., Gopidi H. R., Wlazło M., Malyi O. I. (2023). Fermi-level instability
as a way to tailor the properties of La3te4. J. Phys. Chem. Lett..

[75] Geisler B., Follmann S., Pentcheva R. (2022). Oxygen vacancy
formation and electronic
reconstruction in strained LaNiO_3_ and LaNiO_3_/LaAIO_3_. Phys. Rev. B.

[76] Rosendal V., Pryds N., Petersen D. H., Brandbyge M. (2024). Electron-vacancy
scattering in SrNbO_3_ and SrTiO_3_: A density functional
theory study with nonequilibrium Green’s functions. Phys. Rev. B.

[77] Rivas G., Yépez M., Mello P. A. (2023). Electron Transport and electron density
inside one-dimensional disordered conductors: An analysis of the electronic-levels
contribution. Waves Random Complex Media.

[78] Bok J. (1994). High-T C superconductivity
and electronic band Structure. J. Supercond..

[79] Aristov V.
Y., Lay G. L., Soukiassian P., Hricovini K., Bonnet J. E., Osvald J., Olsson O. (1994). Alkali-metal-induced
highest fermi-level pinning position above semiconductor conduction
band minimum. Europhys. Lett..

[80] Welch D. (2022). Normal-state
metallic behavior in contrast to superconductivity: An introduction. Handbook of Superconductivity.

[81] Kobayashi Y., Fukui K., Enoki T., Kusakabe K. (2006). Edge state on hydrogen-terminated
graphite edges investigated by scanning tunneling microscopy. Phys. Rev. B.

[82] Yazyev O. V. (2013). A guide
to the design of electronic properties of graphene nanoribbons. Acc. Chem. Res..

[83] Abdelaal S., Elmaghraby E. K., Abdelhady A. M., Youssf M., Rashad A. M., Bashter I. I., Helal A. I. (2020). The physical structure and surface
reactivity of graphene oxide. Diamond Relat.
Mater..

[84] Nguyen H. T. T., Tranh D. T. (2021). Evolution of the graphene layer in hybrid graphene/silicon
carbide heterostructures upon heating. European
Phys. J. D.

[85] Hughes E. W., Lipscomb W. N. (1946). The crystal structure of methylammonium chloride. J. Am. Chem. Soc..

[86] Harris C., Hardcastle F. (2015). Bond length
- bond valence relationships for carbon
- carbon and carbon - oxygen bonds. J. Arkansas
Academy Sci..

[87] Hargittai M., Hargittai I. (1974). An electron diffraction study of the molecular geometry
of dimethyl Sulphone. J. Mol. Struct..

[88] Trinajstić N. (1968). Calculation
of carbon-sulphur bond lengths. Tetrahedron
Lett..

[89] Ayiania M., Hensley A. J. R., Groden K., Garcia-Perez M., McEwen J.-S. (2019). Thermodynamic stability of nitrogen functionalities
and defects in graphene and graphene nanoribbons from first principles. Carbon.

[90] Roy D., Sarkar S., Bhattacharjee K., Panigrahi K., Das B. K., Sardar K., Sarkar S., Chattopadhyay K. K. (2020). Site specific
nitrogen incorporation in reduced graphene oxide using imidazole as
a novel reducing agent for efficient oxygen reduction reaction and
improved supercapacitive performance. Carbon.

[91] Liu S., Yang H., Huang X., Liu L., Cai W., Gao J., Li X., Zhang T., Huang Y., Liu B. (2018). Identifying
active sites of nitrogen-doped carbon materials for the co2 reduction
reaction. Adv. Funct. Mater..

[92] Miao H., Li S., Wang Z., Sun S., Kuang M., Liu Z., Yuan J. (2017). Enhancing the pyridinic
N content of nitrogen-doped graphene and
improving its catalytic activity for oxygen reduction reaction. Int. J. Hydrogen Energy.

[93] Zhang M., Xia C., Li L., Wang A., Cao D., Zhang B., Fang Q., Zhao X. (2024). Computational screening of pyrazine-based
graphene-supported transition metals as single-atom catalysts for
the nitrogen reduction reaction. Dalton Trans..

[94] Zhang X., Zhang X., Zhao S., Wang Y. Q., Lin X., Tian Z. Q., Shen P. K., Jiang S. P. (2021). Precursor modulated
active sites of nitrogen doped graphene-based carbon catalysts via
one-step pyrolysis method for the enhanced oxygen reduction reaction. Electrochim. Acta.

[95] Candu N., Man I., Simion A., Cojocaru B., Coman S. M., Bucur C., Primo A., Garcia H., Parvulescu V. I. (2019). Nitrogen-doped
graphene as metal free basic catalyst for coupling reactions. J. Catal..

[96] Vahdat M. T., Li S., Huang S., Bondaz L., Bonnet N., Hsu K.-J., Marzari N., Agrawal K. V. (2023). Mechanistic
insights on functionalization
of graphene with ozone. J. Phys. Chem. C.

[97] Hoyos-Ariza F. A., Prias-Barragan J. J., Galván D. H., Guerrero-Sánchez J., Ariza-Calderon H. (2023). Graphene nanostructures functionalization: Hydrogen
bonds and oxide coverage effect. Mater. Today
Commun..

[98] Tajima K., Isaka T., Yamashina T., Ohta Y., Matsuo Y., Takai K. (2017). Functional group dependence of spin magnetism in graphene oxide. Polyhedron.

[99] Wu H., Li W., Li W., Dai Y., Guo J., Wang S., Song J., Odunmbaku G. O., Zhang H., Boi F. S. (2022). Evidence
for increased metallicity arising from carbon-sulfur bonding and amorphization
effects in sulfur-doped pyrolytic graphite. Diamond Relat. Mater..

[100] Gu X., Zhang S., Hou Y. (2016). Graphene-based sulfur
composites
for energy storage and conversion in li-s batteries. Chin. J. Chem..

[101] Lee C. H., Nam E. B., Lee S. U. (2019). Enhanced
catalytic
activity of SOx -incorporated graphene for the hydrogen evolution
reaction. J. Mater. Chem. A.

[102] Kagkoura A., Pelaez-Fernandez M., Arenal R., Tagmatarchis N. (2019). Sulfur-doped
graphene/transition metal dichalcogenide heterostructured hybrids
with electrocatalytic activity toward the hydrogen evolution reaction. Nanoscale Adv..

[103] Ding Q., Cheng A., Ma L., Ding Y., Men D. (2020). In situ-generated NiCo@NiS nanoparticle
anchored S-doped carbon nanotubes
as dual electrocatalysts for oxygen reduction reaction and hydrogen
evolution reaction. Nanotechnology.

[104] Ajide M. T., English N. J. (2023). Nonequilibrium ab
initio molecular
dynamics simulation of water splitting at Fe2O3–hematite/water
interfaces in an external electric field. J.
Phys. Chem. C.

[105] Ajide M. T., English N. J. (2023). Effect of temperature
on the dipole
response, structural and dynamical properties of water under External
Electric Fields. J. Mol. Liq..

[106] Ajide M. T., O’Carroll D., English N. J. (2023). Structural, dynamical
and dielectric properties of water in contact with TiO2 surfaces via
molecular-dynamics simulations. Mol. Phys..

[107] Jinnouchi R., Karsai F., Kresse G. (2020). Making free-energy
calculations routine: Combining first principles with machine learning. Phys. Rev. B.

[108] Jin B., Chen G., He Y., Zhang C., Luo J. (2023). Lubrication
properties of graphene under harsh working conditions. Mater. Today Adv..

[109] Tomanik E., Christinelli W., Souza R. M., Oliveira V. L., Ferreira F., Zhmud B. (2023). Review of graphene-based materials
for Tribological Engineering Applications. Eng.

[110] Berman D., Erdemir A., Sumant A. V. (2014). Graphene: A new
emerging lubricant. Mater. Today.

[111] Wang V., Xu N., Liu J.-C., Tang G., Geng W.-T. (2021). VASPKIT: A user-friendly interface
facilitating high-throughput
computing and analysis using VASP Code. Comput.
Phys. Commun..

[112] Kneller G. R., Keiner V., Kneller M., Schiller M. (1995). NMOLDYN: A
program package for a neutron scattering oriented analysis of molecular
dynamics simulations. Comput. Phys. Commun..

[113] Futera Z., English N. J. (2017). Exploring rutile (110) and Anatase
(101) TiO2 water interfaces by reactive force-field simulations. J. Phys. Chem. C.

[114] ERGUN S. (1973). Structure of graphite. Nat. Phys. Sci..

