# Supplementary material for: Machine Learning Force Field Predictions of Structural and Dynamical Properties in HOPG Defects and the HOPG-Water Interface with Electronic Structure Analysis
Source: ACS Omega. 2025 Jun 10;10(24):25962–80. doi: 10.1021/acsomega.5c02543 (PMC12198994; doi:10.1021/acsomega.5c02543)
Supplement: Supplementary file 1 [file ao5c02543_si_001.pdf]

Supplementary Material
For
Machine Learning Force Field Predictions of Structural and Dynamical Properties in HOPG Defects and the HOPG-Water Interface with Electronic Structure Analysis

Mary T. Ajide [1*], Parisa Naeiji [1], Joaquín Klug [2], and Niall J. English [1*]

[1]School of Chemical & Bioprocess Engineering, University College Dublin, Belfield, Dublin 4, Ireland
[2]Department of Life Sciences, Atlantic Technological University, ATU Sligo, Ash Lane, Sligo, F91 YW50, Ireland

* Corr. Authors – mary.ajide@ucd.ie ; niall.english@ucd.ie

Table of Contents

S3.2.1: Band Structure

S3.4.2: Mean Atomic Distance Time Series

Figure S1-S2: Supercell models showcasing various configurations of HOPG, including structures with N, O, and S dopants; models with 3N, 3O, and 3S dopants; configurations with increased and reduced vacancy defects; pristine HOPG; and armchair and zigzag graphene nanoribbons (GNRs) on HOPG

Figure S3-S14: Error analysis of the forcefield during the "on-the-fly" machine learning simulations for different HOPG configurations: pristine, reduced/increased vacancy defects, N, O, and S dopants (both reduced and increased), and armchair/zigzag GNRs on HOPG

Figure S15-S18: Total Density of States (TDOS) and Local Density of States (LDOS) for pristine HOPG, and HOPG with reduced and increased point defects, including vacancy, N, O, and S dopants

Figure S19-S20: Electronic band structures of various HOPG configurations, such as the HOPG-water interface, increased/reduced vacancy defects, HOPG with reduced/increased O, N, and S dopants, armchair graphene nanoribbons (AGNRs) on HOPG, and zigzag graphene nanoribbons (ZGNRs) on HOPG

Figure S21: 3D electronic band structure of HOPG with reduced S dopant

Figure S22: RDF analysis of zigzag graphene nanoribbons (ZGNRs) and armchair graphene nanoribbons (AGNRs) on HOPG

Figure S23-S24: MSD analysis for HOPG with various point defects (reduced and increased), including vacancy, N, O, and S dopants, as well as pristine HOPG

Figure S25-S26: Log-scale MSD for the HOPG surface and water molecules at the HOPG-water interface

Table S1: Dynamical properties of pristine HOPG, vacancy and substitutional HOPG defect: mean squared displacement (MSD), $Å^2$; and diffusion coefficient, D

S3.2.1 Band Structure

Pristine HOPG

Interestingly, the band structures shown in Figure S19-S20 disclose the electronic band energies along specific high-symmetry paths in the Brillouin zone. These paths are revealed to connect the points depicted in the plots in reciprocal space, where the electronic structure is crucial [1-2]. For pristine HOPG (cf. Figure S19i), the Γ-M-K-Γ path shows the Γ-M point transversing from the centre of the Brillouin zone to the midpoint of the hexagonal face edge. It explores transitions along a major symmetry direction within the plane of the HOPG layers, offering insights into in-plane electronic properties. The M-K point travels from the edge to a corner of the hexagonal Brillouin zone, examining the symmetries related to the edge and observing how the band structure changes along the zone's edge, which is paramount for understanding the angular dispersion of the HOPG hexagonal lattice structure [3]. The K-Γ segment completes the loop by returning to the centre, analysing shifts in electronic states as they transition from high-symmetry points back to the central point. In the Γ-A-L-H-A|L segment, the Γ-A point travels perpendicularly out-of-plane, away from the centre towards the zone boundary along the c-axis of the HOPG layer, which is essential for investigating interlayer interactions and conductivity in the perpendicular direction. The A-L point moves from a point on the c-axis to the edge, exploring how out-of-plane symmetries affect the electronic states. The path moving from L-H and back to the A point, afterwards revisiting L, offers an in-depth examination of out-of-plane band behaviour near the top of the Brillouin zone, crucial for understanding the behaviour of bands at vertical extremes. The return to the L point emphasises further analysis at this high-symmetry point, critical for probing the anisotropic effects in the HOPG layered structure [4]. Moreover, when the path transverses the final segment from M-H and then to H-K, it concentrates on transitions at the edges and corners of the Brillouin zone, essential for thoroughly mapping the electronic landscape of the HOPG hexagonal structure. This last segment enhances the understanding of pristine HOPG edge states and the electronic transitions among various high-symmetry points within the topological aspects of the Brillouin zone [2,4]. Probing the 3D band structure further, pristine HOPG exhibits a high degree of crystallographic orientation, where a near-invisible gap between the Highest Occupied Molecular Orbital (HOMO) and the Lowest Unoccupied Molecular Orbital (LUMO) is observed. This further affirms the semi-metallic nature of pristine HOPG, resulting in some states in the conduction band being lower in energy than states in the valance band, thereby facilitating electron movement and its metallic conductivity [5].

HOPG-Water Interface

Evidently, band bending caused a redistribution of states and may have led to the formation of interface states that are not present in pristine HOPG. These states arise due to the discontinuity

in the electronic structure at the boundary where HOPG and water intersect (see Figure S20i). When the HOPG surface interfaces with water molecules, the high-symmetry paths (Γ-X|Y-Γ-Z|R-Γ-T|U-Γ-V) in the Brillouin zone offer insight into how the electronic band structure was impacted, even though these paths do not correspond to recognised paths within the Brillouin zone for pristine HOPG, as mentioned earlier – *vide supra*. Nonetheless, considering the segment Γ-X|Y, X and Y typically denote specific points on the edge of the Brillouin zone, potentially corresponding to the principal axes in a cubic system [4]. In the case of HOPG, this is similar to transversing from Γ to M and returning, while examining the in-plane electronic characteristics. The segment Γ-Z|R indicates another in-plane examination, transitioning from the centre to various edge points, thereby investigating variations in the electronic structure along two distinct high-symmetry lines. When considering Γ-T|U, if T and U signify transitions along or across different Brillouin zone boundaries, this perhaps suggests a detailed examination involving both in-plane and possible out-of-plane (c-axis) transitions, especially if it includes movement towards A. The Γ-V path is involved in exploring shifts from the centre to a vertical or out-of-plane boundary, particularly relevant when assessing the impacts of water intercalation and its potential effects on electronic states perpendicular to the layers. In summary, the 3D structure plot (see Figure 7ix) visually represents how energy levels (bands) evolve across different points in the Brillouin zone.

Vacancy Defects

Considering the paths Γ-X|Y-Γ-Z|R-Γ-T|U-Γ-V, relating to the vacancy defects (see Figure S19ii and Figure S20ii), it indicates that the Γ-X|Y segments transition from the centre, Γ (where $k = 0$), toward edge points in the Brillouin zone, possibly towards points like M or K. Along this path, vacancy defects introduce localised defect states that manifest as extra energy states within the band gap or near the Fermi level. These states are presumed to have altered the typical semi-metallic behaviour of the HOPG layers, potentially opening a small gap or forming flat bands. In the Γ-Z|R segment, the Z point typically indicates a location along the c-axis, describing the out-of-plane direction. Although interlayer interactions in HOPG are generally weak, defects alter these van der Waals interactions, possibly introducing new states that impact the out-of-plane electronic structure. This path allows for examining variations in the band structure as it transitions from in-plane behaviour (near Γ) to out-of-plane behaviour (towards Z) when defects are present. Vacancies are observed to cause subtle shifts in interlayer coupling, particularly around defect sites. The Γ-T|U segment involves transitioning to high-symmetry points that illustrate band dispersion along specific crystallographic directions. With vacancies present, the crystal's symmetry is locally disrupted, potentially causing band splitting or the emergence of additional states along this path. Additionally, vacancy-induced states influence the curvature of conduction and valence bands, especially near the defect region, thereby resulting in band flattening or the appearance of mid-gap states. The final path, Γ-V, represents movement in reciprocal space toward another high symmetry point, possibly along a different axis within the graphene plane or perpendicular to it. The presence of vacancies modifies the band structure along this route by altering the local electronic structure, potentially generating resonant states near the Fermi level, leading to a decrease in electron conductivity in a particular direction due to increased scattering, as also reported for LDOS *(vide supra)*.

Due to the quasi-one-dimensional nature of GNRs, the armchair graphene nanoribbons (AGNRs) structure (cf. Figure S19v) depends on their width, where confinement leads to band opening,

even though bulk HOPG is reported to be almost gapless. The calculated segment, Γ-X|Y-Γ-Z|R-Γ-T|U-Γ-V from the band structure, and also akin to the aforementioned vacancy defect, describes the path Γ-X|Y, exploring the in-plane electronic characteristics of AGNRs in contact with the HOPG substrate. Since the HOPG substrate induces charge transfer that can alter the electronic states of AGNRs, this interaction resulted in shifts or distortions in the band edges, potentially decreasing the band gap, as observed. Additionally, van der Waals forces between the nanoribbons and HOPG subtly modify the electronic states close to the Fermi level. As AGNRs lie flat on the HOPG surface, the Γ-Z|R path examines how interactions between the nanoribbons and substrates influence the out-of-plane electronic states. While the interlayer coupling in HOPG is generally weak due to van der Waals forces, the nanoribbons induce changes at the interface, particularly due to local strain or distortions arising from lattice mismatch. Furthermore, the presence of HOPG impacted charge distribution within the AGNRs, potentially modifying the band structure along the out-of-plane direction. The Γ-T|U path plays a crucial role in analysing band dispersion across various crystallographic directions, with the orientation of AGNRs on HOPG resulting in degrees of dispersion (either flat or dispersive bands) along this direction. Factoring in the nanoribbons' specific orientation caused localised states to form along this path, as reflected in the changes in PDOS. Nonetheless, the Γ-V path uncovers surface or resonance states induced by the proximity of AGNRs to the HOPG substrate. Given that AGNRs possess edge states, especially along the armchair edge, their interactions with HOPG shift these edge states, as observed from the band structure. Consequently, the band alignment between AGNRs and HOPG was examined and found to have resulted in additional shifts observed in the Fermi level, leading to the creation of new band structure features peculiar to AGNRs on HOPG, as shown in the plot.

Considering zigzag graphene nanoribbons (ZGNRs) interfaced with the HOPG substrate, Figure S19vi reveals how the electronic properties of ZGNRs are modified by their interaction with HOPG, where the path Γ-Z-D-B-Γ-A-E-Z-C_2-Y_2-Γ differs compared to their AGNRs counterparts. As the path travels from Γ, representing the centre of the Brillouin zone where the wavevector $k = 0$ (i.e., electronic states at rest in reciprocal space [4]), to Z (a point along the out-of-plane c-axis direction in the Brillouin zone of HOPG), Γ-Z explores the interaction between the nanoribbons and the substrate. Notably, the weak van der Waals coupling between ZGNRs and HOPG slightly alters the edge states of ZGNRs, thus impacting the DOS near the Fermi level. However, because ZGNRs are quasi-1D systems [6], the band structure in the out-of-plane is expected to show minimal dispersion due to the weak interaction. Moreover, the Z-D-B path examines other high-symmetry points within the in-plane Brillouin zone, where points D and B correspond to high-symmetry locations along distinct directions in the 2D Brillouin zone (i.e., individual graphene layer). Given that ZGNRs exhibit significant edge states localised along their zigzag edges, Z-D-B highlights how the band structure evolves along various crystallographic directions. When ZGNRs are placed on HOPG, their interaction influences metallic edge states, potentially inducing slight energy shifts or hybridisation with the underlying HOPG states. As the Γ-A-E path moves from Γ to A (a high-symmetry point in the in-plane direction, near the edge of the Brillouin zone of HOPG) and then to E (a point that lies along a diagonal direction in the reciprocal space [56-57][2,4]), the Γ-A-E path experiences slight band splitting or band gap opening due to interaction with the HOPG substrate. This phenomenon can be inferred as the edge states of ZGNRs shifting slightly based on the existing work function between ZGNRs and HOPG, potentially causing charge transfer or screening effects [7-8]. The segment Z-C_2-Y_2 explores additional high-symmetry points along the in-plane directions of the Brillouin zone, where points C_2 and Y_2 are likely located along the edges or faces of the Brillouin zone, thereby probing the electronic states in various

crystallographic directions. The Z-C_2-Y_2 directions reveals how the metallic edge states of ZGNRs behave when interacting with HOPG. In addition, this path indicates how the flat bands of the edge states interact with the graphene-like states in HOPG, potentially giving rise to interface-induced states. The final path Y_2-Γ circles back to Γ, completing the loop by returning to the centre of the Brillouin zone. By revising the Γ point, insight into how the overall band structure of ZGNRs on HOPG evolves after passing through these high-symmetry points is shown in the plot, allowing assessment of whether notable changes in the Fermi level or band curvature are caused by the interaction between the nanoribbons and the substrate.

Substitutional Defects

With the exception of ZGNRs, similar paths for the band structure describing the high-symmetry points in the Brillouin zone for HOPG substitutional defects (N-doped, O-doped, S-doped) in Figures S19iii-S19iv and Figures S20iii-S20vi closely mimics those of HOPG vacancy defects. In any event, despite similar high-symmetry paths in the Brillouin zone reported for different degrees of the defect (i.e., increased or reduced), the electronic band structures disclose how any modification to the pristine HOPG structure influences the behaviour of electrons in specific directions of momentum space (the reciprocal space) within the Brillouin zone of the doped structure. The paths; Γ-X|Y-Γ-Z|R-Γ-T|U-Γ-V relating to the substitutional defect, highlight vividly the changes in the band structure due to doping.

Considering the segment, Γ-X|Y representing the exploration of the electronic states from the centre of the Brillouin zone (i.e., Γ point) towards its boundaries in various directions, the impact of nitrogen doping introduces n-type states near the Fermi level. Along the, Γ-X|Y path, the new electronic states emerge close to the Fermi level, indicating additional electrons contributed by the nitrogen dopants (see Figure S19iii and Figure S20iii of the Supplementary Material). In addition, this path reveals the shift in the Fermi level of band structure, potentially decreasing the band gap. Incorporating sulphur leads to localised states due to lattice distortions – appearing as either flat band or mid-gap states along this path, in addition to peaks in DOS (cf. Figure S19iv and S20iv). When doped with oxygen, p-type states (i.e., acceptor level) emerge thus introducing localised states (cf. Figure S20v-S20vi). Along Γ-X|Y, path, the band structure discloses band bending close to the dopant sites, altering the typical semi-metallic nature of HOPG.

Furthermore, using the movement of the Γ-Z|R path within the Brillouin zone to probe the out-of-plane direction along the c-axis and examine the coupling between the layers reveals alterations in the interlayer coupling due to the dopant being chemically bonded within the graphene layer. This alteration led to the appearance of new interlayer states and changes in the dispersion relation along the c-axis, potentially modifying the van der Waals gaps. Undoubtedly, slight alteration in the band dispersion affects the electronic properties perpendicular to the graphene planes caused by dopants that change the interlayer interactions. Moreover, when investigating different in-plane crystallographic directions, the Γ-T|U path introduces prevailing localised defect states in the band structure, where nitrogen causes a slight downward shift of the conduction band due to its electron-donating nature, while sulphur and oxygen introduce flat bands and new electronic states within the band gap. Quite evidently, these modifications are revealed by the change in band curvature from pristine to doped (see Figure S19-S20), thus indicating altered effective masses for charge carriers. Nonetheless, examining the Γ-V path through the Brillouin zone further indicates the effect of doping on the electronic states, where

the Fermi level shifts upward towards the conduction band for nitrogen-doped, while oxygen- and sulphur-doped HOPG display a prevailing presence of defect-induced electronic states.

S3.4.2 Mean Atomic Distance Time Series

The mean atomic distance time series provides a time-resolved analysis of how atomic distances fluctuate within the material over the course of the machine-learning force field (MLFF) simulation. It offers insights into structural stability, bonding dynamics, and electronic structure modifications due to defects and dopants.

In the context of HOPG vacancy defects and dopants, the mean atomic distance time series measures the evolution of bond distances over time, particularly for key atomic pairs (e.g., C-C, C-N, C-O, C-S). This helps in understanding how structural changes impact the electronic and mechanical properties of HOPG.

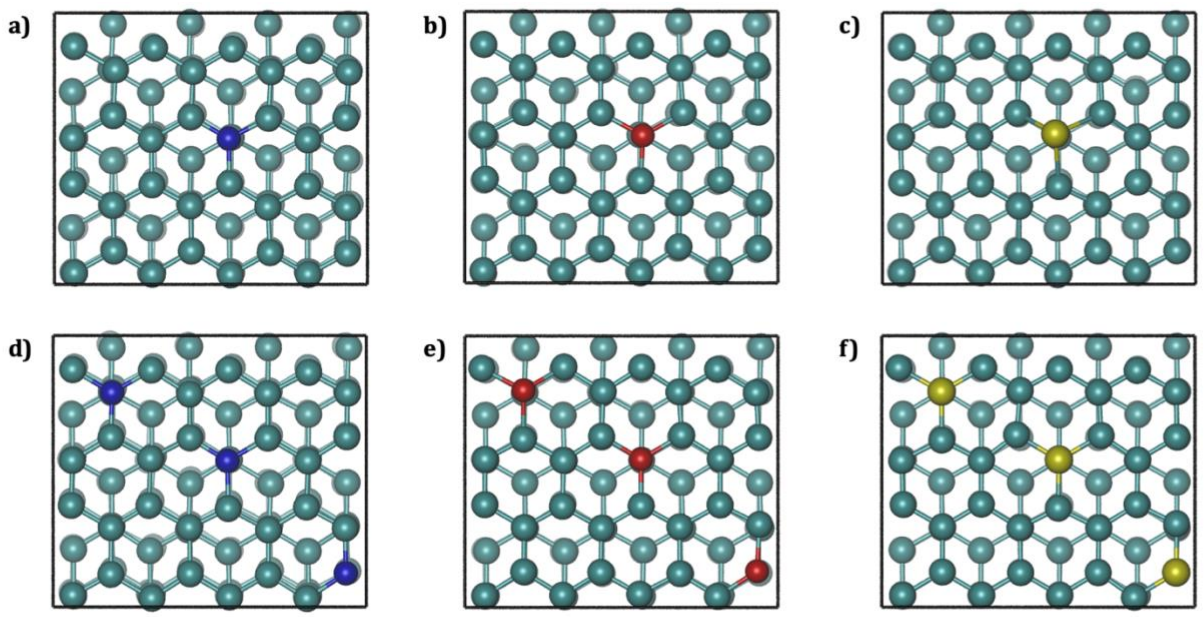

Figure S1 Supercell models of HOPG with a) N dopant, b) O dopant, c) S dopant, d) 3N dopant, e) 3O dopant, and f) 3S dopant.

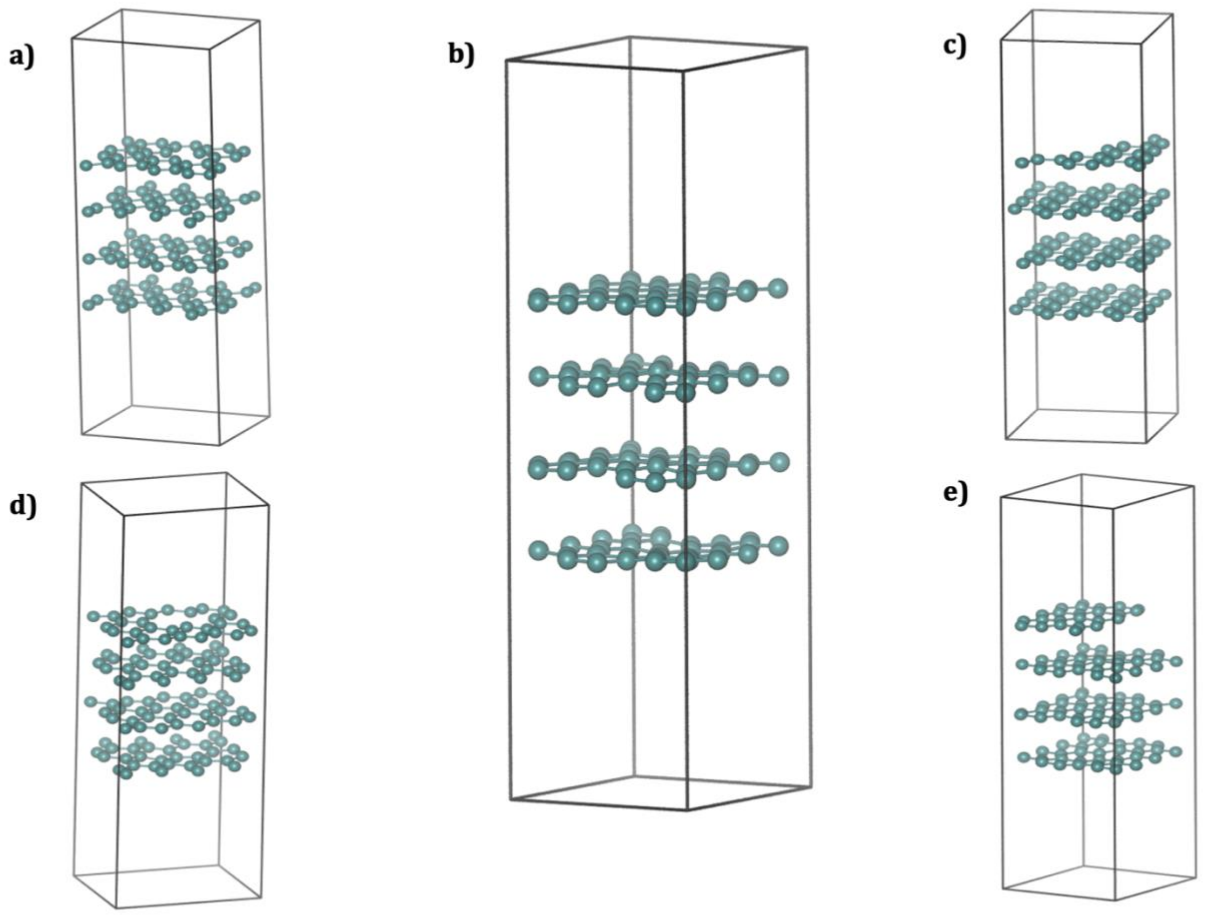

Figure S2 Supercell models of a) HOPG with increased vacancy defect, b) pristine HOPG, c) HOPG with reduced vacancy defect, d) armchair graphene nanoribbons (GNRs) and e) zigzag graphene nanoribbons (GNRs) on HOPG.

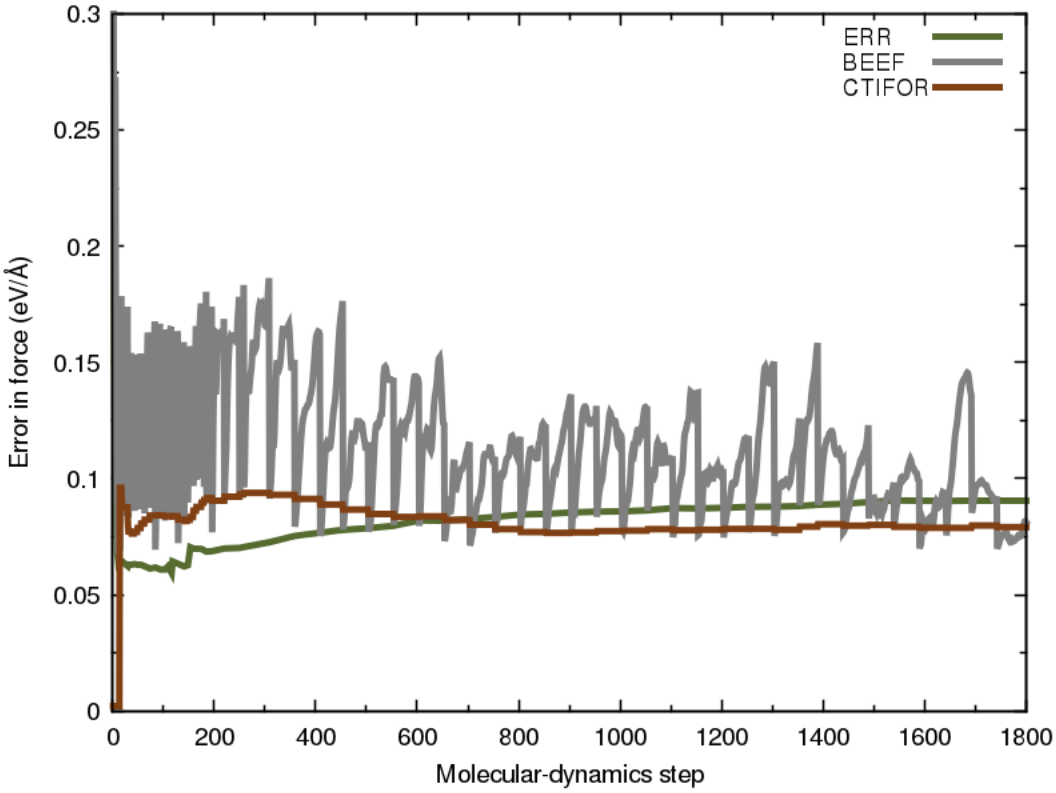

Figure S3. Error in the force field of the HOPG-water interface structure during the "on-the-fly" machine learning simulation. The errors in the plot are shown at different stages of the training process. The evolution of the Bayesian error estimates of forces is shown in dark grey colour, the root mean squared error of the predicted forces is represented in dark olive green colour, and the current threshold for maximum Bayesian error estimation of the forces is depicted in sienna colour.

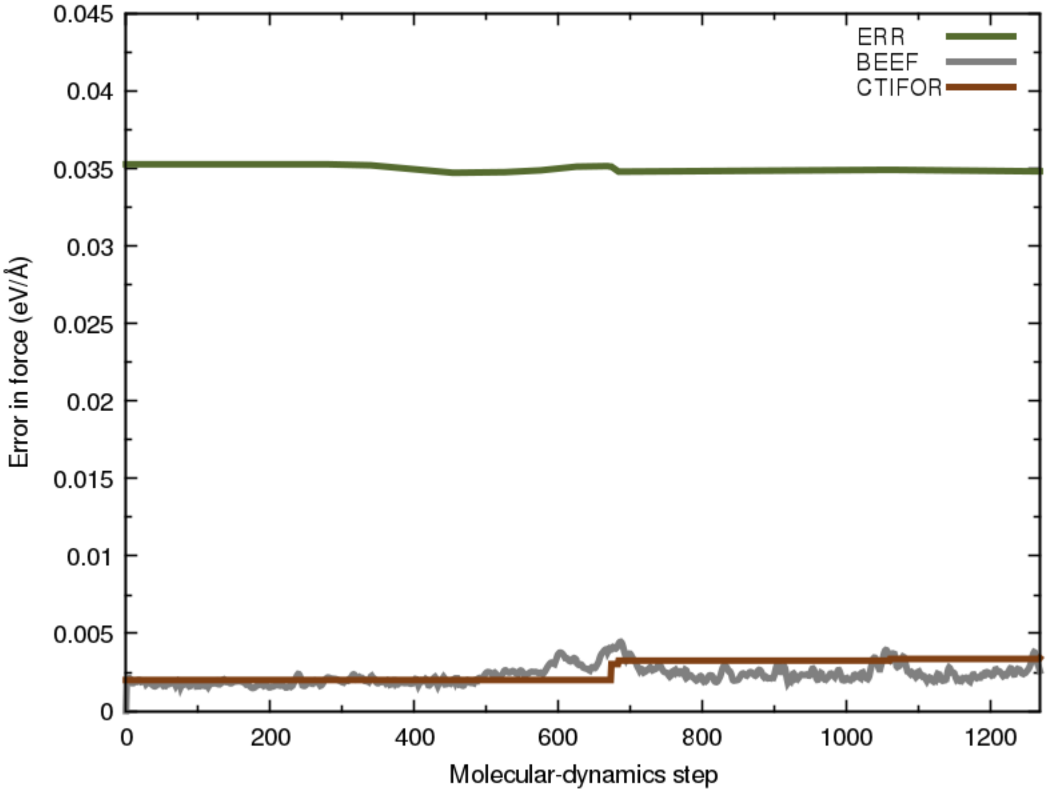

Figure S4. Error in the force field of the pristine HOPG structure during the "on-the-fly" machine learning simulation. The errors in the plot are shown at different stages of the training process. The evolution of the Bayesian error estimates of forces is shown in dark grey colour, the root mean squared error of the predicted forces is represented in dark olive green colour, and the current threshold for maximum Bayesian error estimation of the forces is depicted in sienna colour.

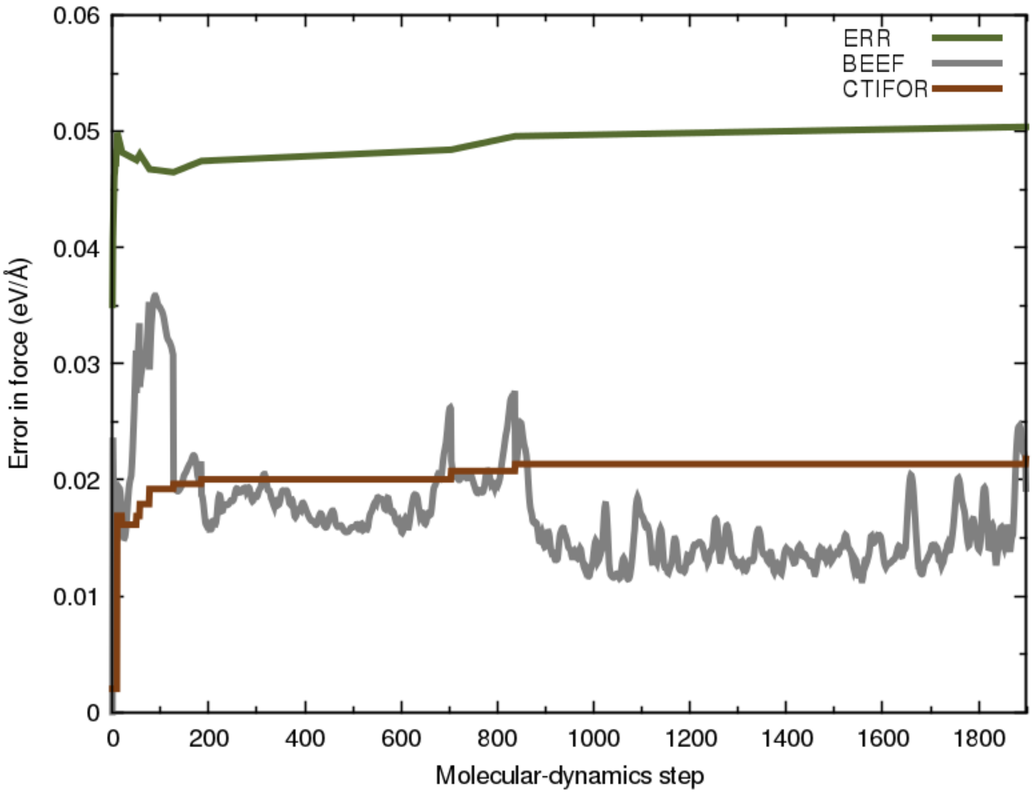

Figure S5. Error in the force field of the HOPG reduced vacancy defect structure during the "on-the-fly" machine learning simulation. The errors in the plot are shown at different stages of the training process. The evolution of the Bayesian error estimates of forces is shown in dark grey colour, the root mean squared error of the predicted forces is represented in dark olive green colour, and the current threshold for maximum Bayesian error estimation of the forces is depicted in sienna colour.

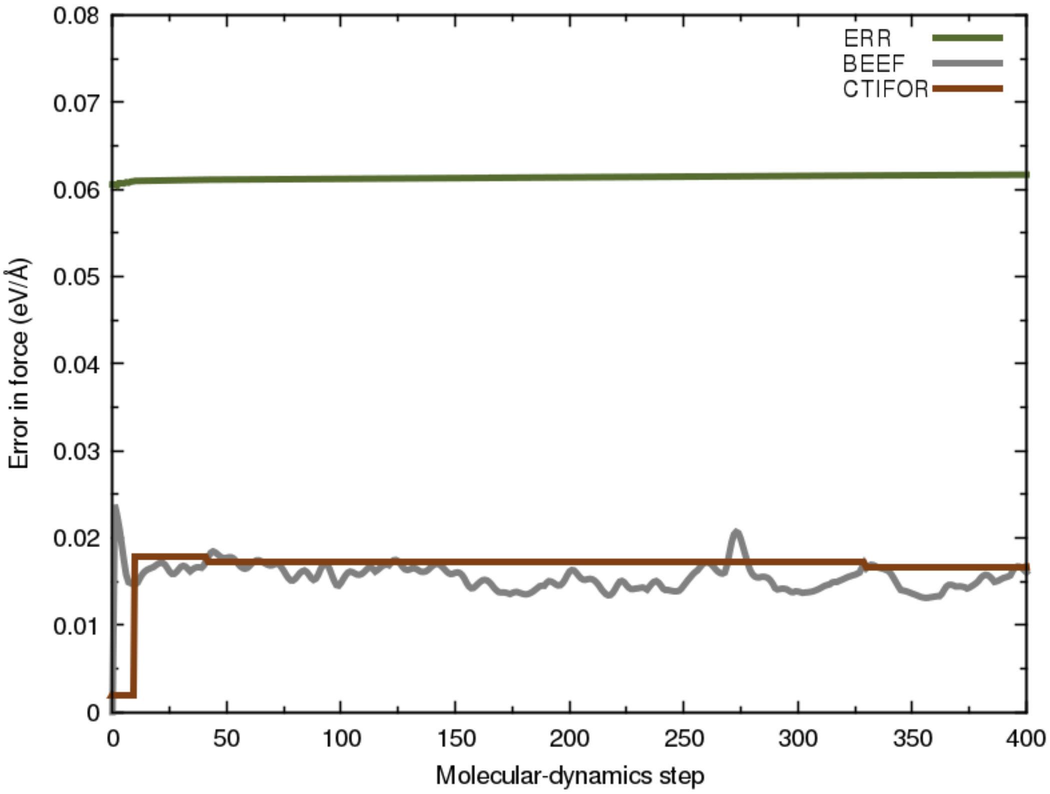

Figure S6. Error in the force field of the HOPG increased vacancy defect structure during the "on-the-fly" machine learning simulation. The errors in the plot are shown at different stages of the training process. The evolution of the Bayesian error estimates of forces is shown in dark grey colour, the root mean squared error of the predicted forces is represented in dark olive green colour, and the current threshold for maximum Bayesian error estimation of the forces is depicted in sienna colour.

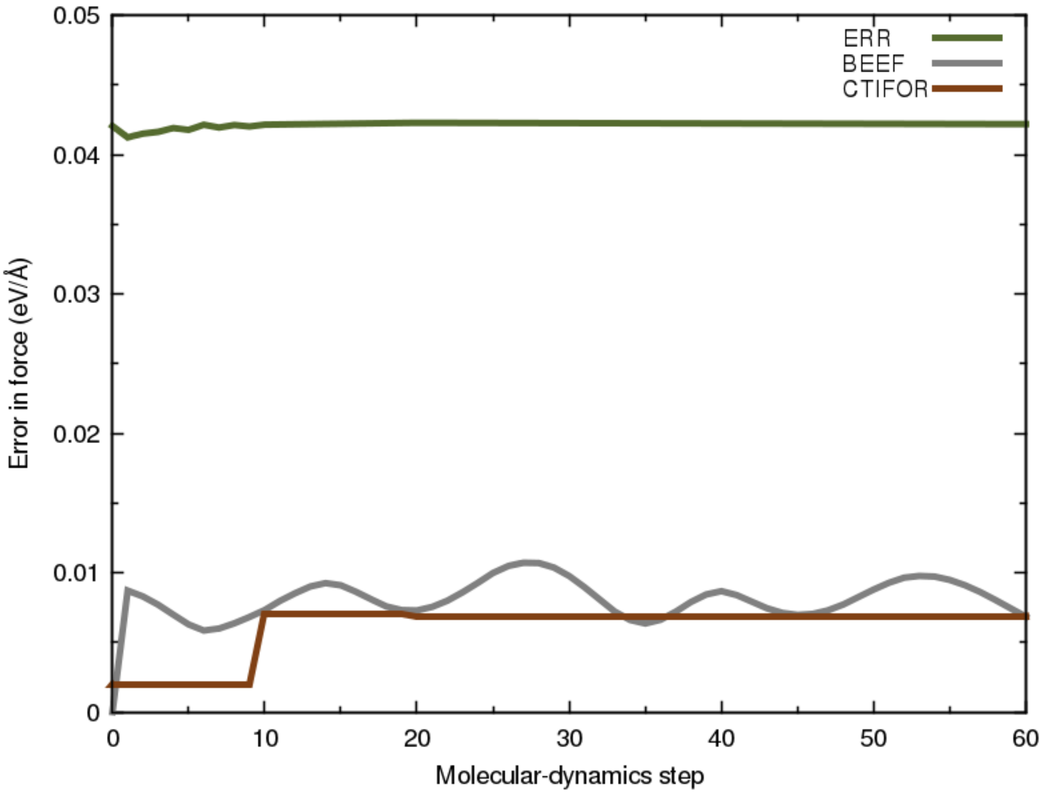

Figure S7. Error in the force field of the HOPG reduced N-doping structure during the "on-the-fly" machine learning simulation. The errors in the plot are shown at different stages of the training process. The evolution of the Bayesian error estimates of forces is shown in dark grey colour, the root mean squared error of the predicted forces is represented in dark olive green colour, and the current threshold for maximum Bayesian error estimation of the forces is depicted in sienna colour.

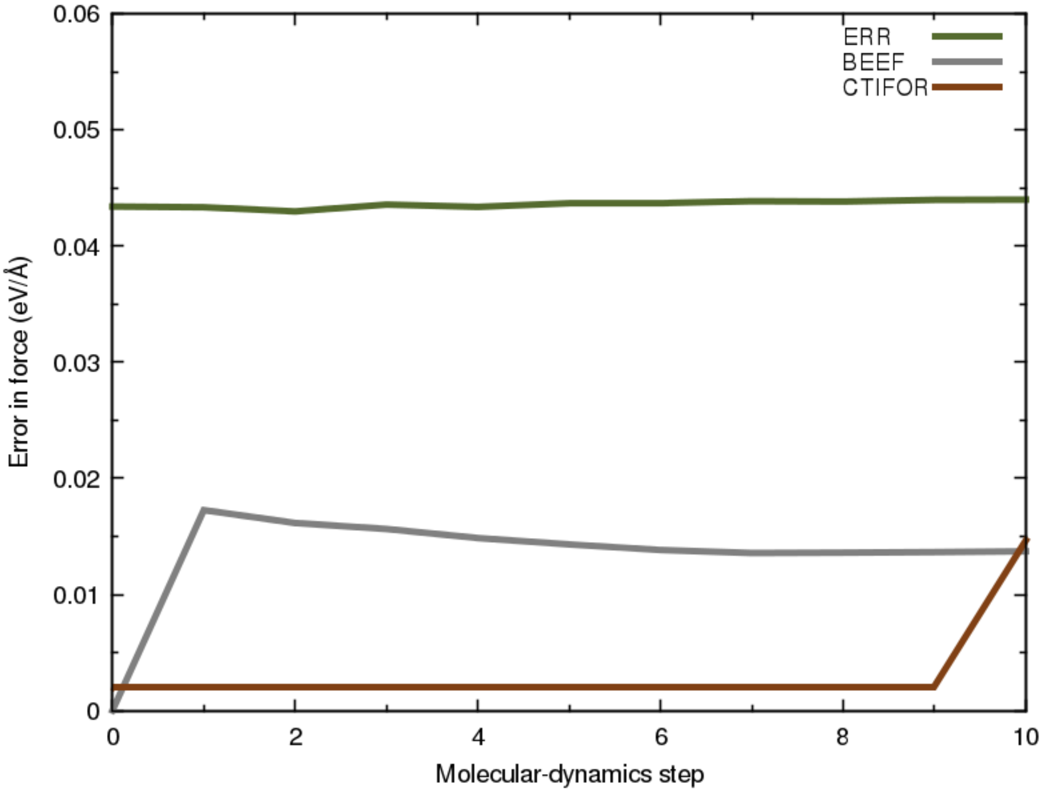

Figure S8. Error in the force field of the HOPG increased N-doping structure during the "on-the-fly" machine learning simulation. The errors in the plot are shown at different stages of the training process. The evolution of the Bayesian error estimates of forces is shown in dark grey colour, the root mean squared error of the predicted forces is represented in dark olive green colour, and the current threshold for maximum Bayesian error estimation of the forces is depicted in sienna colour.

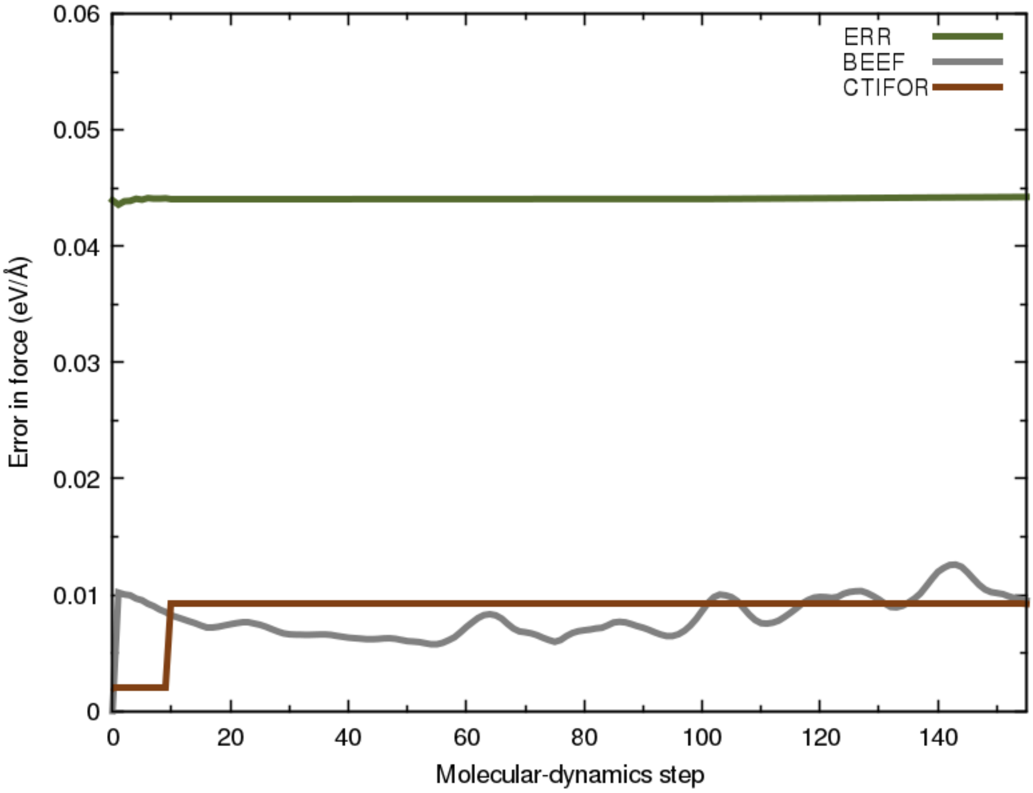

Figure S9. Error in the force field of the HOPG reduced O-doping structure during the "on-the-fly" machine learning simulation. The errors in the plot are shown at different stages of the training process. The evolution of the Bayesian error estimates of forces is shown in dark grey colour, the root mean squared error of the predicted forces is represented in dark olive green colour, and the current threshold for maximum Bayesian error estimation of the forces is depicted in sienna colour.

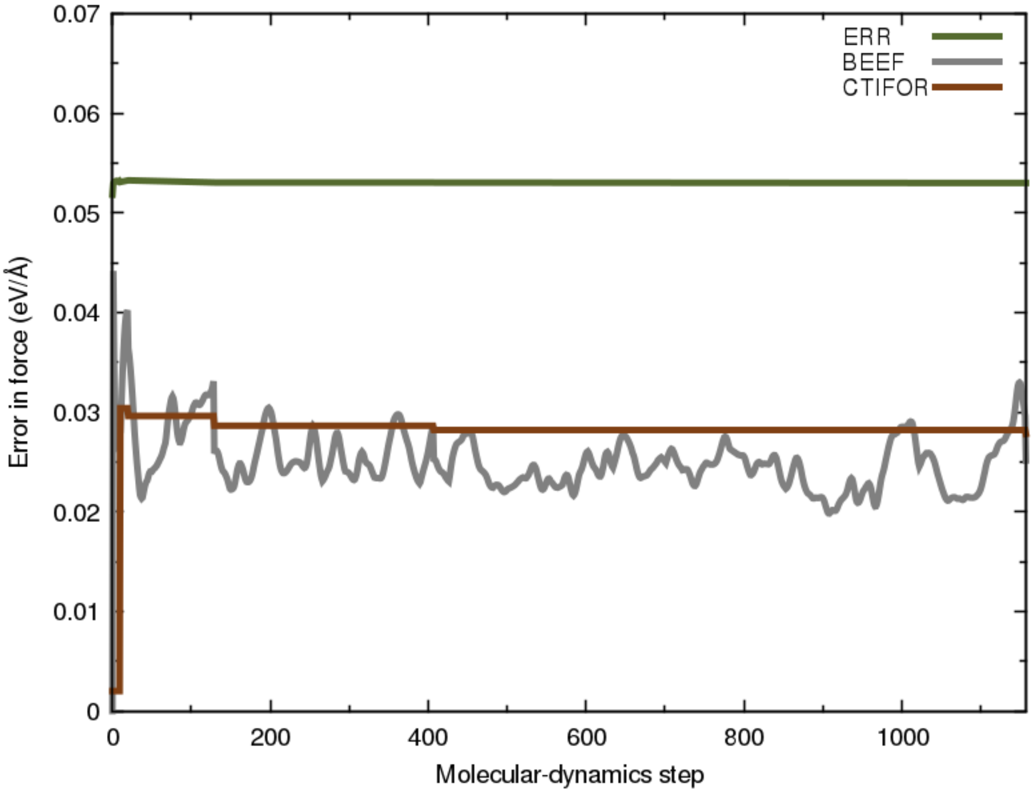

Figure S10. Error in the force field of the HOPG increased O-doping structure during the "on-the-fly" machine learning simulation. The errors in the plot are shown at different stages of the training process. The evolution of the Bayesian error estimates of forces is shown in dark grey colour, the root mean squared error of the predicted forces is represented in dark olive green colour, and the current threshold for maximum Bayesian error estimation of the forces is depicted in sienna colour.

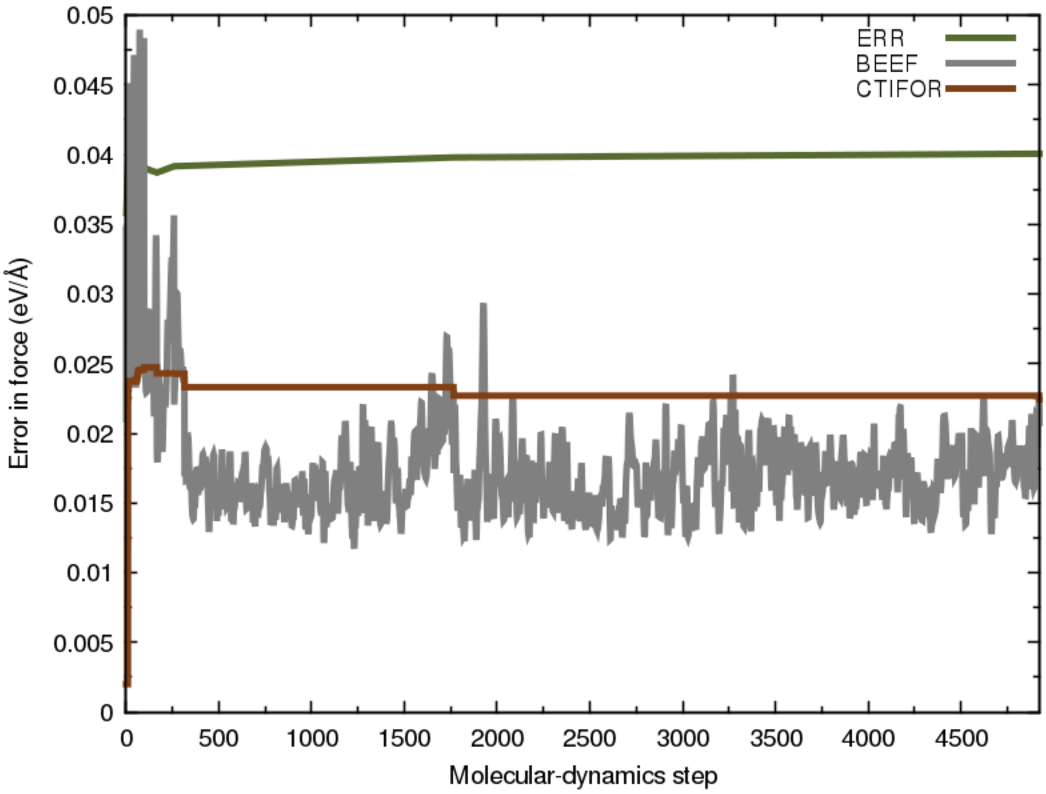

Figure S11. Error in the force field of the HOPG reduced S-doping structure during the "on-the-fly" machine learning simulation. The errors in the plot are shown at different stages of the training process. The evolution of the Bayesian error estimates of forces is shown in dark grey colour, the root mean squared error of the predicted forces is represented in dark olive green colour, and the current threshold for maximum Bayesian error estimation of the forces is depicted in sienna colour.

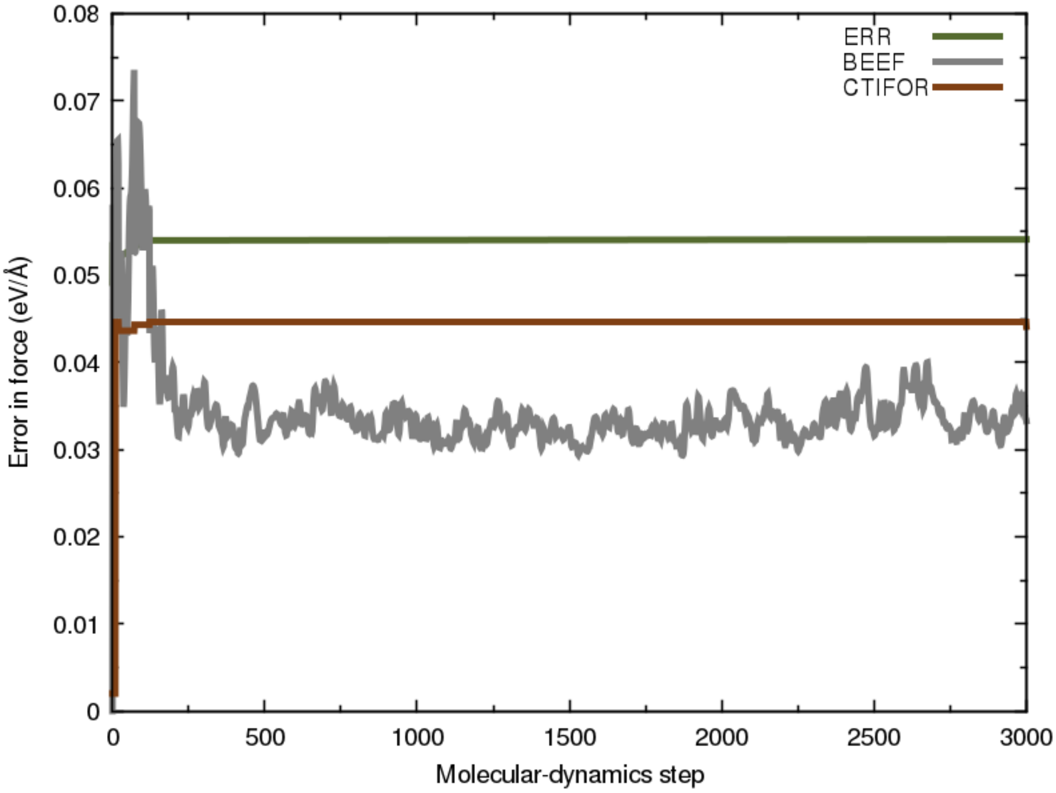

Figure S12. Error in the force field of the HOPG increased S-doping structure during the "on-the-fly" machine learning simulation. The errors in the plot are shown at different stages of the training process. The evolution of the Bayesian error estimates of forces is shown in dark grey colour, the root mean squared error of the predicted forces is represented in dark olive green colour, and the current threshold for maximum Bayesian error estimation of the forces is depicted in sienna colour.

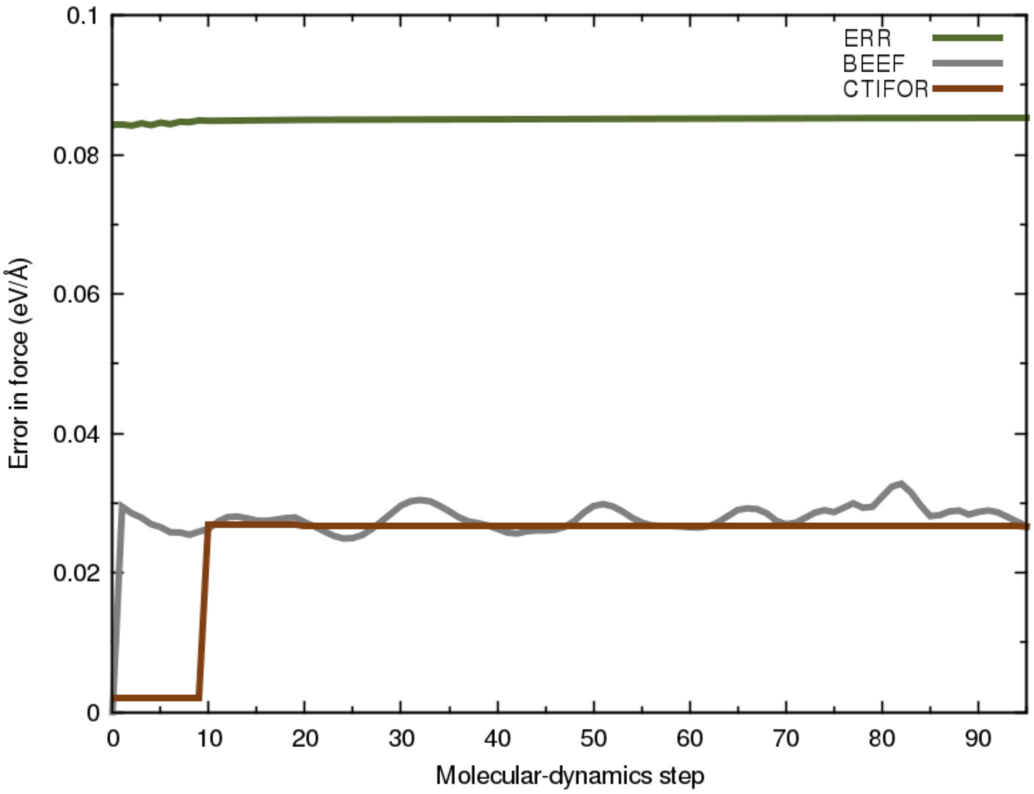

Figure S13. Error in the force field of the armchair graphene nanoribbons (GNRs) on HOPG structure during the "on-the-fly" machine learning simulation. The errors in the plot are shown at different stages of the training process. The evolution of the Bayesian error estimates of forces is shown in dark grey colour, the root mean squared error of the predicted forces is represented in dark olive green colour, and the current threshold for maximum Bayesian error estimation of the forces is depicted in sienna colour.

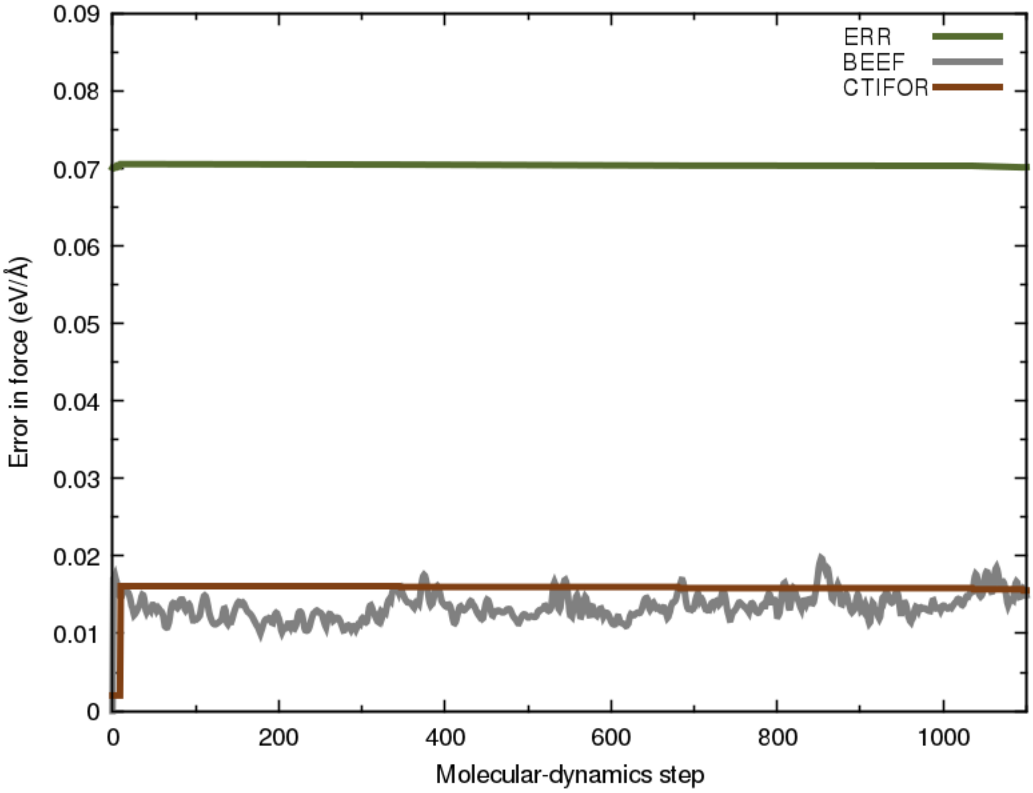

Figure S14. Error in the force field of the zigzag graphene nanoribbons (GNRs) on HOPG structure during the "on-the-fly" machine learning simulation. The errors in the plot are shown at different stages of the training process. The evolution of the Bayesian error estimates of forces is shown in dark grey colour, the root mean squared error of the predicted forces is represented in dark olive green colour, and the current threshold for maximum Bayesian error estimation of the forces is depicted in sienna colour.

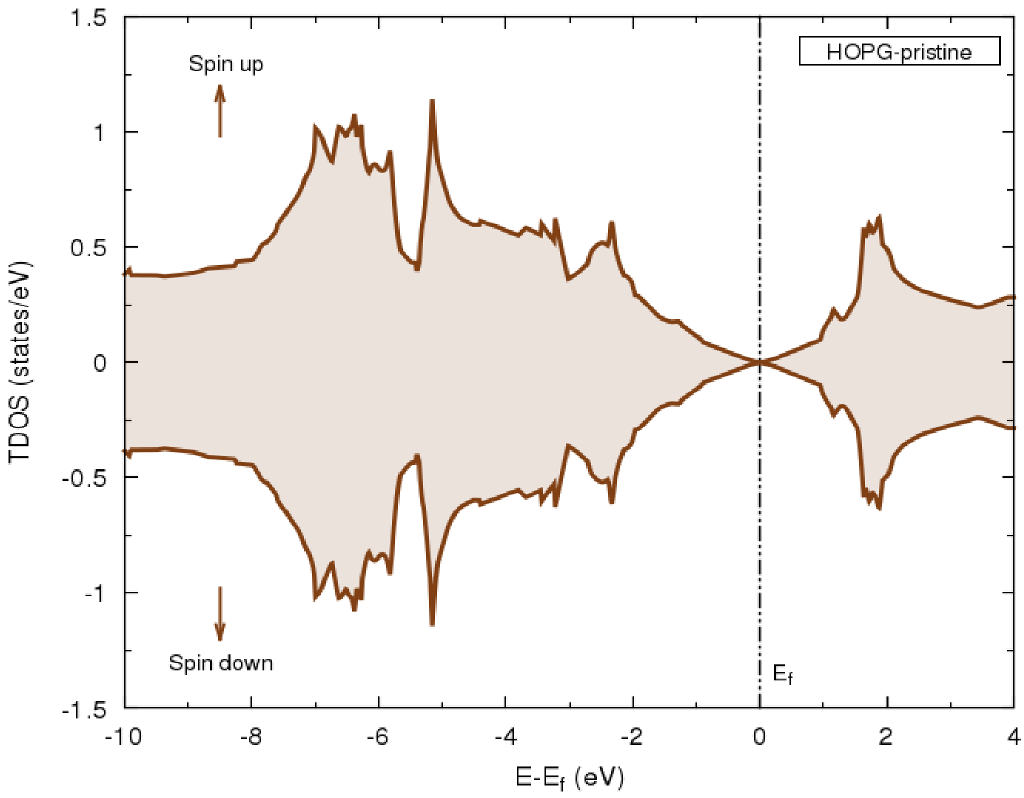

Figure S15. Calculated total density of state (TDOS) of pristine HOPG

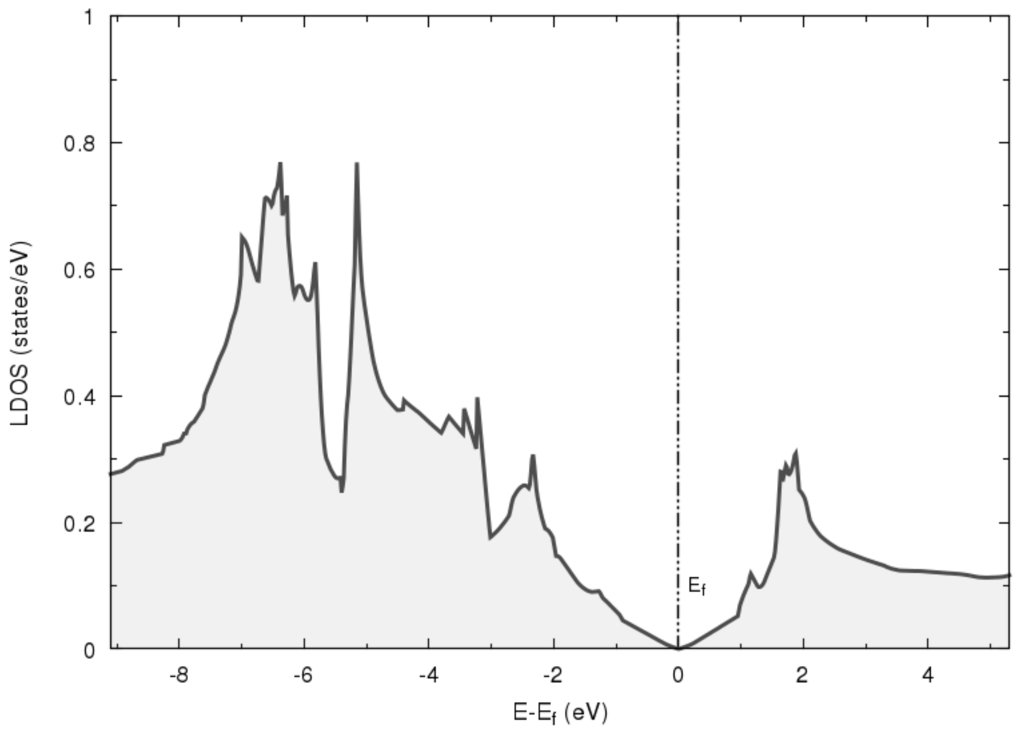

Figure S16. Calculated local density of state (LDOS) of pristine HOPG

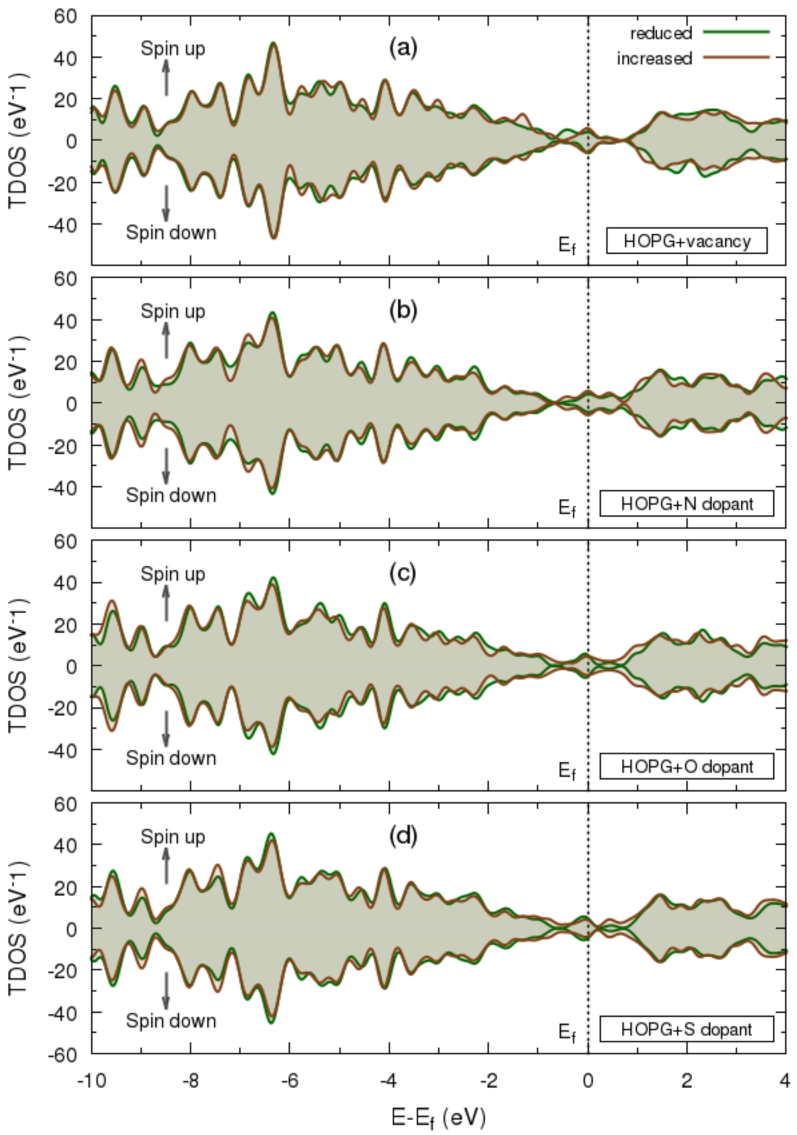

Figure S17. Calculated total density of states (TDOS) for HOPG with reduced and increased point defects, including (a) vacancy, (b) N dopant, (c) O dopant, and (d) S dopant.

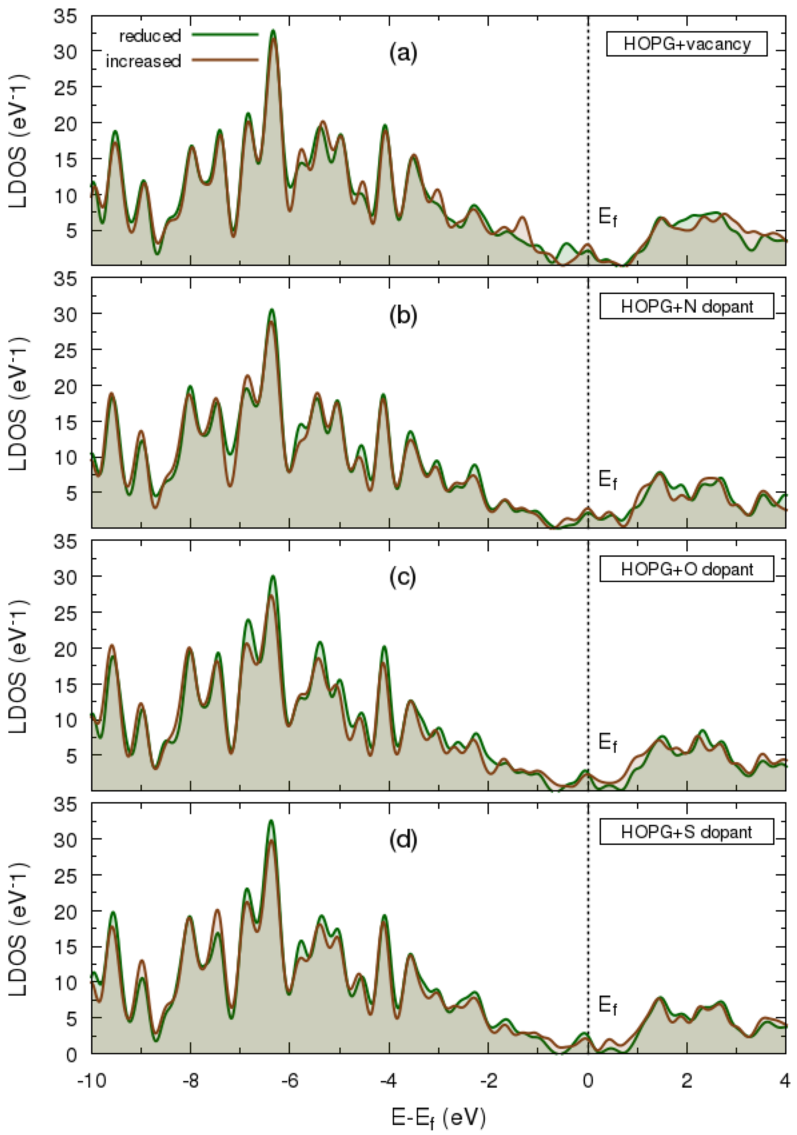

Figure S18. Calculated local density of states (LDOS) for HOPG with reduced and increased point defects, including (a) vacancy, (b) N dopant, (c) O dopant, and (d) S dopant.

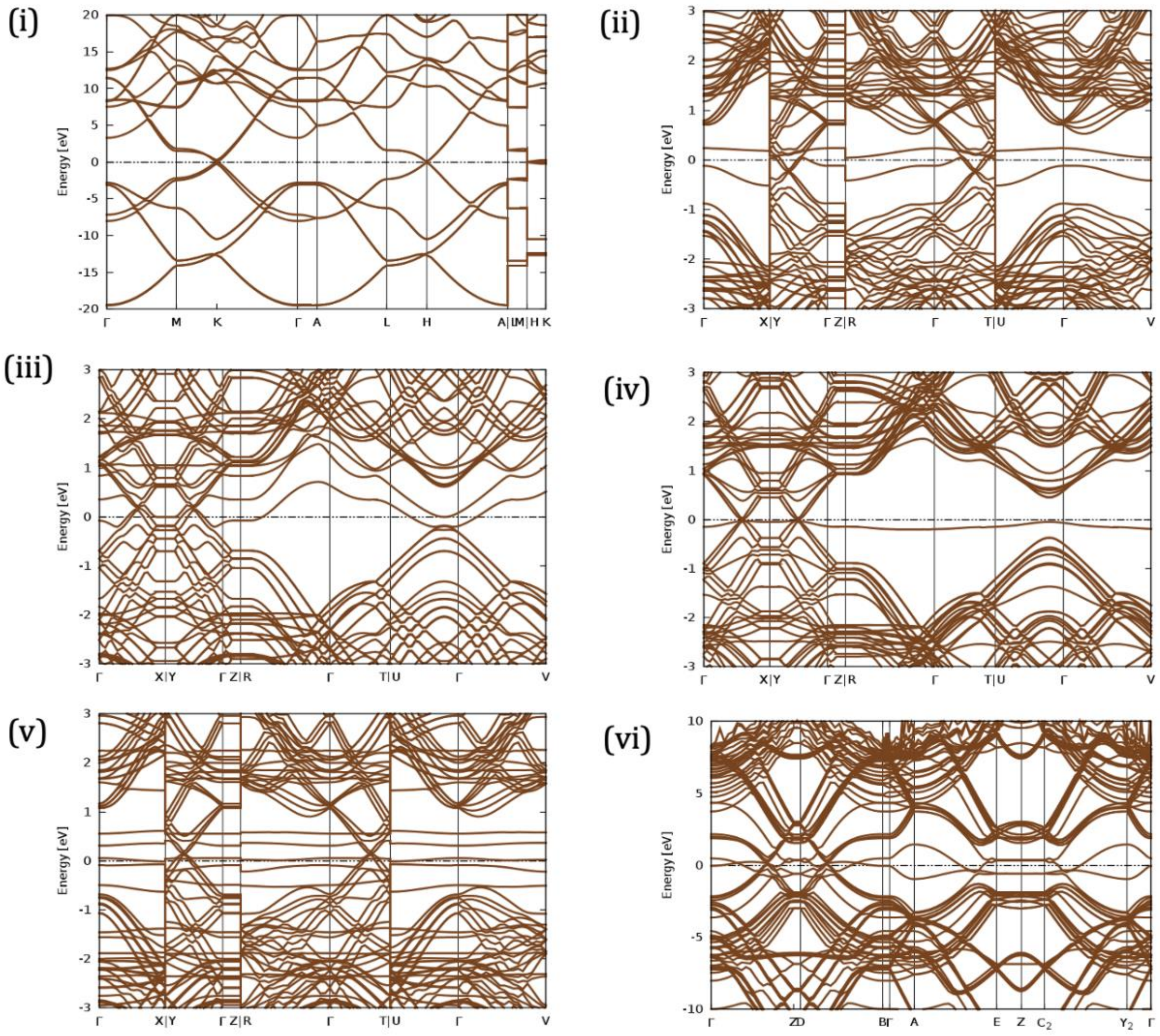

Figure S19. Electronic band structure of (i) pristine HOPG, (ii) HOPG with reduced vacancy defect, (iii) HOPG with reduced N dopant, (iv) HOPG with reduced S dopant, (v) armchair graphene nanoribbons (AGNRs) on HOPG, and (vi) zigzag graphene nanoribbons (ZGNRs) on HOPG.

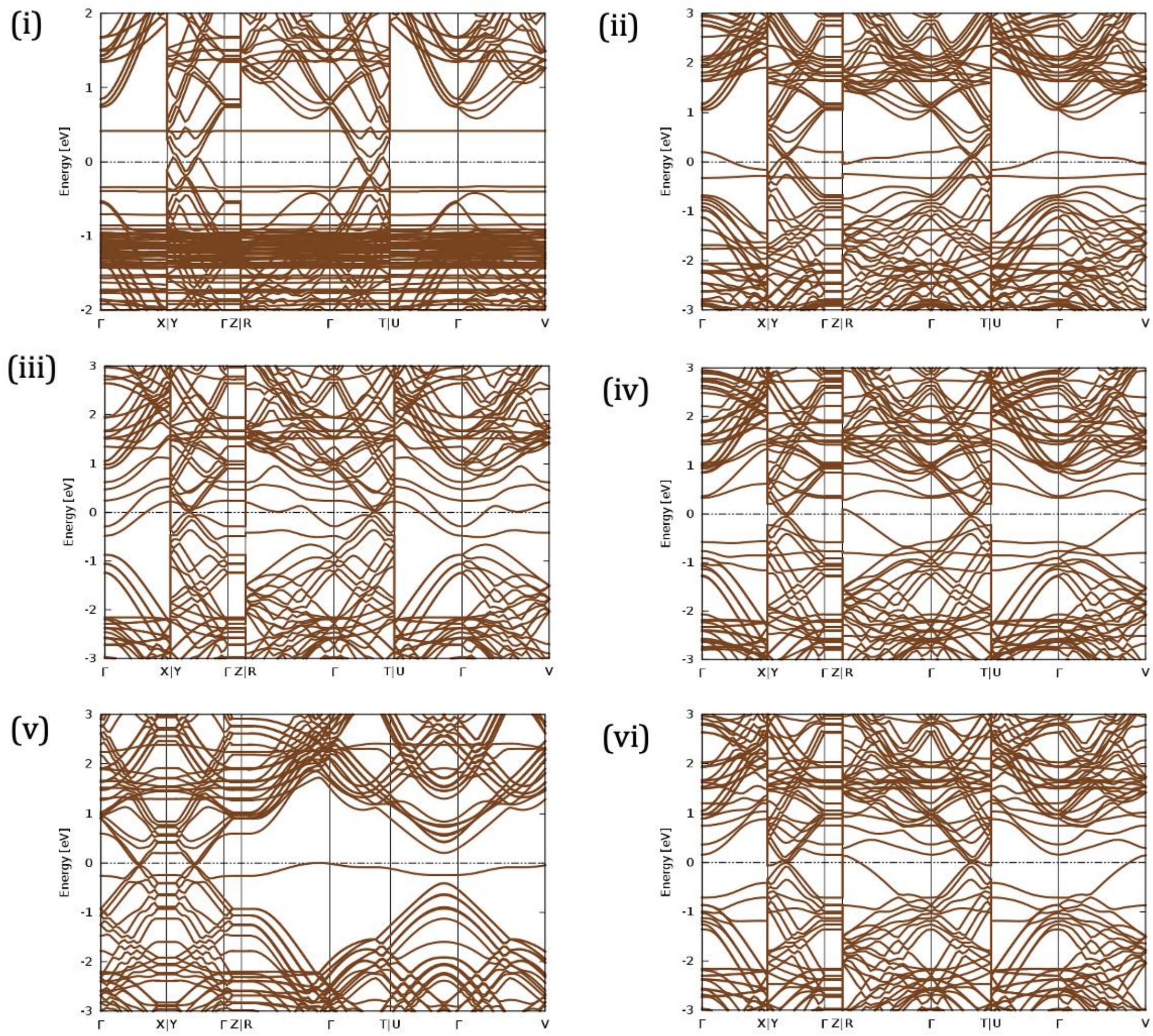

Figure S20. Electronic band structure of (i) HOPG-water interface, (ii) HOPG with increased vacancy defect, (iii) HOPG with increased N dopant, (iv) HOPG with increased S dopant, (v) HOPG with reduced O dopant, and (vi) HOPG with increased O dopant.

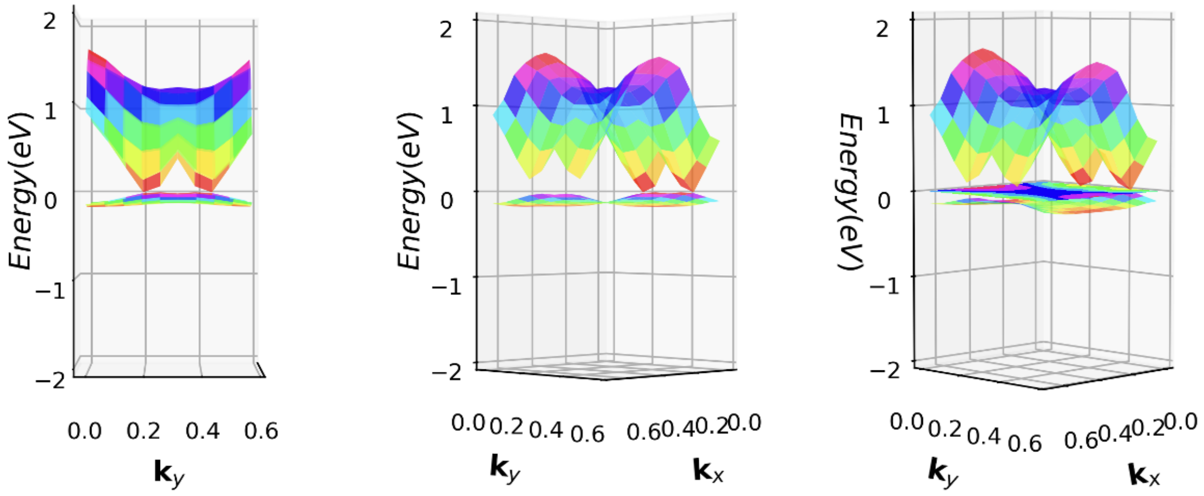

Figure S21. 3D band structure of HOPG with reduced S dopant.

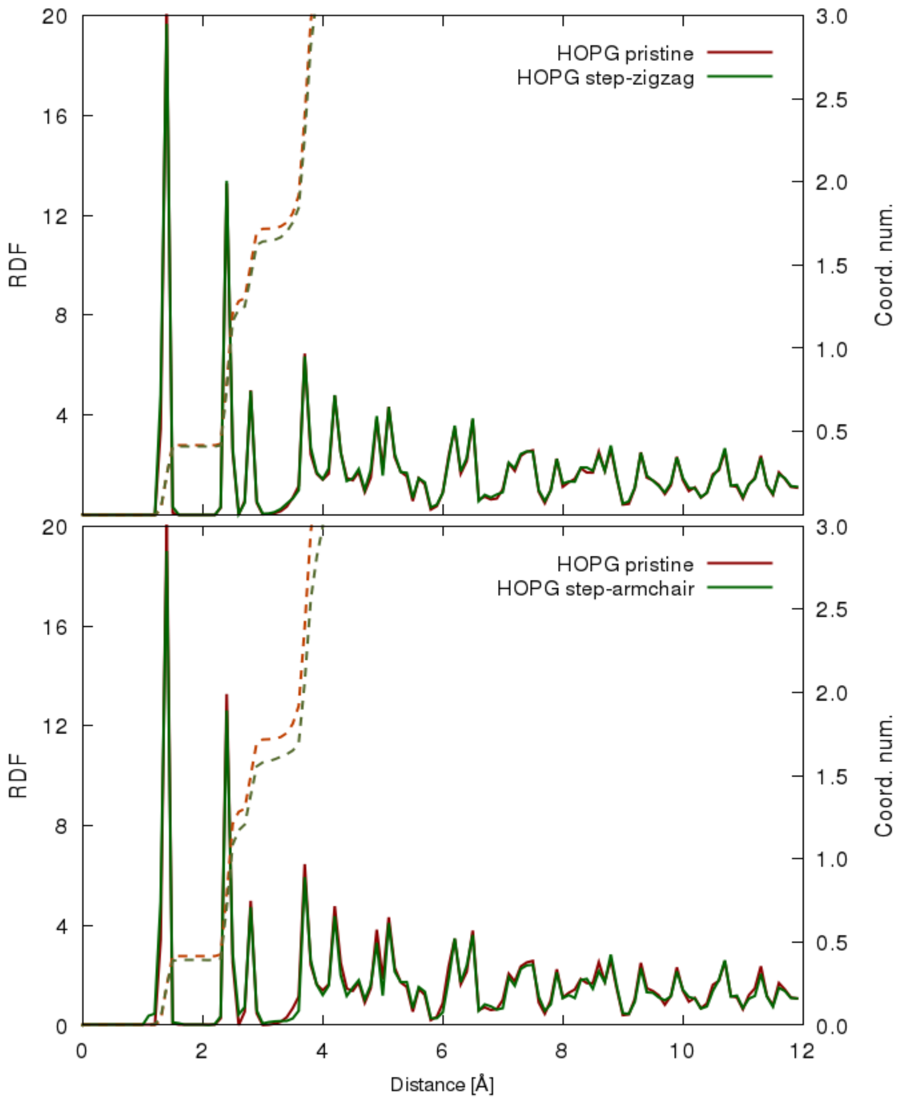

Figure S22. Radial distribution functions (RDF) detecting the interaction of zigzag graphene nanoribbons (ZGNRs) and armchair graphene nanoribbons (AGNRs) on HOPG. A comparison is made with the pristine HOPG surface structure.

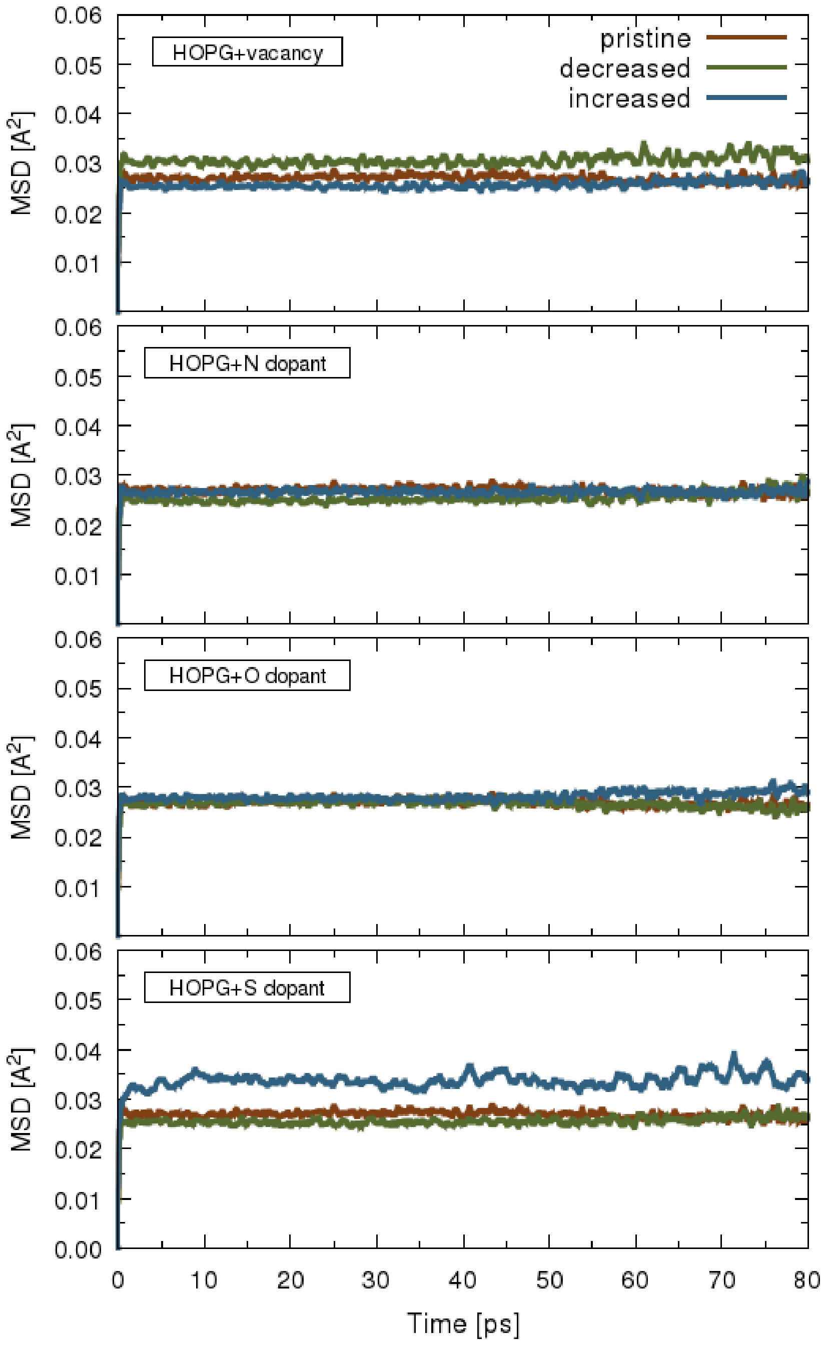

Figure S23. Mean squared displacement of HOPG with reduced and increased point defects, including (a) vacancy, (b) N dopant, (c) O dopant, and (d) S dopant. A comparison is made with the pristine HOPG.

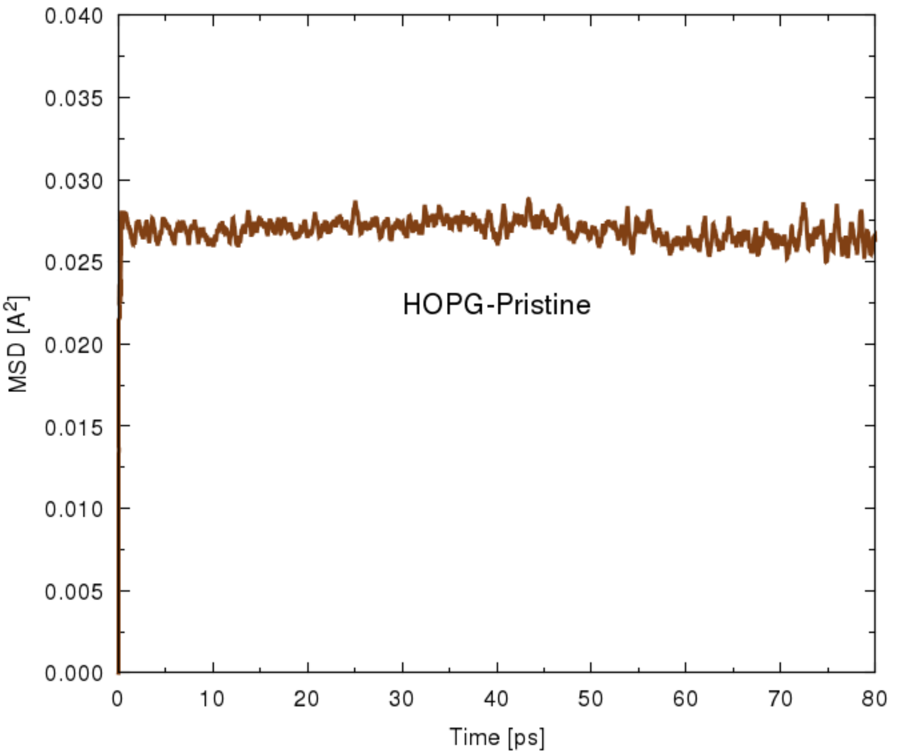

Figure S24. Mean squared displacement of pristine HOPG.

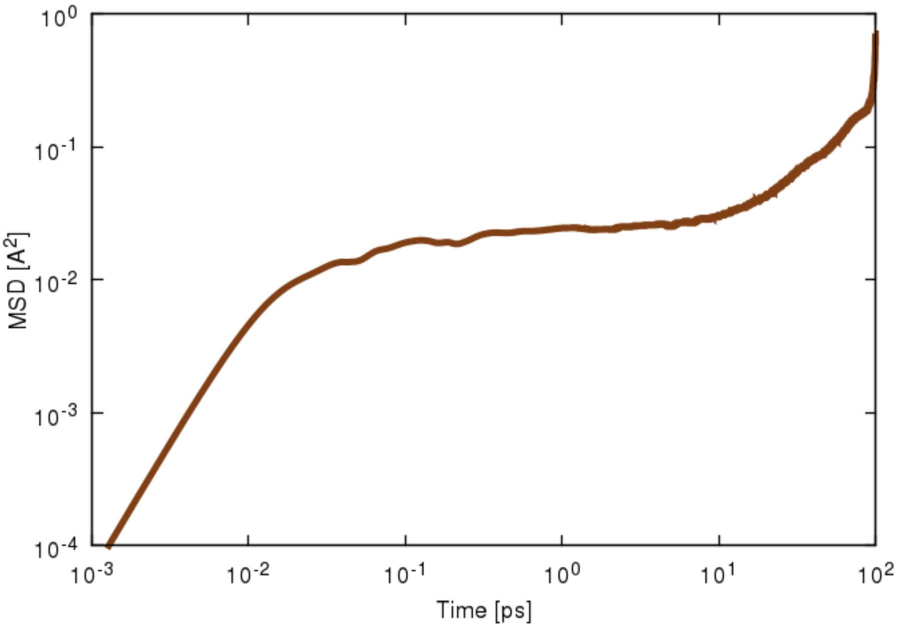

Figure S25. Mean squared displacement of HOPG surface at the HOPG-water interface shown in logarithm scale.

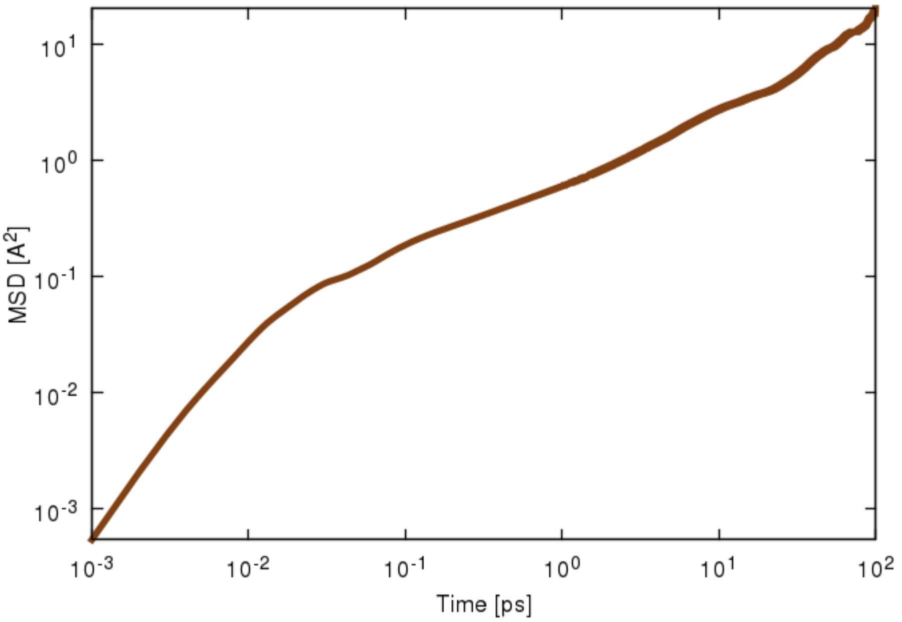

Figure S26. Mean squared displacement of water molecules at the HOPG-water interface shown in logarithm scale.

Table S1. Dynamical properties of pristine HOPG, vacancy and substitutional HOPG defect: mean squared displacement (MSD), Å^2; and diffusion coefficient, D.

		**MSD (Å^2)**	**D (cm^2/s)**
HOPG	pristine	0.0279	3.607E-08
vacancy defect	reduced	0.0321	3.998E-08
	increased	0.0269	3.455E-08
Substitutional defect	reduced N-doping	0.0276	3.404E-08
	reduced O-doping	0.0274	3.573E-08
	reduced S-doping	0.0266	3.434E-08
	increased N-doping	0.0276	3.563E-08
	increased O-doping	0.0296	3.756E-08
	increased S-doping	0.0343	4.176E-08

References

1. Bird, J. P. (2002). Semiconductors: An introduction. Encyclopedia of Materials: Science and Technology, 1–16. https://doi.org/10.1016/b0-08-043152-6/01854-4
2. Ibach, H., & Lüth, H. (2009). Solid-State Physics. An Introduction to Principles of Materials Science (4th ed.). Springer-Verlag https://doi.org/10.1007/978-3-540-93804-0
3. Lechner, C., Pannier, B., Baranek, P., Forero-Martinez, N. C., & Vach, H. (2016). First-Principles Study of the structural, electronic, dynamic, and mechanical properties of HOPG and diamond: Influence of Exchange–Correlation functionals and dispersion interactions. The Journal of Physical Chemistry C, 120(9), 5083–5100. https://doi.org/10.1021/acs.jpcc.5b10396
4. Setyawan, W., & Curtarolo, S. (2010). High-throughput electronic band Structure Calculations: Challenges and Tools. Computational Materials Science, 49(2), 299–312. https://doi.org/10.1016/j.commatsci.2010.05.010
5. Pantin, V., Avila, J., Valbuena, M. A., Esquinazi, P., Dávila, M. E., & Asensio, M. C. (2006). Electronic properties of high oriented pyrolitic Graphite: Recent discoveries. Journal of Physics and Chemistry of Solids, 67(1–3), 546–551. https://doi.org/10.1016/j.jpcs.2005.10.169
6. Yazyev, O. V. (2013). A guide to the design of electronic properties of graphene nanoribbons. Accounts of Chemical Research, 46(10), 2319–2328. https://doi.org/10.1021/ar3001487
7. Yang, L., Cohen, M. L., & Louie, S. G. (2008). Magnetic edge-state excitons in zigzag graphene nanoribbons. Physical Review Letters, 101(18). https://doi.org/10.1103/physrevlett.101.186401
8. Zheng, X. H., Rungger, I., Zeng, Z., & Sanvito, S. (2009). Effects induced by single and multiple dopants on the transport properties in zigzag-edged graphene nanoribbons. Physical Review B, 80(23). https://doi.org/10.1103/physrevb.80.235426
